# Transposon sequencing reveals *Burkholderia* gene fitness in a spaceflight-relevant plant-pathogen interaction

**DOI:** 10.1128/aem.01941-25

**Published:** 2026-01-13

**Authors:** Anya Volter, Jessica Atkin, Aaron Curry, Anirudha Dixit, Rachel Tucker, Hannah Roberts, Mary Hummerick, Elison B. Blancaflor, Aubrie O'Rourke

**Affiliations:** 1 NASA Space Technology Graduate Research Opportunities (NSTGRO) Visiting Technologist Experience (VTE), Kennedy Space Center 53410 , Merritt Island, Florida, USA; 2 NASA Kennedy Space Center Office of STEM Engagement (OSTEM) Intern Program, Kennedy Space Center 53410 , Merritt Island, Florida, USA; 3 Astrion, LASSO II, Kennedy Space Center, Merritt Island, Florida, USA; 4 Aetos Systems Inc., LASSO II, Kennedy Space Center, Merritt Island, Florida, USA; 5 Bennett Aerospace, LASSO II, Kennedy Space Center, Merritt Island, Florida, USA; 6 Noetic Strategies, Inc., LASSO II, Kennedy Space Center, Merritt Island, Florida, USA; 7 NASA Exploration Research and Technology, Kennedy Space Center 53410 , Merritt Island, Florida, USA; The University of Tennessee Knoxville, Knoxville, Tennessee, USA

**Keywords:** *Burkholderia*, Nudix hydrolase, *Solanum lycopersicum*, *Fusarium oxysporum*, transposon sequencing

## Abstract

The spaceflight environment imparts unique selective pressures on the plants and microbes of plant growth chambers on the International Space Station (ISS), which generally manifests through genetic signatures associated with a heightened response to stress. Terrestrially, a baseline understanding of the gene fitness response for any plant growth-promoting microbe when in a tripartite relationship with host and pathogen is currently unknown and is important to characterize before closed-environment spaceflight implementation. To that end, this study evaluated the behavior of an ISS plant habitat isolate of *Burkholderia contaminans* as tomato seeds transitioned to seedlings and assessed gene fitness during challenge with *Fusarium oxysporum* f. sp. *lycopersici* (FOL), the causal agent of Fusarium wilt. Using a seed film delivery method vetted for spaceflight, *B. contaminans* was applied to *Solanum lycopersicum* cv. Red Robin seeds. Green fluorescent protein (GFP)-tagged *B. contaminans* was primarily found to localize at the shoot-root junction and was detected on shoots. Upon FOL challenge, *B. contaminans* population levels remained stable, and despite harboring antifungal and plant growth-promoting capacity, these properties were not conferred in response to FOL in the tissue culture environment. To probe mechanisms underlying the bacterial-fungal interaction between *B. contaminans* and FOL in the tomato root zone, a genome-wide transposon mutant library was developed for the *B. contaminans* isolate. Transposon sequencing (Tn-Seq) analysis revealed that the type II secretion system (T2SS) was critical for root zone establishment, whereas a Nudix hydrolase was specifically important for responding to FOL infection and provided further confirmation that antifungal and siderophore-producing gene clusters were not.

**IMPORTANCE:**

This study is the first to evaluate the genetic fitness of a *Burkholderia contaminans* International Space Station (ISS) isolate in the plant root zone in association with the obligate pathogen *Fusarium oxysporum* f. sp. *lycopersici* (FOL). This isolate of *B. contaminans* establishes in the tomato root zone, does not confer plant growth promotion in tissue culture, but is persistent in the tomato root zone when challenged with FOL through stress-adaptation mechanisms rather than direct antifungal antagonism. The response of *B. contaminans* in the host root zone when in the presence of the pathogen suggests the microbe is primed to counter stress, which may further confer an advantage in the spaceflight environment.

## INTRODUCTION

Spaceflight habitats are closed ecosystems where plants, microbes, and crew coexist in tightly coupled environments. The Vegetable Production System (Veggie) unit on the International Space Station (ISS) consists of a light-emitting diode panel overtop a plant growth area with an enclosure to separate the plants from crew quarters (1). The unit shares its atmosphere and microbiome with the greater crew habitat of the ISS. In addition, Veggie utilizes water from the ISS potable water dispenser (PWD) for crop watering needs. Accordingly, microbial monitoring efforts in the closed environment of the ISS have repeatedly identified *Burkholderia contaminans* both in the PWD (2) and in plant growth chambers. Members of the *Burkholderia cepacia* complex (BCC), such as *B. contaminans*, possess large genomes with extensive metabolic redundancy, enabling survival in both nutrient-rich and oligotrophic conditions (3). Many strains produce several secondary metabolites, including antifungal compounds, positioning them as promising candidates for crop protection against fungal pathogens in resource-limited environments (4).

The space crop community has looked to the plant microbiome to provide a countermeasure to buffer plants from opportunistic pathogens when engineered environmental controls go off nominal (1). Prior work has demonstrated that *B. contaminans* isolates from the ISS PWD can inhibit fungal growth and are no more cytotoxic to macrophages than terrestrial isolates (2). This capacity for *B. contaminans* to inhibit fungal growth is of interest to space crop production efforts, given a 2015 occurrence where a subset of Veggie-grown Zinnia flowering plants on the ISS were infected by the opportunistic growth of *Fusarium oxysporum* (5). The event, attributed to a fan malfunction, was contained. No occurrences have been observed since the *Fusarium* incident, and in 2024, several experiments on the ISS successfully showed disease-free growth of tomato.

To date, no obligate plant pathogens have been detected on the ISS. However, obligate pathogens do present a risk for exploration. Specifically, *Fusarium oxysporum* f. sp. *lycopersici* (FOL), the causative agent of Fusarium wilt, is responsible for at least 14% of tomato economic product loss and is the fifth most important plant pathogenic fungus worldwide (6). The common practice for thwarting a disease outbreak of FOL is by field rotation of crops (7). In the resource-limited spaceflight or extraterrestrial surface environments on the Moon and Mars, this rotation method may not be a feasible solution. Therefore, there is a need to pursue alternative methods to avoid catastrophic disease outbreaks in space agricultural settings. The use of beneficial plant microbes is a promising tool for Earth agriculture (8), and this study investigates if similar approaches are applicable to space.

Transposon sequencing (Tn-Seq) provides a powerful approach to dissect gene fitness under selective pressures. Transposon sequences are DNA sequences that can move around and insert into the genome with the help of a transposase. This process has been manipulated as a molecular biology tool to introduce the Tn5 transposon and disrupt genes in a genome to create libraries of mutants. The mutants within a Tn-Seq library that survive when placed under an environmental challenge have interruptions in genes that would exhibit negative or neutral fitness in the wild type. The mutants that decrease or fully disappear after selection are considered to promote fitness or are essential for fitness in the wild type. Tn-Seq experiments in *Burkholderia* to date have focused on the human pathogenic species of *B. thailandensis* (9), *B. cenocepacia* (10), and *B. pseudomallei* (11), the plant colonizing *B. vietnamiensis* (12), the antifungal properties of *B. seminalis* (13, 14), and the *Lagria villosa* beetle symbiont, *B. gladioii* (15). These Tn-Seq studies across the genus have linked secretion systems (e.g., T6SS in pathogens; T2SS/T3SS in symbionts) to host interactions, while also revealing context-dependent deployment of secondary metabolism and stress response pathways. To date, no genome-wide fitness study has assessed the plant, bacterium, and fungal pathogen tripartite interaction.

*In vivo* evidence demonstrated that a strain of *B. contaminans* could act as a plant growth-promoting (PGP) microbe when applied to tomato in the presence of FOL by providing a 20%–50% growth advantage (16). Therefore, in this study, we sought to evaluate if an isolate of *B. contaminans* from the ISS Veggie plant growth unit could provide protection to the space crop, Red Robin tomato, when challenged with FOL. The metabolic repertoire of *Burkholderia* has allowed the genera to present as plant beneficial microbes, plant pathogens, and even as human opportunistic pathogens (17). While it is known that *B. contaminans* in the ISS ecosystem has not caused any opportunistic infections in crew, it remains important to understand the behavior of *B. contaminans* in higher concentrations if it were to be seriously considered as a plant beneficial microbe in the closed spaceflight environment. A green fluorescent protein (GFP) reporter line of the ISS *B. contaminans* isolate was developed to monitor its movement and understand the mechanisms by which it colonizes the root and shoot zone. Furthermore, a transposon library was developed and implemented to determine the gene fitness of the ISS *B. contaminans* isolate upon FOL challenge.

## RESULTS

### *Burkholderia* tracking

**Fig 1:**
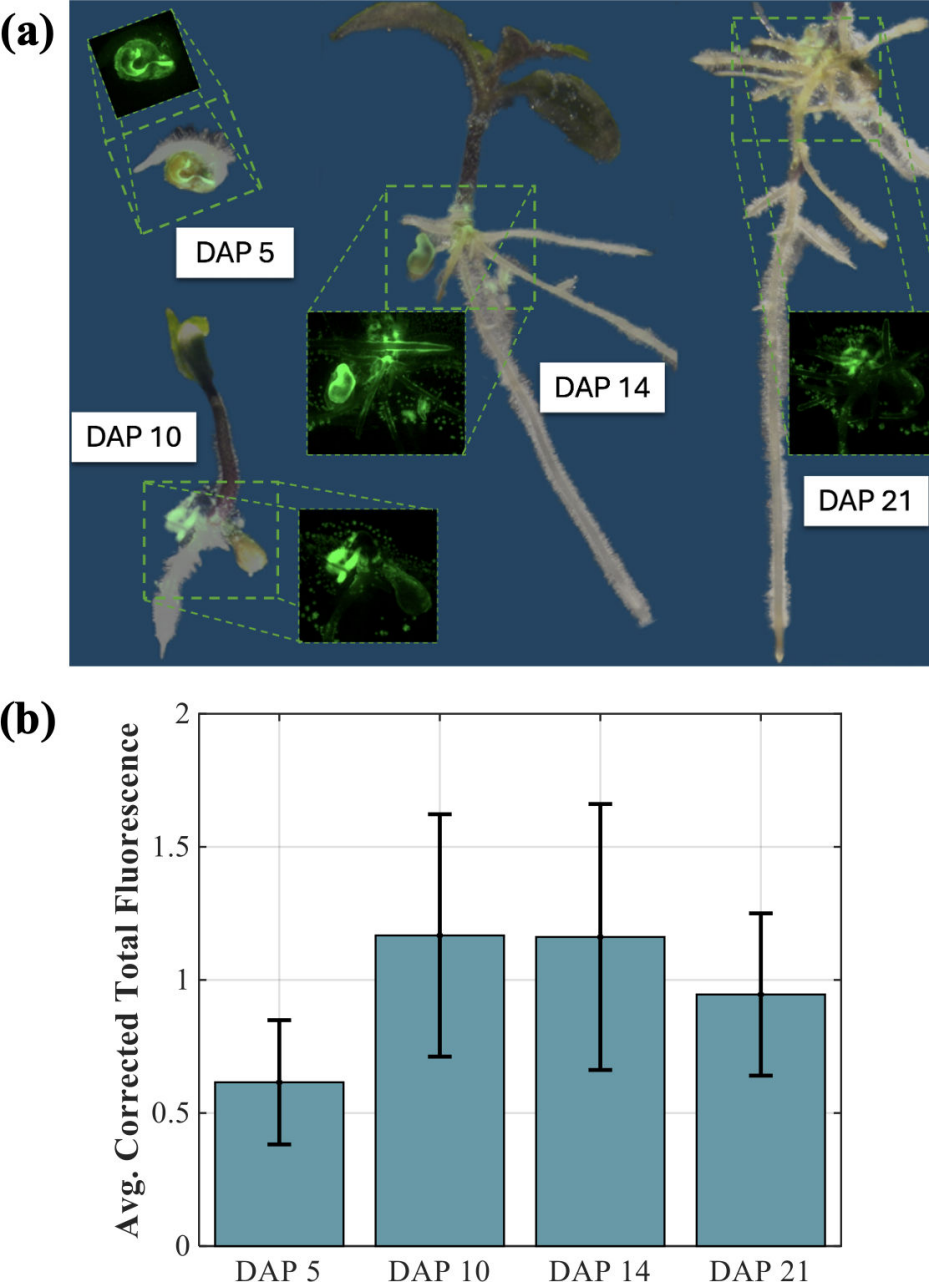
(**a**) Bright-field and fluorescence imaging overlay of *B. contaminans* containing a GFP-reporter plasmid at DAP 5, 10, 14, and 21. (**b**) Corresponding average corrected total fluorescence from images.

Bright-field and fluorescence imaging of *B. contaminans* containing a GFP-reporter plasmid showed concentrated growth around the seed coat that spread to the root surface over time (Fig. 1a). Quantification of GFP-expressing *B. contaminans* showed the highest intensities on day after planting (DAP) 10 and 14 (Fig. 1b).

### FOL inhibition and plant challenge assays

**Fig 2:**
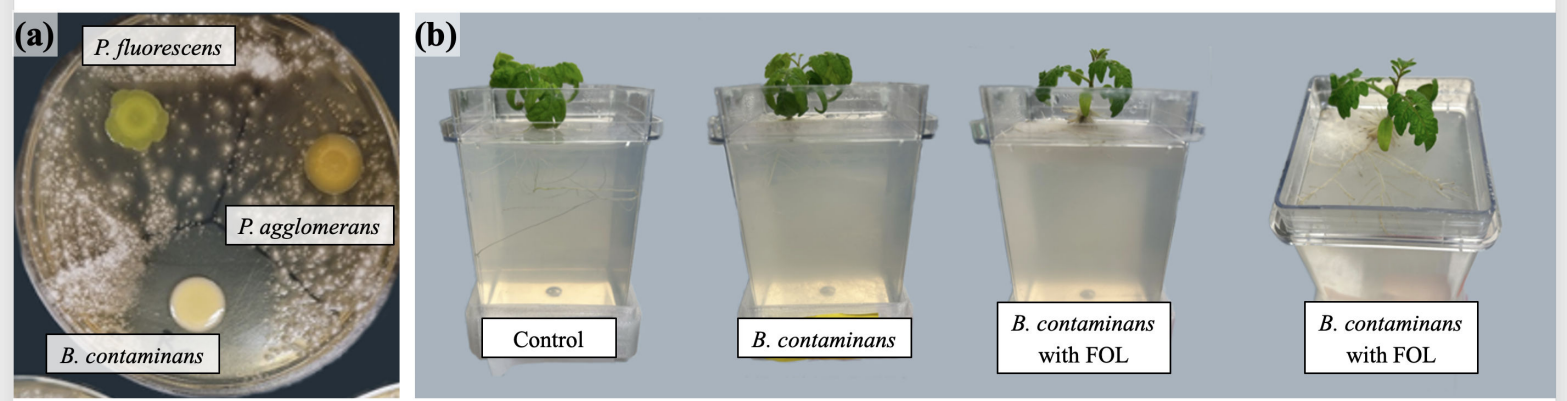
(**a**) Zone of inhibition for *B. contaminans*, *Pseudomonas fluorescens*, and *Pantoea agglomerans* against FOL on PDA. (**b**) Magenta vessels filled with phytogel harboring a plant at 28 days, with control, *B. contaminans* inoculation, and *B. contaminans* inoculation plus FOL challenge.

**Fig 3:**
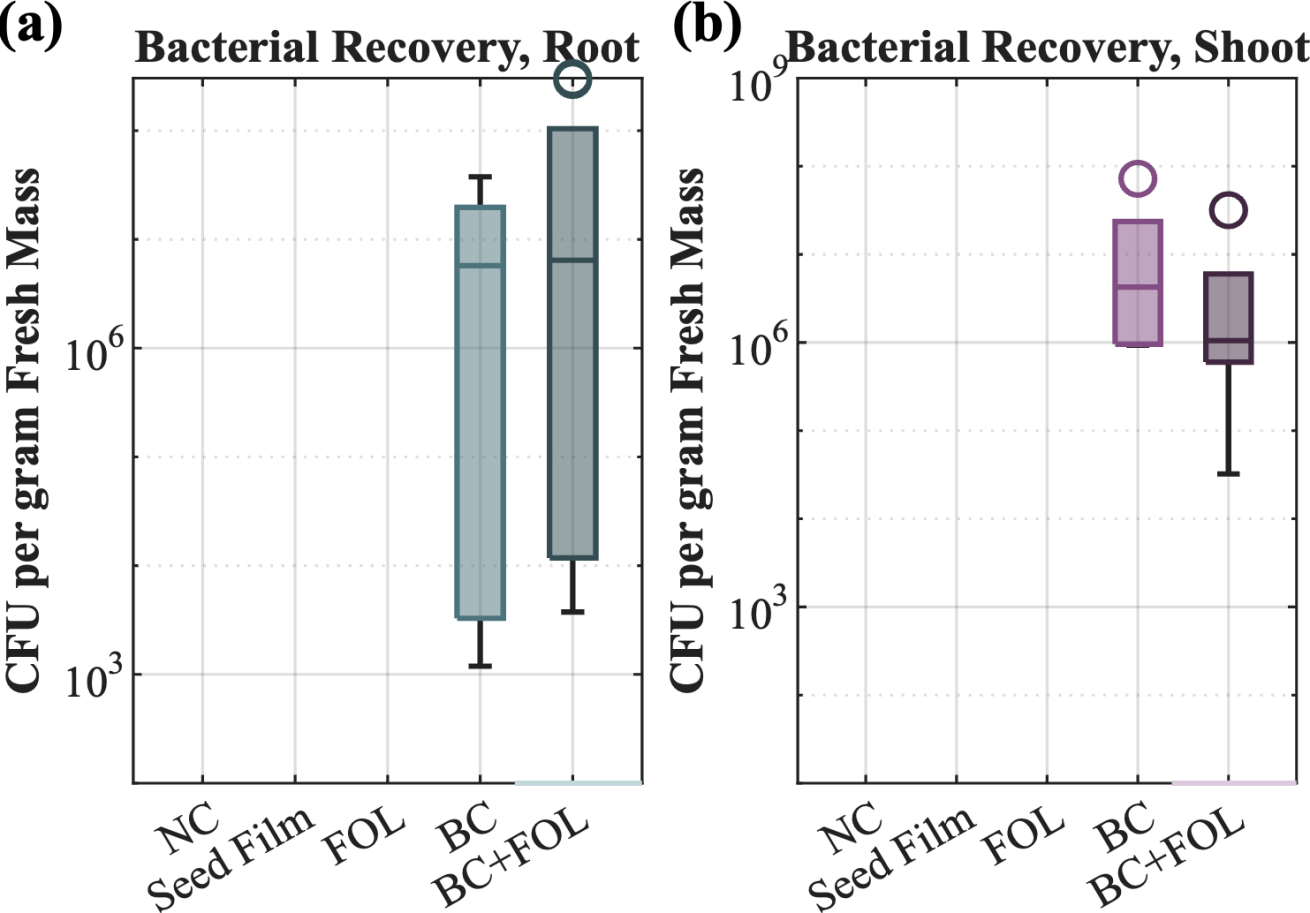
Plate count data for fluorescent *B. contaminans* recovered from tomato root (**a**) and shoot (**b**) samples grown in magenta jars study. Bars represent the min and max values, and the horizontal line within the box is the median. *N* = 9 for negative control (NC), seed film control, and FOL control; *N* = 6 for *B. contaminans* control (“BC”) and *B. contaminans* plus FOL (“BC+FOL”).

**Fig 4:**
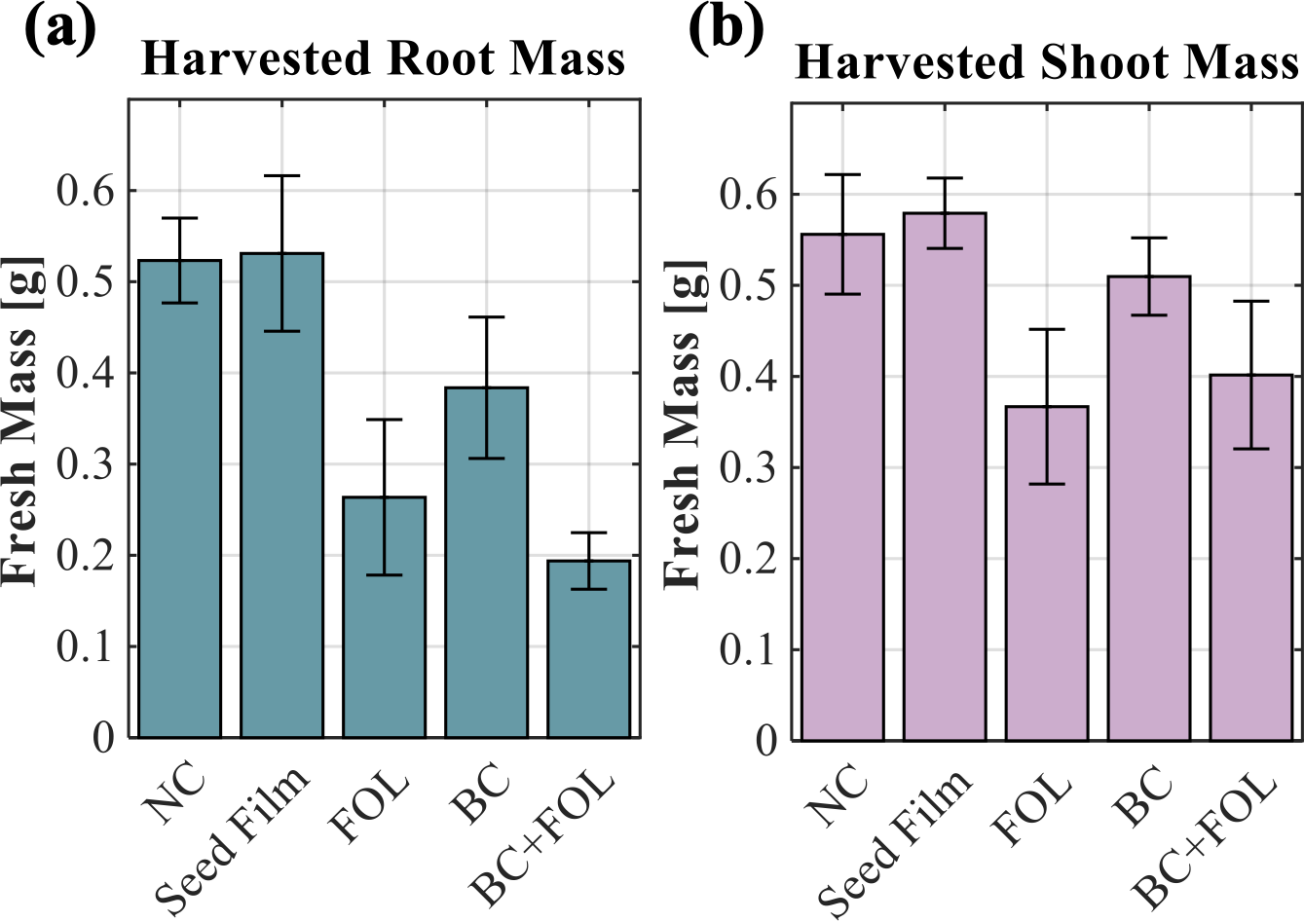
Average mass data for harvested tomato plant roots (**a**) and shoots (**b**). Bars represent the standard error of the mean. *N* = 9 for negative control (NC), seed film control, and FOL control; *N* = 6 for *B. contaminans* control (“BC”) and *B. contaminans* plus FOL (“BC+FOL”).

A zone of inhibition assay was carried out for the *B. contaminans* strain in co-culture with FOL. The zone of inhibition for *B. contaminans* was greatest against FOL on potato dextrose agar (PDA) with an average clearance zone of 100 mm (Fig. 2a). For comparison, *Pseudomonas fluorescens* and *Pantoea agglomerans* showed little to no inhibition (Fig. 2a). To assess biocontrol potential *in planta*, tomato seeds were coated with a seed film containing 10^8^ CFU/mL *B. contaminans* and subsequently challenged with FOL in magenta jars (Fig. 2b). GFP-tagged bacteria were successfully recovered from inoculated plants, with average recovery levels of one log lower than the initial inoculum (Fig. 3). Notably, bacteria were detected not only in roots but also in the aerial tissues, including edible biomass, confirming vertical migration from seed to shoot (Fig. 3a and b). Due to the plant processing method, the isolated bacteria could have originated from both within and from the surfaces of the sampled plant tissues. When evaluating plant growth promotion in the classical sense, no statistically significant difference in shoot or root biomass was observed between *B. contaminans*-treated plants and untreated controls (Fig. 4a and b). *Burkholderia* plus FOL-challenged plants (“BC+FOL”) displayed reduced root biomass relative to the two conditions of FOL alone (“FOL”) and *B. contaminans* alone (“BC”). Roots from “BC+FOL” were friable and fragile, in contrast to the morphology of healthy controls (Fig. 2b). Similarly, “FOL” was infected without *B. contaminans* and also displayed fragile roots.

### Tn-Seq input library and T0 profiles

A Tn5-based mutant library of *B. contaminans* was constructed to screen for genes involved in tomato plant colonization and fungal interaction. High-throughput sequencing was used to determine the coverage of insertion sites across the genome. For the input library, 86,616 insertion sites were identified in 7,735 genes of the 7,788 total genes. Insertion sequencing reads covered 89% of Chromo1, 80% of Chromo2, 77% of Chromo3, and 92% of Chromo4 in the *B. contaminans* genome. The 53 genes without insertion sequences were deemed essential for survival, where a mutation in the gene is lethal to growth. Gene Ontology (GO) enrichment classified these essential genes as ribosomal, translational regulation, and DNA binding. Following an outgrowth in nutrient-rich media (T0), insertion sites decreased to 56,360 (T0TnA: 67,586; T0TnB: 40,474; T0TnC: 61,021), disrupting 6,916 genes with sequencing reads covering 70% of Chromo1, 47% of Chromo2, 43% of Chromo3, and 37% of Chromo4. The 872 genes without insertion sequences were essential for the viability of *B. contaminans* in rich media. These genes were involved in lipid biosynthesis and metabolism, nucleoside, and protein metabolic processes. This observed 11.2% proportion of the genome found to be essential in the rich media environment is on the order of magnitude observed in *B. vietnamiensis* and *Paraburkholderia kururiensis* (12).

### Selective pressures in tissue culture conditions

**Fig 5:**
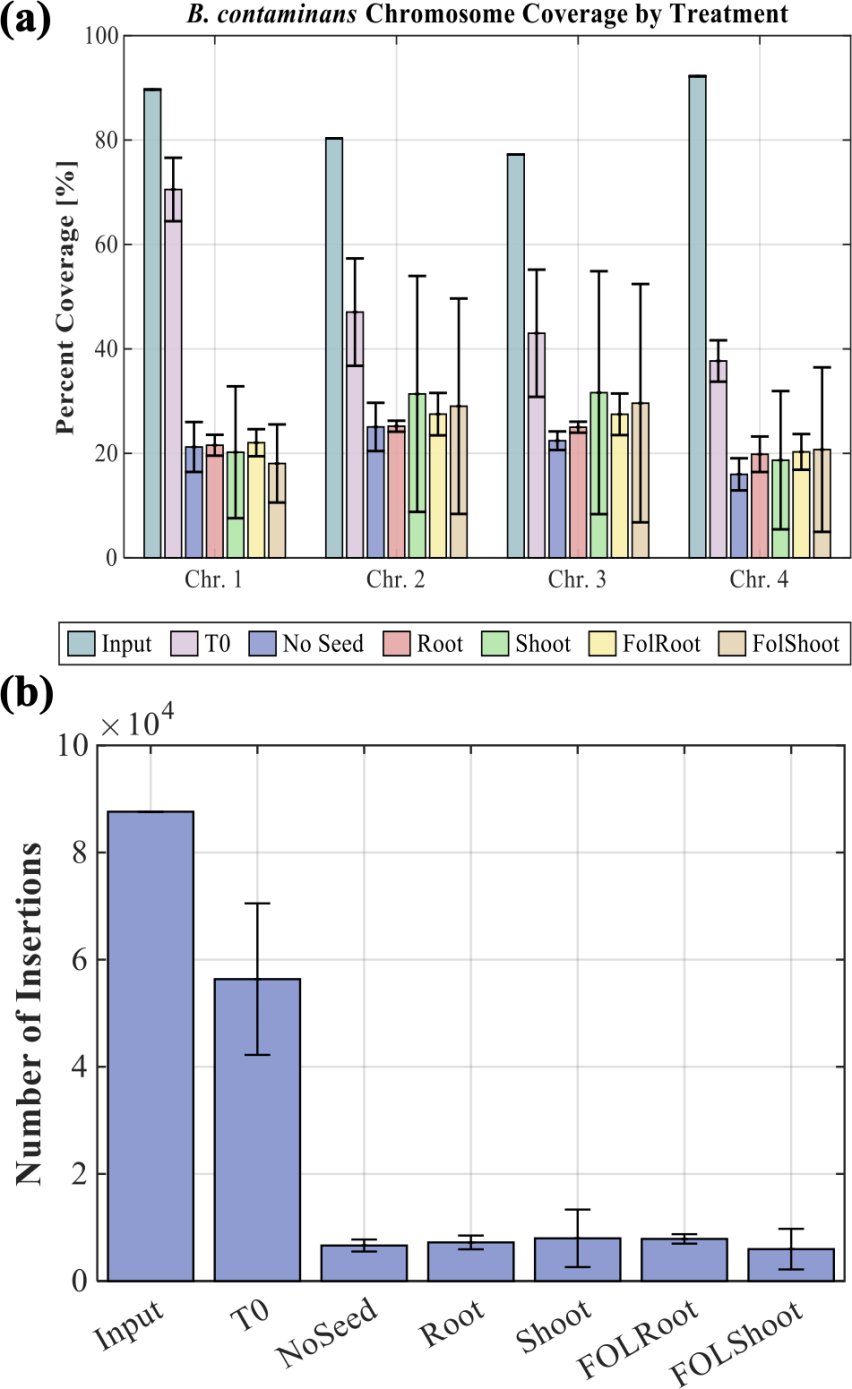
(**a**) Percent coverage of the *B. contaminans* genome by transposon insertion sites. (**b**) Transposon insertions detected per Tn-Seq condition.

To assess colonization *in planta*, the T0 library was applied to seed films under three conditions: (i) Tn-Seq library only (“No Seed”), (ii) Seeded tomato with Tn-Seq library (“Root” or “Shoot” recovery), (iii) Seeded tomato + Tn-Seq library + FOL challenged (“FOLRoot” or “FOLShoot” recovery). Transposed bacteria harvested from these conditions after 28 days of growth were processed and sequenced. Insertion mapping revealed reduced genome coverage across all conditions, reflecting selective pressures of the low-nutrient environment (Fig. 5a and b). The triplicates of the No Seed libraries showed an average of 6,633 unique insertion sites, where read coverage dropped to 21% of Chromo1, 25% of Chromo2, 22% of Chromo3, and 15% of Chromo4 for a total of 4,896 mutated genes. The triplicates of the Root libraries showed an average of 7,213 unique insertion sites, where average read coverage dropped to 21% of Chromo1, 25% of Chromo2, 25% of Chromo3, and 20% of Chromo4 for a total of 5,090 mutated genes. The triplicates of the Shoot library showed an average of 7,977 unique insertion sites, where average coverage dropped to 20% of Chromo1, 31% of Chromo2, 31% of Chromo3, and 19% of Chromo4 for a total of 3,089 mutated genes. The triplicates for the FOL Root libraries showed an average of 7,867 unique insertion sites, where average read coverage dropped to 22% of Chromo1, 28% of Chromo2, 27% of Chromo3, and 20% of Chromo4 for a total of 5,273 mutated genes. The triplicates for the FOL Shoot libraries showed an average of 5,960 unique insertion sites, where average read coverage dropped to 18% of Chromo1, 29% of Chromo2, 23% of Chromo3, and 15% of Chromo4 for a total of 4,207 mutants. The number of essential genes reflects the number of genes with a read count of zero for any one of the three biological replicates per condition. The essential gene number shows an inverse relationship to the insertion number per condition (Table 1).

**TABLE 1:** Essential gene count per condition as determined by Tn-Seq

	Essential genes (a count of 0 reads in any one of three biological replicates)
Input library	52
T0	872
No Seed	2,891
Root	4,698
Shoot	2,697
FOL Root	3,579
FOL Shoot	2,517

### Shared and condition-specific fitness determinants

**Fig 6:**
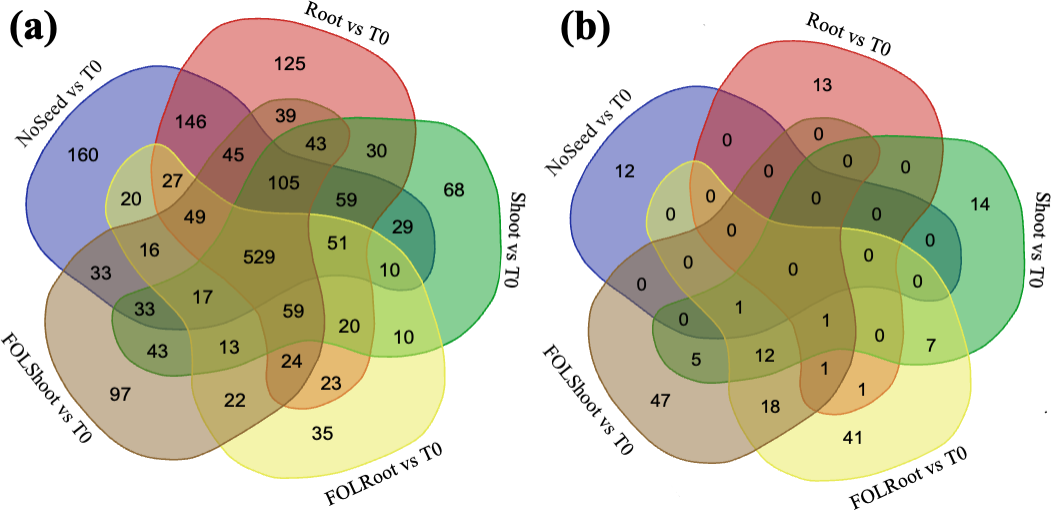
(**a**) Venn diagram of genes with significant positive fitness with respect to the wild type for each condition versus T0. (**b**) Venn diagram of genes with significant negative fitness with respect to the wild type for each condition versus T0.

Comparison of fitness values [log_2_FoldChange(FC) relative to T0] identified 529 genes consistently contributing to growth across all conditions (Table S1). These were enriched in amino acid biosynthesis, DNA replication, oxidoreductase activity, and metal binding. The genes *waaC* (MMB18_RS04835) and *gspD* (MMB18_RS00365), which encode a lipopolysaccharide heptosyltransferase I and a type II secretion system secretin, respectively, were common among the 529 fit genes across all comparisons (Fig. 6). In contrast, subsets of genes were condition-specific. For instance, 160 genes showed positive fitness exclusively under the No Seed condition. These include genes that encode proteins involved in amino acid biosynthesis and purine metabolism. One hundred twenty-five genes showed positive fitness exclusively under the Root condition. These include genes involved in DNA replication and purine biosynthesis. Thirty-five genes showed positive fitness exclusively under the FOLRoot condition. These genes mostly encoded for proteins involved in branched-chain amino acid biosynthesis, DNA replication, and valine, leucine, and isoleucine biosynthesis. Ninety-seven genes showed positive fitness exclusively under the FOLShoot condition. These genes were mostly involved with DNA replication, damage, and repair. Common genes across treatments included those encoding the cellulose synthase complex periplasmic endoglucanase BcsZ (in NoSeed, Root, Shoot, and FOLShoot compared to T0), *cobW*, a protein involved in Vitamin B12 biosynthesis (in Shoots and FOLShoot compared to T0), (in Shoots and FOLShoot compared to T0) and *hpnA*, a protein component of hopanoid synthesis (in NoSeed and FOLShoot compared to T0). Similar to work on *B. vietnamiensis*-rice interactions (12), an enhanced fitness for queuosine synthesis (*queG* in NoSeed, Root, and FOLRoot compared to T0) was observed. On the other hand, for genes that showed negative fitness in the wild type, no genes were shared across all conditions, and nearly no genes overlapped among the NoSeed, Root, and Shoot conditions, while some overlap was observed for FOLRoot and FOLShoot conditions (Fig. 6). Twelve genes were common to FOLRoot, FOLShoot, and Shoot when compared to T0. The genes with negative fitness in the FOLRoot, FOLShoot, and Shoot comparisons based on GO analysis were processes associated with siderophore transport, siderophore uptake, transmembrane transporter activity, and TonB-dependent receptors. These results suggest that siderophores, which typically sequester iron and are used by organisms as a defense mechanism, are not found here to confer fitness to *B. contaminans* in the presence of FOL.

### Nutrient limitation primes secondary metabolite production

**Fig 7:**
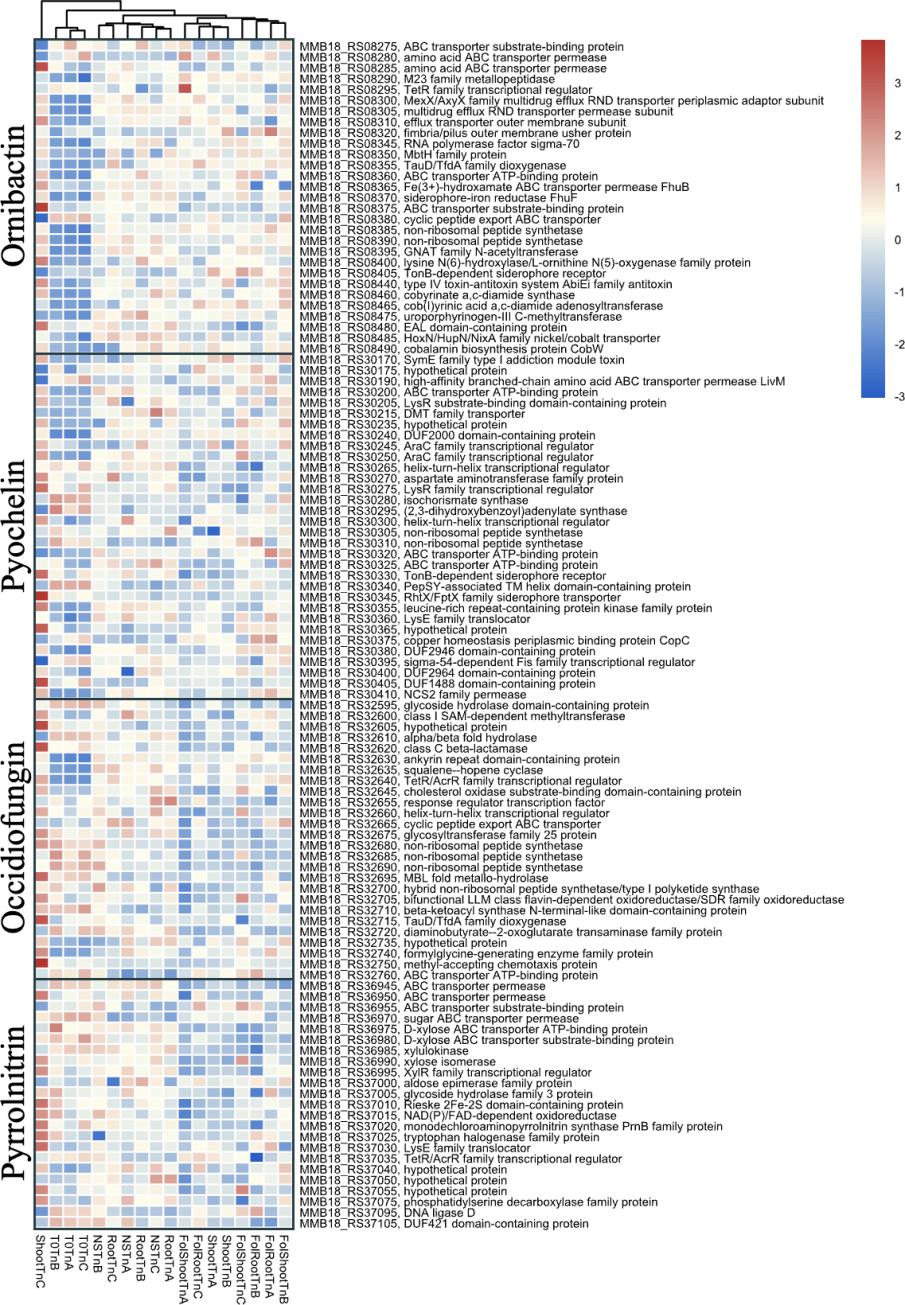
Hierarchical clustering of the gene counts (translated to wild-type representation) for secondary metabolites gene clusters of *B. contaminans* according to condition.

Comparisons of tissue culture vs T0 highlighted increased fitness contributions of genes involved in the biosynthesis of the secondary metabolites, ornibactin and pyochelin (Fig. 7; Table S2). This suggests that the low nutrient conditions of the tissue culture environment, as compared to the rich media environment of T0, may lend to the production of the iron-scavenging compounds, ornibactin and pyochelin. The antifungal and cytotoxic compounds of occidiofungin and pyrrolnitrin have a baseline fitness that does not change from T0 to tissue culture conditions, and perhaps counterintuitively, the antifungal compounds appear to again reduce in fitness with the introduction of FOL. Furthermore, FOL does not appear to enhance the fitness of iron-scavenging siderophores (Fig. 7). Similar results for the baseline production of occidiofungin and ornibactin in the root zone of citrus were observed for *Burkholderia* strain B23 (15). This lack of increased antifungal production by *B. contaminans* in the presence of FOL indicates that there may be an advantage for the microbe to maintain the fungi.

### Type II secretion system supports root and shoot colonization

**Fig 8:**
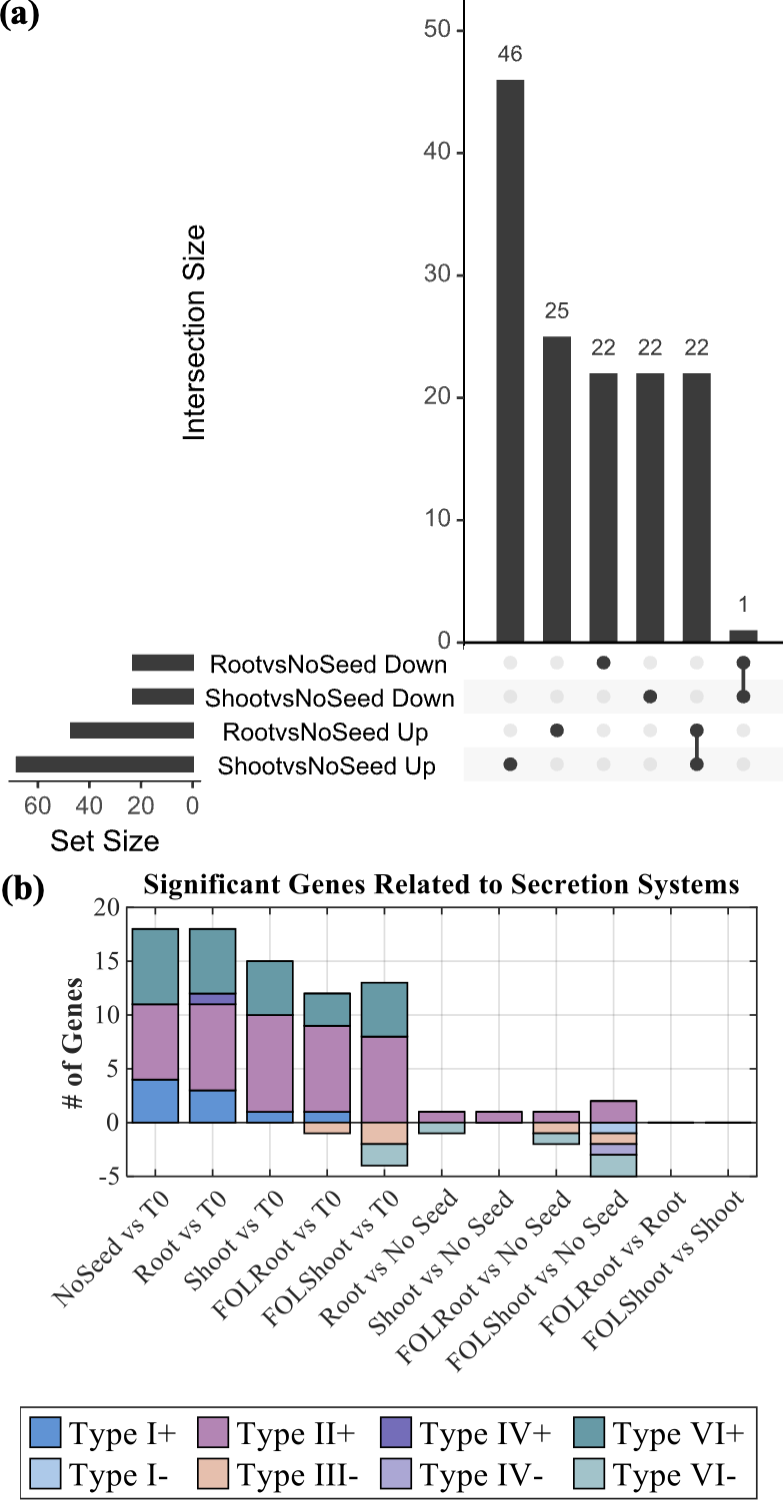
(**a**) UpSet plot illustrating the overlap of significantly fit (up) and significantly unfit (down) genes with respect to the wild type for the Root and Shoots of the Tn-Seq treated plant to the NoSeed control. (**b**) Counts of significantly fit and unfit secretion systems with respect to the wild type found per condition, as compared to T0 and the NoSeed control.

Using the NoSeed condition as baseline, we identified genes specifically required for root and shoot colonization (Fig. 8; Table S3). Among these, a type II secretion system (T2SS) gene (secretion protein N) emerged as central to fitness in the root and shoot, as well as *RpoE* for the root that encodes an RNA polymerase sigma factor. RpoE is likely involved in stress associated with colonizing the plant root (18).

Counts of the secretion system genes with greater than fourfold positive and negative fitness values for all treatments as compared to T0 or NoSeed controls were made (Fig. 8). Where type I, II, and VI secretion systems (T1SS, T2SS, T6SS) were broadly important in comparisons to nutrient-rich T0, T2SS remained consistently advantageous in low-nutrient plant-associated contexts, while T6SS activity correlated with decreased fitness. This T2SS fitness does not further increase when the plant is challenged with FOL, suggesting a baseline need for T2SS activity both with and without the presence of FOL. The trend for T6SS to be less fit and T2SS to contribute to fitness was similarly observed in a prior study that investigated a transposon library of *B. vietnamiensis* as it colonized the rice root zone (12).

### Nudix hydrolase underpins fungal interaction in the root zone

**Fig 9:**
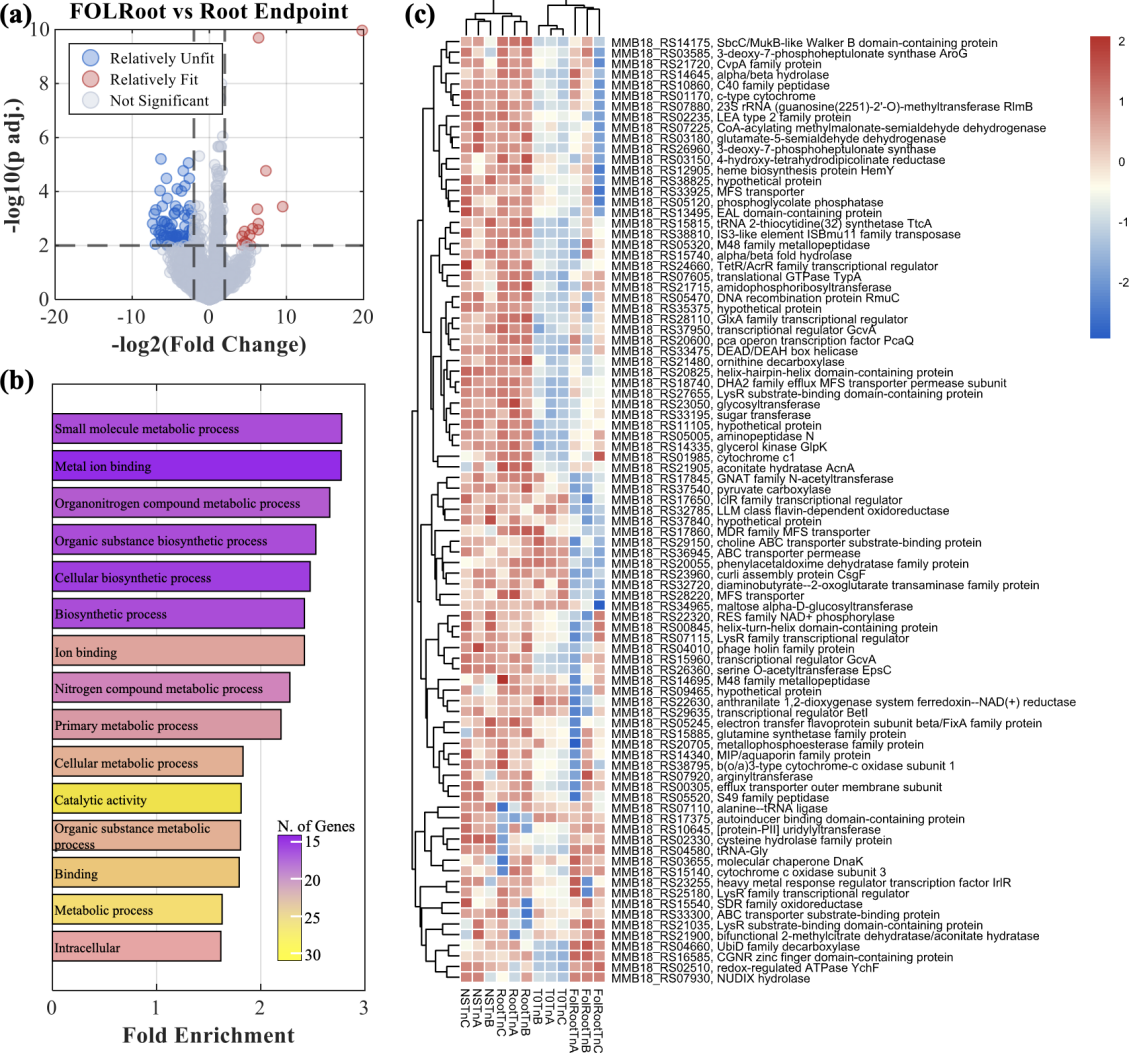
(**a**) Volcano plot depicted the 89 significant genes and their fitness with respect to the wild type for the comparison of FOLRoot vs Root. (**b**) The GO terms enriched among these 89 genes. (**c**) Hierarchical clustering of the counts for the 89 significant genes (translated to wild-type representation) from the FOLRoot vs Root comparison with the corresponding translated counts of the 89 in the T0 and NoSeed conditions.

Direct comparison of FOL-infected roots to uninfected roots identified 89 genes with significant fitness shifts: 15 positive and 74 negative (Fig. 9; Table S4). Among the most significant was a Nudix hydrolase (MMB18_RS07930), whose function likely involves reducing host- and fungus-derived reactive oxygen species (ROS) through ion-dependent hydrolysis. Hierarchical clustering showed FOLRoot replicates grouped with nutrient-rich T0 samples, while uninfected Root samples clustered with NoSeed controls, suggesting that fungal presence alters nutrient availability for *B. contaminans*. Furthermore, the genes that have positive fitness in the Root and NoSeed treatments but negative fitness under the nutrient-rich T0 and FOL challenge are involved in nitrogen metabolic processes. Together, these data support a model where Nudix hydrolase activity facilitates *B. contaminans* persistence by mitigating ROS stress and exploiting nutrients liberated during fungal challenge, and is consistent with proteomics observations in *Burkholderia glathei* co-cultured with *Alternaria* and *Fusarium* (18).

## DISCUSSION

An isolate of *B. contaminans* from the ISS Veggie facility was investigated for its PGP and antifungal potential due to evidence from earlier soil-based studies where tomato plants exhibited a measurable growth advantage when inoculated with *B. contaminans* strain AY001, in the presence of FOL (16). Given that spaceflight represents a closed ecosystem where microbial proliferation can have both beneficial and hazardous outcomes, understanding the mechanisms underlying bacterial-plant-fungal interactions is essential. Microbial tracking using fluorescently tagged strains allows spatial and temporal monitoring of bacterial colonization, while transposon sequencing enables the dissection of gene-level contributions to fitness during such interactions. Together, these tools provide a baseline understanding of microbial behavior in terrestrial settings before strains are evaluated under the altered physical constraints of spaceflight.

*Burkholderia* species are highly adaptable and capable of colonizing multiple plant niches, including the rhizoplane, rhizosphere, endosphere, and, in some cases, translocating from the rhizosphere to the phyllosphere (18). Previous phylogenetic studies have shown no direct correlation between genetic relatedness and plant niche preference, suggesting that colonization traits are broadly distributed across the *Burkholderia* genus (19). Studies employing GFP tagging (20), plating (21), and microscopy (22) have demonstrated that *Burkholderia* typically colonize roots preferentially before shoots, with bacterial populations often enriched at the root surface and intercellular spaces. In this study, however, *B. contaminans* was recovered at similar magnitudes from both roots and shoots of 28-day-old tomato plants. Fluorescent microscopy confirmed strong colonization at the root-shoot junction in the rhizoplane, consistent with prior observations in grapevine (*Burkholderia phytofirmans* sp. PsJN) (23) and rice (*B. cepacia* strains RRE-3 and RRE-5) (24), where bacteria preferentially accumulated at the base of lateral roots and junction sites. These findings support the view that *B. contaminans* can establish robustly across multiple tomato plant compartments under tissue culture conditions.

Our Tn-Seq analysis provided further insight into the metabolic strategies employed by *B. contaminans*. Genes encoding proteins involved in secondary metabolite biosynthesis, including the siderophores pyochelin and ornibactin, were induced in the low-nutrient tissue culture environment compared to the nutrient-rich input library. By contrast, genes involved in producing the antifungals occidiofungin and pyrrolnitrin did not increase beyond baseline. Interestingly, the presence of FOL did not enhance the relative fitness of genes involved in these metabolite pathways. This phenomenon has been observed for *Pseudomonas aeruginosa* when siderophore mutants for pyoverdin and pyochelin were applied to tomato for biocontrol of *Pythium*. The conclusion was that mutants retain function due to the redundancy provided by other siderophores (25). An alternative explanation for the lack of FOL elimination by *B. contaminans* is the need to acquire nutrients in the low-nutrient tissue culture setting. Bacterial-fungal interactions (BFI) are known to provide the bacteria with carbon, nitrogen, and phosphorus, whereas the bacteria provide protective molecules, such as Vitamin B12, antibiotics, and toxin resistance molecules (26). These results suggest that in this environment, *B. contaminans* does not function solely as an antifungal competitor but may instead exploit the fungal pathogen as a nutrient source. Such metabolic flexibility may underpin the persistence of *B. contaminans* in competitive root environments.

Perhaps the most striking result obtained was the identification of a Nudix hydrolase gene with an exceptionally high fitness requirement (−1 × log₂FC ≈ −19) in the tomato root zone challenged with FOL. *Burkholderia* genomes typically encode ~15 Nudix hydrolases, but the strong signal from this specific gene suggests a specialized role. In *Escherichia coli*, the well-characterized Nudix hydrolase MutT detoxifies oxidized guanine nucleotides (8-oxo-dGTP), preventing mutagenesis; loss of MutT results in a ~1,000-fold increase in spontaneous mutation rates (27). While proteomic studies in *Burkholderia glathei* showed reduced MutT abundance during fungal co-culture (28), our findings indicate that in the tripartite tomato–*B*. *contaminans*–FOL interaction, this Nudix hydrolase becomes nearly essential. Analogous to the Nudix motif-containing effector Avr3b in *Phytophthora sojae*, which suppresses host ROS bursts to evade effector-triggered immunity (29), the *B. contaminans* Nudix hydrolase may mitigate intracellular or extracellular stresses triggered by fungal metabolites or plant defense responses. This reliance on a Nudix hydrolase highlights the importance of stress detoxification pathways in sustaining bacterial survival within a plant-fungal-bacterial tripartite system. A recent review has highlighted the heightened ROS response of plants grown in the spaceflight environment, pointing out that ROS secretion from the extracellular space outside of the plant cell membrane, known as the apoplast, can further shape microbial interactions (30). Taken together, our findings suggest that while this ISS *B. contaminans* isolate does not consistently confer plant growth promotion in the tissue culture environment, it maintains persistence in the tomato root zone under fungal challenge through stress-adaptation mechanisms rather than direct antifungal antagonism.

As a note of caution—though not observed in this study—BFI interactions can include compounding scenarios where bacteria directly attach to, or even invade, their fungal partners. For example, *Burkholderia glumae* attachment to *Fusarium graminearum* enhances sporulation *in vitro* (31), and a co-infection of rice increases both disease severity and the production of the mycotoxin deoxynivalenol (32). Members of the BCC, including *B. contaminans*, are classified as Biosafety Level 2 and should always be handled with appropriate precautions. To date, BCC strains are primarily associated with hospital-acquired infections and are not associated with food-borne illness, like the related *B. gladioli* pathovar *cocovenenans* (33). The closest account of an orally acquired BCC infection involved a hospital outbreak due to the use of contaminated mouthwash (34). During transmission, it is the T2SS and T6SS that facilitate BCC colonization of plant and fungal hosts than can enable entry and survival in human immune cells (35, 36). Our prior work has demonstrated that simulated microgravity and nutrient limitation stimulate T2SS activity, toxin production, and non-ribosomal peptide synthesis for an ISS water processor assembly isolate of *B. contaminans* (37). These phenotypes may be transient, given our other prior work showed that *B. contaminans* isolates from the ISS PWD were no more cytotoxic to macrophage than terrestrial strains (2), but nonetheless demonstrated the capacity to lyse murine macrophages. Together, our findings highlight the dual potential of *Burkholderia*: as resilient colonizers capable of stabilizing plant microbiomes, yet also as opportunistic pathogens that warrant careful monitoring. Future work should define the specific substrates of the Nudix hydrolase and clarify its role in modulating plant-microbe-fungal interactions under the unique constraints of spaceflight agriculture and do so preemptively in an uncrewed setting.

## MATERIALS AND METHODS

### *B. contaminans* reporter strain construction

An electrocompetent culture of the *B. contaminans* isolate in this study was prepared following the small batch protocol from Barrick et al. Culturing steps used tryptic soy agar (TSA) or tryptic soy broth (TSB), and five total wash cycles with ice-cold 10% glycerol for the generation of salt-free electrocompetent cells. For electroporation, 1.5 µL of the plasmid pCAT220 at 100 ng/µL was added to 30 µL of electrocompetent *B. contaminans* cells on ice and incubated for 10 min. The mixture was applied to a chilled 1 mm gap electroporation cuvette, then pulsed with standard settings for *E. coli*. The electroporated cells were promptly recovered in 500 µL of Super Optimal broth with Catabolite repression (SOC) media and incubated for 1 h at 35°C at 200 rpm. The cells were plated on TSA plus 50 μg/mL kanamycin and incubated overnight at 37°C to select for the transformed cells containing the pCAT220 plasmid.

### *B. contaminans* reporter strain application, plant growth, imaging, and enumeration

Seeds of tomato (*Solanum lycopersicum*) cultivar Red Robin were sanitized using a chlorine gas method (1) and then coated with a pullulan seed coat harboring the fluorescently labeled bacterial strain *B. contaminans* with known starting concentrations of 10^8^ CFU/mL achieved by encapsulating the seeds in 10 µL droplets of the bacterial cultures at 10^10^ CFU/mL. The seeds were allowed to dry before cutting coupons of seed-embedded film for planting. Coupons containing the inoculated seeds were placed on 645 cm^2^ square petri dishes with optically clear plant growth media (Murashige & Skoog modified basal medium with Gamborg vitamins, Cat. No. M404; PhytoTech Labs, Lenexa, KS). Each replicate of this experiment consisted of two negative control Petri plates, which had sterile seeds placed on the media surface, two seed film control Petri plates, which had seed film strips containing sterile seeds encapsulated with sterile DI water, and two bacterial test plates. The plates were placed horizontally to fully dissolve the seed film and allow for the initial establishment of the plants. Following root emergence in the majority of the seeds, the petri dishes were reoriented vertically. The plants grew for 28 days in a controlled environment chamber set to 50% relative humidity (RH) and ambient CO_2_. The plates were observed with a Nikon SMZ25 stereo microscope equipped with brightfield and fluorescence optics. Images were acquired bi-weekly to track the movement of the microbes during plant growth. GFP fluorescence was quantified from the captured grayscale images from the green channel using ImageJ (ImageJ v1.49 software). The area, average mean intensity, and integrated density of the root perimeter and at least three background regions were measured from the green channel. Average corrected fluorescence was defined as the background-subtracted mean intensity of the root region, normalized to its measured area.

### FOL zone of inhibition evaluation

Zone of inhibition testing on PDA was conducted for *B. contaminans*, *P. fulva,* and *P. agglomerans* to assess clearance of FOL following the protocol described in reference 2.

### Tripartite FOL challenge experiment

Growth vessel assemblies were created using autoclaved magenta vessels joined via custom 3D-printed connectors that were sterilized with ethylene oxide and thoroughly off-gassed. Sterilized vessels were filled with 250 mL of the same optically clear plant growth media utilized in the fluorescence tracking work. Once cooled and solidified, coupons of seed film containing two seeds were placed in one corner of each vessel with three vessels for each of the following treatments: (i) Seed film control (no *Burkholderia* or FOL), (ii) FOL control (only “FOL”), (iii) *B. contaminans* control (only *Burkholderia,* “BC”), (iv) an experimental treatment of *B. contaminans* with FOL (“BC+FOL”), and (v) a negative control (NC) having only the sanitized tomato seeds used in this experiment which did not utilize seed film (Table 2). Magenta vessels were placed into a controlled-environment chamber set to 50% RH and ambient CO_2_. At 7 days after initiation, vessels were thinned to one plant per vessel, and 10 µL of 10^3^ CFU/mL FOL (isolate GEV1400 [38]) culture was applied to the corner opposite to the established plant in the fungal control and experimental treatment vessels. Plants were grown for 28 days total before they were harvested. At harvest, shoot and root tissues were separated and placed into 50 mL Falcon tubes with sterile 3 mm glass beads and 20 mL of buffered peptone water. Samples were shaken using a Bead Ruptor (Omni International, Kennesaw, GA, USA) for 90 s with 30 s rest periods following every 30 s shaking interval. Shaken samples were plated on TSA plates containing 50 µg/mL of kanamycin (which selects for the *B. contaminans* strain harboring the pCAT220 plasmid) to determine the quantity and general location of *B. contaminans* pCAT220 cells within the plants. Plates were incubated at 37°C for 48 h, at which point they were removed, and fluorescent colonies were enumerated.

**TABLE 2:** Number of samples for statistical measurements in the FOL challenge experiment

FOL study	Only seeds (negative control)	Seed plus seed film alone	Seed plus seed film plus FOL	Seed plus seed film plus *B. contaminans*	Seed plus seed film plus *B. contaminans* plus FOL
No. of samples	9 (3 biological replicates × 3 growouts)	9 (3 biological replicates × 3 growouts)	9 (3 biological replicates × 3 growouts)	9 (3 biological replicates × 3 growouts)	9 (3 biological replicates × 3 growouts)

### Plasmid donor strain construction

An electrocompetent culture of the *E. coli* diaminopimelic acid auxotrophic strain, RH03, was prepared following the small batch protocol (39). Briefly, the freezer stock of RH03 was revived on TSA plus 400 μg/mL diaminopimelic acid, a single colony was used to prepare an overnight culture in TSB plus 400 μg/mL diaminopimelic acid, was diluted 1:100 in 10 mL of fresh TSB plus 400 μg/mL diaminopimelic acid and allowed to grow to exponential phase (OD_600_ of 0.05) before harvesting by centrifugation at 4°C. To prepare electrocompetent cells, the media was decanted from the harvested pellet, ice-cold 10% glycerol was used to resuspend the pellet before centrifugation (5 min at 6,000 rpm) and decanting for four more cycles of washing to remove any salts and generate 30 µL aliquots of electrocompetent RH03 cells. For electroporation, 1.5 µL of the plasmid pBAM1-GFP at 100 ng/μL was added to 30 µL of electrocompetent RH03 cells on ice and incubated for 10 min. The mixture was applied to a chilled 1 mm gap electroporation cuvette, then pulsed with standard settings for *E. coli*. The electroporated cells were promptly recovered in 500 µL of SOC media and incubated for 1 h at 37°C at 200 rpm. The cells were plated on TSA plus 400 μg/mL diaminopimelic acid plus 50 μg/mL kanamycin and incubated overnight at 37°C to additionally select for the transformed cells containing the pBAM1-GFP plasmid.

### Tn-Seq library construction

#### Conjugation of Tn5 plasmid from the donor to *B. contaminans*

To generate the transposon library for an isolate of *B. contaminans* obtained from the Veggie facility on the ISS, the general conjugation protocol from Barrick et al. 2025 (40) was followed. Briefly, a −80°C freezer stock of the recipient *B. contaminans* strain was revived on a TSA Petri plate and grown for 2 days at 35°C. One colony from this plate was selected and grown overnight in TSB at 35°C with shaking at 200 rpm. Concurrently, the −80°C stock of the donor RH03 strain harboring the pBAM1-GFP plasmid was revived on TSA plus 400 μg/mL diaminopimelic acid plus 50 μg/mL kanamycin, and one colony was selected and grown overnight in TSB plus 400 μg/mL diaminopimelic acid plus 50 μg/mL kanamycin at 35°C with shaking at 200 rpm. One milliliter of each culture was spun down at 3,000 rpm for 5 min and washed with phosphate-buffered saline (PBS) and resuspended in 500 μL PBS. A 1:2 ratio of donor to recipient was mixed and allowed to conjugate by plating three separate 100 μL spots of the mixture on a TSA plus 400 μg/mL diaminopimelic acid plate overnight at 35°C. The next day, the conjugation mixtures were scraped from the agar plate, and each was resuspended in 1 mL of PBS for a total of 3 mL. The mixture was divided into 100 μL aliquots and spread among 30, 120 × 120 mm square Petri dishes filled with TSA with 50 μg/mL kanamycin to obtain evenly spaced transposed mutants without the auxotrophic donor. The plates were incubated for 48 h at 35°C, and the next day, all colonies were scraped and combined to generate the *B. contaminans* Tn-Seq library. Presence of the transposon was confirmed in the library by DNA extracting an aliquot of the library, followed by PCR with the following primers: transposoncheck_FWD: TGCCCGAAGGTTATGTACAGG and transposoncheck_REV: TTCATCCGCCTGATGCAC. The library was then frozen with a final concentration of 15% glycerol at −80°C.

#### Tripartite Tn-Seq plant growth experiment

Growth vessels were prepared in the same way as above. In each vessel, two seed films were placed in one corner of each for the following scenarios: (i) “NoSeed”—a control where only the *B. contaminans* Tn-Seq library (10^8^ CFU/mL) is applied, (ii) “Root” or “Shoot”—a treatment where *B. contaminans* Tn-Seq library (10^8^ CFU/mL) plus seed is applied, and (iii) “FOLRoot” or “FOLShoot”—a treatment of *B. contaminans* Tn-Seq library (10^8^ CFU/mL) plus seed for additional FOL challenge. The final 10^8^ CFU/mL count was obtained by adding 10 µL droplets of the bacterial cultures at 10^10^ CFU/mL to the seed film. The *B. contaminans* Tn-Seq library was revived by slightly thawing the freezer stock and pipetting 100 μL of the Tn-Seq library into 3 mL of TSB containing 50 μg/mL kanamycin and incubating at 35°C with shaking at 200 rpm for 16 h. Three separate Tn-Seq starter cultures were prepared to serve as time point 0 (T0) and to inoculate the seed films as the three biological replicates (TnA, TnB, and TnC) of the experiment. Cells that were not used for the experiment were retained as T0 for Tn-Seq sequencing. Magenta vessels were placed into a controlled-environment chamber set to 50% RH and ambient CO_2_. At 7 days after initiation, vessels were thinned to one plant per vessel, and 10 µL of 10^3^ CFU/mL FOL culture was applied to the corner opposite to the established plant in the fungal control and experimental treatment vessels. Plants were grown for 28 days total before they were photographed and harvested. At harvest, shoot and root tissues were separated and placed into 50 mL Falcon tubes with 10 mL of 50 μg/mL kanamycin in TSB media and allowed to grow for 16 h at 35°C with shaking at 200 rpm to select for the transposed *B. contaminans* Tn-Seq library. Plant material was removed from the Falcon tube, the bacterial culture was pelleted by centrifugation at 3,000 rpm for 5 min, and the media was decanted. Three cell pellets were obtained for scenarios a, b roots, b shoots, c roots, c shoots, and time point 0 for a total of 18 cell pellets for DNA extraction. The nutrient profiles of TSB and optically clear plant growth media are presented in Table 3. The presence of the pullulan seed film could also serve as a carbon source in the tissue culture environment.

**TABLE 3:** Nutrient component class break out for TSB and Murashige & Skoog modified basal medium (MS) with Gamborg’s vitamins

Component class	TSB (tryptic soy broth)	MS + Gamborg’s (no sucrose)
Carbon/energy source	Glucose + abundant peptides	None
Nitrogen source	Peptides, amino acids	Only inorganic N (NH_4_^+^, NO_2_^−^)
Growth factors	Complex digests provide nucleotides, fatty acids, cofactors	Only vitamins (thiamine, pyridoxine, nicotinic acid, glycine)
Osmotic balance and salts	Moderate NaCl (~0.5%)	High salt/osmolarity (esp. nitrates)
pH	~7.3	~5.7
Growth outcome	Robust growth, high density	Negligible/poor growth
Nature of medium	Complex, nutrient-rich	Defined, plant-optimized

#### Tn-Seq experiment sequencing library preparation and sequencing

Nineteen cell pellets of DNA were extracted using the DNeasy PowerLyzer PowerSoil Kit (Qiagen, Germany). The samples extracted are outlined in Table 4.

**TABLE 4:** Number of samples per condition in the Tn-Seq study

Transposon sequencing	Input library “Input”	T0 library “T0”	Seed film plus Tn-Seq library without seed “No Seed”	Shoots from Seed film plus Tn-Seq library plus seed “Shoot”	Roots from Seed film plus Tn-Seq library plus seed “Root”	Shoots from Seed film plus Tn-Seq library plus seed plus FOL “FOLShoot”	Roots from Seed film plus Tn-Seq library plus seed plus FOL “FOLRoot”
No. of samples	1	3	3	3	3	3	3

Sequencing library preparation of samples for the Tn-Seq experiment followed the protocol used in Ganesan et al. 2021 (15) with modifications. The NEBNext Ultra II DNA Library Prep Kit for Illumina was used, but at half reaction volumes. Specifically, 400 ng of input DNA in 13 μL was processed for each of the 19 samples. To the DNA for each sample, 3.5 μL NEBNext Ultra II FS Reaction Buffer and 1 μL of NEBNext Ultra II FS Enzyme Mix were added. This mixture was incubated for 15 min at 37°C and then 30 min at 65°C to fragment the DNA and terminate the fragmentase activity. Next, 15 μL of NEBNext Ultra II Ligation Master Mix, 0.5 μL of NEBNext Ligation Enhancer, and 1.25 μL of NEBNext Adaptor for Illumina were added to this mixture and incubated for 15 min at 20°C. A total of 1.5 μL USER Enzyme was added to this mixture and incubated at 37°C for 15 min with a heated lid set to ≥47°C. This 37.75 μL reaction volume was brought to 100 μL using 0.1× TE and size selected with SPRI Ampure magnetic beads with right side selection of 0.3× and a left side selection of 0.15×. The size-selected fragments concentrated into 7.5 μL were PCR amplified using 2.5 μL of a forward primer specific for the transposon that also harbored a 5′ biotin label, 2.5 μL of a P7 barcoded sequencing primer NEBNext Multiplex Oligos for Illumina (Index Primers Set 1 and 2), and 12.5 μL of NEBNext Ultra II Q5 Master Mix. The thermocycler denature, anneal, and extension times were an initial 98°C for 30 s, then 10 rounds of 98°C for 10 s, 65°C for 30 s, and 72°C for 30 s, with a final extension of 72°C for 2 min. This reaction was cleaned up by 0.9× left side selection with SPRI Ampure magnetic beads. A volume of 12.5 μL of the biotin-tagged product was hybridized to streptavidin magnetic beads to select for reads harboring the transposon primer. The beads were washed with 1× binding and wash buffer to remove any products not harboring the biotinylated transposon-specific DNA fragments. A second PCR was carried out using the hybridized biotinylated transposon-streptavidin beads in 7.5 μL of 0.1× TE, 2.5 μL of a forward primer that included the P5 adapter sequence, followed by a random hexamer sequence and the transposon-specific primer sequence, 2.5 μL of the corresponding P7 barcoded sequencing primer NEBNext Multiplex Oligos for Illumina (Index Primers Set 1 and 2) used in the first PCR reaction for the respective sample, and 12.5 μL of NEBNext Ultra II Q5 Master Mix. Each library was quantified using the Qubit DNA High Sensitivity (HS) kit and the Agilent Bioanalyzer DNA HS kit. The 19 barcode sequencing reads were pooled to achieve a 20 nM sample in 40 μL and shipped to Azenta GeneWiz for sequencing on the Illumina Nextseq platform.

#### Tn-Seq analysis

For Tn-Seq analysis, read 1 (P5) raw sequencing reads for all libraries were trimmed for the following transposon-specific sequence: GGTGCATCAGGCGGATGAACGCGCGGCCGC using Cutadapt (version 5.1). Any reads that did not contain the sequence were discarded. Surviving reads were mapped to the reference genome (*B. contaminans*, strain B17-01563-1, GCF_022533485.1_ASM2253348v1_genomic.fna) using bowtie2 (version 2.5.4). The “local” option was used, which allows bowtie to trim reads according to any detected trends in the 5′ region of the read for better alignment. This process allows for any Tn5 mosaic ends that were not trimmed by Cutadapt to be removed during the alignment step. The featureCounts tool (Subreads package, version 2.1.1) was used to compile all mapped read counts by gene feature. The resulting counts table was processed to keep genes with nonzero counts for all the triplicates in the T0 control. The counts table was fed into the DESeq2 program in RStudio (RStudio version 2024.09.1+394) to arrive at log_2_FoldChange values with associated adjusted *P* values across the mutated genes for the relevant comparisons between treatment groups. Outputs from DESeq2 for each of the mutated genes were multiplied by −1 to obtain a relative gene fitness measurement for the wild type of *B. contaminans*. GO term enrichment was determined using ShinyGO 0.8. Differences and commonalities in relative gene fitness among the experimental treatment groups were visualized using UpSet and volcano plots. The antiSMASH tool was used to identify secondary metabolites from the *B. contaminans* reference genome. The genes with the most significant fitness contributions and from related operons were visualized using the “pheatmap” tool in RStudio. Samtools was used to convert the sam file output from bowtie2 to a bam file; the bam file was sorted, and Samtools’ “coverage” feature was used to find a corresponding coverage for each Tn-Seq library and the initial input Tn-Seq mutant pool across the four chromosomes of the reference genome. The TPP tool of the TRANSIT program (41) was used to establish Tn5 insertion counts for each library.

## Data Availability

Data are available in the NCBI Gene Expression Omnibus repository under accession no. GSE315141.

## SUPPLEMENTAL MATERIAL

Supplemental material aem.01941-25-s0001.pdf Tables S1 to S4.

Graphical abstract aem.01941-25-s0002.tiff Study overview.

ASM does not own the copyrights to Supplemental Material that may be linked to, or accessed through, an article. The authors have granted ASM a non-exclusive, world-wide license to publish the Supplemental Material files. Please contact the corresponding author directly for reuse.
