# Supplementary material for: Transposon sequencing reveals Burkholderia gene fitness in a spaceflight-relevant plant-pathogen interaction
Source: Appl Environ Microbiol. 2026 Jan 13;92(2):e01941-25. doi: 10.1128/aem.01941-25 (PMC12915293; doi:10.1128/aem.01941-25)
Supplement: Supplemental material — Tables S1 to S4. [file aem.01941-25-s0001.pdf]

Supplemental Table 1: The 529 B. contaminans genes that had fitness values greater than 2 (|log2FC\>2) and an adjusted p-value less than 0.01 for all groups when compared to the T0 control.

Gene ID	Gene Name	Gene Product	Gene Ontology	Comparison	log2FC	padj
MMB18_RS00020	NA	hypothetical protein	NA	No Seed vs T0	-6.25	3.22E-05
				Root vs T0	-9.08	3.07E-09
				Shoot vs T0	-5.93	1.38E-04
				FolRoot vs T0	-8.29	9.13E-08
				FolShoot vs T0	-7.54	7.91E-07
MMB18_RS00025	drmB	DUF1998 domain-containing protein	NA	No Seed vs T0	-4.81	8.12E-04
				Root vs T0	-8.68	8.69E-09
				Shoot vs T0	-10.2	2.32E-09
				FolRoot vs T0	-7.61	4.55E-07
				FolShoot vs T0	-6.04	3.35E-05
MMB18_RS00040	NA	hypothetical protein	NA	No Seed vs T0	-5.47	5.45E-21
				Root vs T0	-9.27	1.03E-11
				Shoot vs T0	-8.57	1.41E-13
				FolRoot vs T0	-6.93	1.34E-14
				FolShoot vs T0	-8.12	2.66E-13
MMB18_RS00045	NA	helicase-related protein	NA	No Seed vs T0	-4.05	4.56E-04
				Root vs T0	-5.12	8.13E-06
				Shoot vs T0	-6.2	2.07E-07
				FolRoot vs T0	-3.52	3.22E-03
				FolShoot vs T0	-7.8	4.01E-11
MMB18_RS00050	NA	ATP-binding domain-containing protein	NA	No Seed vs T0	-7.27	1.19E-06
				Root vs T0	-5.57	2.01E-04
				Shoot vs T0	-7.63	1.71E-06
				FolRoot vs T0	-6.95	5.99E-06
				FolShoot vs T0	-7.37	1.27E-06
MMB18_RS00100	NA	cytochrome b	GO:0022904, GO:0009055, GO:0020037, GO:0046872, GO:0016020	No Seed vs T0	-5.88	2.01E-08
				Root vs T0	-7.62	2.17E-07
				Shoot vs T0	-6.29	4.92E-08
				FolRoot vs T0	-6.34	1.83E-06
				FolShoot vs T0	-8.06	4.03E-08
MMB18_RS00120	NA	hypothetical protein	NA	No Seed vs T0	-7.51	3.48E-09
				Root vs T0	-7.33	5.25E-09
				Shoot vs T0	-4.61	3.38E-09
				FolRoot vs T0	-6.33	8.68E-09
				FolShoot vs T0	-7.78	7.84E-09
MMB18_RS00155	NA	hypothetical protein	NA	No Seed vs T0	-7.73	2.35E-09
				Root vs T0	-6.99	2.95E-10
				Shoot vs T0	-3.76	9.59E-07
				FolRoot vs T0	-6.22	2.63E-09
				FolShoot vs T0	-4.35	1.88E-08
MMB18_RS00165	NA	DUF2628 domain-containing protein	NA	No Seed vs T0	-4.09	1.76E-03
				Root vs T0	-4.35	1.08E-03
				Shoot vs T0	-4.76	9.50E-04
				FolRoot vs T0	-5.09	6.87E-04
				FolShoot vs T0	-5.67	2.11E-04
MMB18_RS00195	NA	glycine zipper 2TM domain protein	NA	No Seed vs T0	-5.49	5.91E-05
				Root vs T0	-6.28	6.94E-05
				Shoot vs T0	-7.17	1.70E-05
				FolRoot vs T0	-6.66	1.29E-04
				FolShoot vs T0	-6.58	5.15E-05
MMB18_RS00220	NA	sensor histidine kinase	GO:0007165, GO:0000155, GO:0004673, GO:0005524	No Seed vs T0	-2.52	5.46E-04
				Root vs T0	-3.82	3.47E-07
				Shoot vs T0	-3.02	1.42E-04
				FolRoot vs T0	-2.8	2.37E-04
				FolShoot vs T0	-2.46	1.06E-03
MMB18_RS00285	NA	LysR family transcriptional regulator	NA	No Seed vs T0	-5.27	2.37E-07
				Root vs T0	-5.11	5.19E-07
				Shoot vs T0	-4.15	1.07E-04
				FolRoot vs T0	-4.17	5.71E-05
				FolShoot vs T0	-5.36	4.84E-07
MMB18_RS00320	gspL	type II secretion system protein GspL	GO:0015628, GO:0009276, GO:0015627	No Seed vs T0	-3.74	2.56E-12
				Root vs T0	-4.04	6.83E-14
				Shoot vs T0	-5.04	2.08E-16
				FolRoot vs T0	-4.86	1.30E-16
				FolShoot vs T0	-4.45	8.76E-15
MMB18_RS00350	NA	type II secretion system protein N	NA	No Seed vs T0	-3.45	4.92E-08

				Root vs T0	-4.36	1.74E-10
				Shoot vs T0	-3.81	7.77E-08
				FolRoot vs T0	-4.58	1.19E-09
				FolShoot vs T0	-4.32	2.21E-09
MMB18_RS00365	gspD	type II secretion system secretin GspD	GO:0015628, GO:0015627, GO:0019867	No Seed vs T0	-6.62	1.07E-04
				Root vs T0	-8.7	1.20E-06
				Shoot vs T0	-6.67	2.45E-04
				FolRoot vs T0	-4.95	5.09E-03
				FolShoot vs T0	-8.52	4.03E-06
MMB18_RS00385	mnmC	bifunctional tRNA (5-methylaminomethyl-2-thiouridine)(34)-methyltransferase MnmD/FAD-dependent 5-carboxymethylaminomethyl-2-thiouridine(34) oxidoreductase MnmC	GO:0008033, GO:0004808, GO:0016491	No Seed vs T0	-6.75	8.07E-09
				Root vs T0	-6.43	2.68E-08
				Shoot vs T0	-4.74	5.86E-05
				FolRoot vs T0	-3.88	6.09E-04
				FolShoot vs T0	-4.22	1.66E-04
MMB18_RS00400	NA	MarR family winged helix-turn-helix transcriptional regulator	GO:0006355, GO:0003700	No Seed vs T0	-10.2	8.91E-11
				Root vs T0	-10.1	1.31E-10
				Shoot vs T0	-4.9	4.26E-06
				FolRoot vs T0	-7.8	6.08E-08
				FolShoot vs T0	-4.79	2.33E-06
MMB18_RS00410	NA	NADH:flavin oxidoreductase/NADH oxidase	NA	No Seed vs T0	-5.72	1.49E-11
				Root vs T0	-6.18	5.43E-12
				Shoot vs T0	-4.68	6.21E-08
				FolRoot vs T0	-6.35	1.27E-10
				FolShoot vs T0	-8.77	5.06E-10
MMB18_RS00450	NA	class IV adenylate cyclase	NA	No Seed vs T0	-2.24	2.11E-03
				Root vs T0	-4.25	7.44E-09
				Shoot vs T0	-3.55	5.02E-06
				FolRoot vs T0	-2.2	3.51E-03
				FolShoot vs T0	-4.44	7.23E-09
MMB18_RS00490	NA	ABC transporter substrate-binding protein	NA	No Seed vs T0	-6.34	6.05E-08
				Root vs T0	-8.08	2.18E-07
				Shoot vs T0	-6.05	2.42E-07
				FolRoot vs T0	-2.79	3.22E-03
				FolShoot vs T0	-4.78	4.23E-06
MMB18_RS00745	NA	L-serine ammonia-lyase	GO:0006094, GO:0003941	No Seed vs T0	-5.3	4.07E-06
				Root vs T0	-5.23	5.61E-06
				Shoot vs T0	-6.01	1.20E-06
				FolRoot vs T0	-4.36	2.26E-04
				FolShoot vs T0	-7.36	3.37E-09
MMB18_RS00835	NA	hypothetical protein	NA	No Seed vs T0	-9.79	4.15E-08
				Root vs T0	-6.4	6.24E-06
				Shoot vs T0	-8.75	8.38E-07
				FolRoot vs T0	-8.26	7.25E-06
				FolShoot vs T0	-4.64	5.74E-04
MMB18_RS00850	NA	transcriptional regulator	NA	No Seed vs T0	-5.39	1.63E-03
				Root vs T0	-4.38	8.45E-03
				Shoot vs T0	-6.12	9.61E-04
				FolRoot vs T0	-5.82	2.01E-03
				FolShoot vs T0	-6.71	4.90E-04
MMB18_RS00855	NA	helix-turn-helix domain-containing protein	GO:0003677	No Seed vs T0	-8	4.84E-10
				Root vs T0	-7.39	9.11E-11
				Shoot vs T0	-8.12	2.13E-10
				FolRoot vs T0	-5.17	1.14E-09
				FolShoot vs T0	-7.7	4.93E-10
MMB18_RS00895	NA	tetratricopeptide repeat protein	NA	No Seed vs T0	-3.81	2.91E-04
				Root vs T0	-5.1	5.65E-06
				Shoot vs T0	-3.37	2.88E-03
				FolRoot vs T0	-3.93	4.11E-04
				FolShoot vs T0	-6.26	2.24E-06
MMB18_RS00920	NA	flagellin domain-containing protein	NA	No Seed vs T0	-3.24	1.42E-03
				Root vs T0	-3.59	5.14E-04
				Shoot vs T0	-3.73	9.50E-04
				FolRoot vs T0	-5.51	4.59E-05
				FolShoot vs T0	-4.02	3.07E-04
MMB18_RS00935	NA	BadF/BadG/BcrA/BcrD ATPase family protein	NA	No Seed vs T0	-5.95	8.45E-10
				Root vs T0	-7.21	2.98E-10
				Shoot vs T0	-4.8	1.21E-06
				FolRoot vs T0	-6.09	9.37E-09
				FolShoot vs T0	-5.26	7.08E-08
MMB18_RS00945	aqpZ	aquaporin Z	GO:0006833, GO:0015250	No Seed vs T0	-5.63	2.10E-03
				Root vs T0	-6.53	4.89E-04

				Shoot vs T0	-6.34	1.19E-03
				FolRoot vs T0	-5.74	2.71E-03
				FolShoot vs T0	-7.66	1.62E-04
MMB18_RS01045	NA	DUF805 domain-containing protein	GO:0016020	No Seed vs T0	-7.92	5.42E-06
				Root vs T0	-6.61	1.29E-05
				Shoot vs T0	-8.61	2.20E-06
				FolRoot vs T0	-8.13	1.61E-05
				FolShoot vs T0	-9.04	9.11E-07
MMB18_RS01060	flhF	flagellar biosynthesis protein FlhF	GO:0044781, GO:0003924, GO:0005525	No Seed vs T0	-4.04	2.69E-05
				Root vs T0	-4.26	1.72E-05
				Shoot vs T0	-3.07	2.08E-03
				FolRoot vs T0	-2.57	6.60E-03
				FolShoot vs T0	-2.41	9.71E-03
MMB18_RS01090	NA	amidase	GO:0003824	No Seed vs T0	-6.07	1.86E-06
				Root vs T0	-5.34	1.16E-05
				Shoot vs T0	-3.33	6.81E-03
				FolRoot vs T0	-5.06	6.40E-05
				FolShoot vs T0	-6.95	2.01E-06
MMB18_RS01105	NA	branched-chain amino acid ABC transporter substrate-binding protein	GO:0015803, GO:0055085, GO:0015658, GO:0022857, GO:0140359	No Seed vs T0	-4.18	1.55E-07
				Root vs T0	-8.21	2.80E-08
				Shoot vs T0	-3.03	2.48E-04
				FolRoot vs T0	-2.91	2.44E-04
				FolShoot vs T0	-4.5	2.35E-07
MMB18_RS01190	NA	OB-fold-containig protein	NA	No Seed vs T0	-7.68	3.19E-08
				Root vs T0	-2.87	6.05E-07
				Shoot vs T0	-5.22	6.75E-10
				FolRoot vs T0	-2.65	1.24E-05
				FolShoot vs T0	-3.21	3.55E-07
MMB18_RS01195	NA	flotillin family protein	GO:0016020, GO:0016600	No Seed vs T0	-5.68	4.74E-09
				Root vs T0	-4.95	2.00E-07
				Shoot vs T0	-4.42	1.20E-05
				FolRoot vs T0	-5.71	2.04E-08
				FolShoot vs T0	-5.42	6.31E-08
MMB18_RS01295	NA	YbhB/YbcL family Raf kinase inhibitor-like protein	NA	No Seed vs T0	-7.67	8.87E-07
				Root vs T0	-6.09	1.27E-06
				Shoot vs T0	-5.29	3.97E-06
				FolRoot vs T0	-7.1	9.20E-06
				FolShoot vs T0	-6.45	2.20E-06
MMB18_RS01345	paaE	1,2-phenylacetyl-CoA epoxidase subunit PaaE	GO:0010124	No Seed vs T0	-4.28	6.96E-07
				Root vs T0	-3.77	7.34E-06
				Shoot vs T0	-4.77	9.73E-07
				FolRoot vs T0	-2.73	1.28E-03
				FolShoot vs T0	-5.81	1.76E-07
MMB18_RS01365	paaA	1,2-phenylacetyl-CoA epoxidase subunit PaaA	GO:0010124, GO:0097266	No Seed vs T0	-5.21	6.56E-08
				Root vs T0	-8.38	5.50E-08
				Shoot vs T0	-6.46	3.00E-08
				FolRoot vs T0	-3.99	2.63E-05
				FolShoot vs T0	-8.82	1.09E-08
MMB18_RS01370	NA	SGNH/GDSL hydrolase family protein	GO:0016788	No Seed vs T0	-5.28	7.07E-06
				Root vs T0	-7.99	4.22E-10
				Shoot vs T0	-7.61	1.00E-08
				FolRoot vs T0	-5.03	3.20E-05
				FolShoot vs T0	-6.72	7.05E-08
MMB18_RS01670	NA	c-type cytochrome biogenesis protein CcsB	GO:0017004, GO:0020037	No Seed vs T0	-4.56	6.92E-06
				Root vs T0	-3.83	3.00E-05
				Shoot vs T0	-4.49	1.72E-05
				FolRoot vs T0	-4.26	9.76E-05
				FolShoot vs T0	-4.49	3.28E-05
MMB18_RS01720	aroK	shikimate kinase AroK	NA	No Seed vs T0	-5.6	9.07E-05
				Root vs T0	-4.94	4.90E-04
				Shoot vs T0	-7.08	8.94E-06
				FolRoot vs T0	-6.38	3.71E-05
				FolShoot vs T0	-6.3	3.66E-05
MMB18_RS01725	aroB	3-dehydroquinate synthase	GO:0009073, GO:0009423, GO:0003856, GO:0005737	No Seed vs T0	-4.79	2.88E-14
				Root vs T0	-4.12	4.49E-11
				Shoot vs T0	-3.81	8.92E-09
				FolRoot vs T0	-4.18	7.10E-11
				FolShoot vs T0	-4.41	7.71E-12
MMB18_RS01730	NA	deoxyguanosinetriphosphate triphosphohydrolase	GO:0016793	No Seed vs T0	-4.01	9.68E-09
				Root vs T0	-3.13	6.65E-06
				Shoot vs T0	-3.48	3.14E-06

				FolRoot vs T0	-2.47	5.66E-04
				FolShoot vs T0	-4.68	3.20E-10
MMB18_RS01735	ugpB	sn-glycerol-3-phosphate ABC transporter substrate-binding protein UgpB	GO:0055085	No Seed vs T0	-5.68	1.60E-05
				Root vs T0	-7.41	1.42E-06
				Shoot vs T0	-6.77	4.62E-06
				FolRoot vs T0	-4.58	5.66E-04
				FolShoot vs T0	-5.34	8.16E-05
MMB18_RS01770	NA	glutamate synthase-related protein	GO:0006537, GO:0015930, GO:0016638	No Seed vs T0	-4.8	1.50E-21
				Root vs T0	-4.96	4.50E-23
				Shoot vs T0	-4.29	2.08E-16
				FolRoot vs T0	-4.23	9.77E-17
				FolShoot vs T0	-4.4	7.28E-18
MMB18_RS01790	NA	alanine/glycine:cation symporter family protein	GO:0006814, GO:0006865, GO:0005283, GO:0015293, GO:0016020	No Seed vs T0	-4.3	1.76E-03
				Root vs T0	-4.25	2.02E-03
				Shoot vs T0	-4.38	2.76E-03
				FolRoot vs T0	-5.99	5.05E-05
				FolShoot vs T0	-6.26	2.55E-05
MMB18_RS01830	NA	thiazole synthase	GO:1990107	No Seed vs T0	-3.74	5.89E-08
				Root vs T0	-4.42	5.44E-10
				Shoot vs T0	-4.12	7.06E-08
				FolRoot vs T0	-3.95	6.08E-08
				FolShoot vs T0	-4.26	8.90E-09
MMB18_RS01885	murA	UDP-N-acetylglucosamine 1-carboxyvinyltransferase	GO:0019277, GO:0008760	No Seed vs T0	-7	2.13E-14
				Root vs T0	-8.38	1.52E-15
				Shoot vs T0	-5.56	3.17E-09
				FolRoot vs T0	-6.4	6.59E-12
				FolShoot vs T0	-6.58	2.58E-12
MMB18_RS01890	hisG	ATP phosphoribosyltransferase	GO:0000105, GO:0003879, GO:0005737	No Seed vs T0	-7.26	1.28E-03
				Root vs T0	-11.2	9.26E-06
				Shoot vs T0	-8.49	4.22E-04
				FolRoot vs T0	-8.98	3.62E-04
				FolShoot vs T0	-6.12	7.23E-03
MMB18_RS01895	hisD	histidinol dehydrogenase	GO:0000105, GO:0004399, GO:0046872, GO:0051287	No Seed vs T0	-5.46	3.35E-21
				Root vs T0	-5.49	2.87E-21
				Shoot vs T0	-5.55	1.48E-18
				FolRoot vs T0	-5.6	1.49E-20
				FolShoot vs T0	-5.24	2.21E-18
MMB18_RS01905	hisB	imidazoleglycerol-phosphate dehydratase HisB	GO:0000105, GO:0004424	No Seed vs T0	-9.29	1.02E-04
				Root vs T0	-11.4	5.88E-06
				Shoot vs T0	-8.7	2.85E-04
				FolRoot vs T0	-9.97	1.19E-04
				FolShoot vs T0	-10.9	1.71E-05
MMB18_RS01915	hisH	imidazole glycerol phosphate synthase subunit HisH	GO:0000105, GO:0000107	No Seed vs T0	-9.09	3.27E-08
				Root vs T0	-10.4	2.78E-08
				Shoot vs T0	-6.56	1.69E-05
				FolRoot vs T0	-8.49	3.96E-07
				FolShoot vs T0	-8.17	3.30E-07
MMB18_RS01920	hisA	1-(5-phosphoribosyl)-5-[(5-phosphoribosylamino)methylideneamino]imidazole-4-carboxamide isomerase	GO:0000105, GO:0003949	No Seed vs T0	-4.11	6.73E-13
				Root vs T0	-3.96	4.80E-12
				Shoot vs T0	-5.07	5.29E-16
				FolRoot vs T0	-5.12	1.41E-17
				FolShoot vs T0	-4.3	2.64E-13
MMB18_RS01925	hisF	imidazole glycerol phosphate synthase subunit HisF	GO:0000105, GO:0000107	No Seed vs T0	-2.91	3.04E-05
				Root vs T0	-2.59	2.10E-04
				Shoot vs T0	-2.44	9.50E-04
				FolRoot vs T0	-2.41	8.08E-04
				FolShoot vs T0	-2.47	5.13E-04
MMB18_RS01930	hisI	phosphoribosyl-AMP cyclohydrolase	GO:0000105, GO:0004635	No Seed vs T0	-10.4	2.27E-10
				Root vs T0	-9.34	1.36E-08
				Shoot vs T0	-9.38	6.13E-09
				FolRoot vs T0	-8.07	1.37E-07
				FolShoot vs T0	-8.25	2.18E-08
MMB18_RS01935	NA	phosphoribosyl-ATP diphosphatase	GO:0000105, GO:0004636	No Seed vs T0	-8.54	3.32E-05
				Root vs T0	-8.43	4.26E-05
				Shoot vs T0	-4.89	7.22E-03
				FolRoot vs T0	-7.96	1.62E-04
				FolShoot vs T0	-5.02	5.99E-03
MMB18_RS01950	tatA	Sec-independent protein translocase subunit TatA	GO:0043953, GO:0016020	No Seed vs T0	-8.09	1.18E-04
				Root vs T0	-5.13	5.65E-03
				Shoot vs T0	-6	2.63E-03
				FolRoot vs T0	-7.52	4.97E-04

				FolShoot vs T0	-7.47	4.14E-04
MMB18_RS02050	NA	RHS repeat-associated core domain-containing protein	NA	No Seed vs T0	-5.39	3.83E-06
				Root vs T0	-7.95	3.92E-11
				Shoot vs T0	-5.74	2.70E-06
				FolRoot vs T0	-6.46	6.79E-08
				FolShoot vs T0	-6.92	8.90E-09
MMB18_RS02080	NA	hypothetical protein	NA	No Seed vs T0	-6.16	4.51E-04
				Root vs T0	-10.4	1.17E-06
				Shoot vs T0	-10.4	1.06E-06
				FolRoot vs T0	-10.9	5.40E-07
				FolShoot vs T0	-8.63	9.78E-06
MMB18_RS02090	NA	hypothetical protein	NA	No Seed vs T0	-5.34	2.78E-03
				Root vs T0	-6.77	2.63E-04
				Shoot vs T0	-5.99	1.70E-03
				FolRoot vs T0	-6.39	8.26E-04
				FolShoot vs T0	-7.71	1.15E-04
MMB18_RS02210	NA	transporter	NA	No Seed vs T0	-2.65	9.44E-04
				Root vs T0	-2.67	8.76E-04
				Shoot vs T0	-3	5.16E-04
				FolRoot vs T0	-2.55	2.16E-03
				FolShoot vs T0	-2.16	8.56E-03
MMB18_RS02240	NA	methyltransferase	GO:0008168	No Seed vs T0	-3.93	6.92E-08
				Root vs T0	-4.71	1.27E-10
				Shoot vs T0	-4.27	3.31E-08
				FolRoot vs T0	-3.88	2.11E-07
				FolShoot vs T0	-5.23	5.37E-12
MMB18_RS02245	NA	class I SAM-dependent methyltransferase	GO:0032259, GO:0008168, GO:0008757, GO:1904047	No Seed vs T0	-3.39	1.52E-07
				Root vs T0	-3.21	6.33E-07
				Shoot vs T0	-2.78	4.83E-05
				FolRoot vs T0	-3.1	3.20E-06
				FolShoot vs T0	-3.14	1.88E-06
MMB18_RS02300	prmC	peptide chain release factor N(5)-glutamine methyltransferase	GO:0006412, GO:0018364, GO:0008757	No Seed vs T0	-7.35	8.09E-09
				Root vs T0	-5.4	1.56E-10
				Shoot vs T0	-5.41	9.00E-10
				FolRoot vs T0	-6.19	2.54E-08
				FolShoot vs T0	-6.38	2.90E-09
MMB18_RS02315	NA	response regulator transcription factor	GO:0000160, GO:0006355, GO:0000156, GO:0003677	No Seed vs T0	-5.01	9.12E-05
				Root vs T0	-5.19	9.06E-05
				Shoot vs T0	-4	1.55E-03
				FolRoot vs T0	-3.88	1.93E-03
				FolShoot vs T0	-3.8	1.98E-03
MMB18_RS02365	gabP	GABA permease	GO:0015812, GO:0015185, GO:0016020	No Seed vs T0	-6.29	3.52E-08
				Root vs T0	-4.98	3.93E-06
				Shoot vs T0	-4.87	2.75E-05
				FolRoot vs T0	-4.77	1.90E-05
				FolShoot vs T0	-6.08	3.32E-07
MMB18_RS02385	NA	hypothetical protein	NA	No Seed vs T0	-8.5	6.23E-09
				Root vs T0	-6.92	3.29E-08
				Shoot vs T0	-4.96	7.12E-05
				FolRoot vs T0	-6.6	3.34E-07
				FolShoot vs T0	-5.29	1.15E-05
MMB18_RS02410	NA	hypothetical protein	NA	No Seed vs T0	-5.02	1.67E-08
				Root vs T0	-5.72	1.78E-10
				Shoot vs T0	-6.41	8.94E-11
				FolRoot vs T0	-4.92	6.91E-08
				FolShoot vs T0	-6.18	4.15E-11
MMB18_RS02415	NA	hypothetical protein	NA	No Seed vs T0	-6.2	3.27E-04
				Root vs T0	-5.94	5.81E-04
				Shoot vs T0	-7.54	5.38E-05
				FolRoot vs T0	-5.19	3.55E-03
				FolShoot vs T0	-6.59	2.22E-04
MMB18_RS02425	NA	nucleoside 2-deoxyribosyltransferase	NA	No Seed vs T0	-8.66	1.93E-10
				Root vs T0	-9.38	3.41E-10
				Shoot vs T0	-9.43	1.02E-10
				FolRoot vs T0	-4.35	2.22E-07
				FolShoot vs T0	-5.12	4.37E-09
MMB18_RS02440	NA	metallophosphoesterase	GO:0016787	No Seed vs T0	-6.47	1.84E-07
				Root vs T0	-7.71	4.31E-10
				Shoot vs T0	-5.38	2.50E-05
				FolRoot vs T0	-5.96	2.78E-06
				FolShoot vs T0	-7.66	9.63E-10

MMB18_RS02455	NA	HNH endonuclease	GO:0003676, GO:0004519, GO:0008270	No Seed vs T0	-7.99	2.44E-07
				Root vs T0	-7.78	4.31E-07
				Shoot vs T0	-9.4	9.76E-08
				FolRoot vs T0	-10.9	1.89E-08
				FolShoot vs T0	-7.89	1.13E-06
MMB18_RS02460	NA	OmpA/MotB family protein	GO:0015288, GO:0042834	No Seed vs T0	-7.19	7.63E-04
				Root vs T0	-7.3	6.96E-04
				Shoot vs T0	-6.26	4.77E-03
				FolRoot vs T0	-7.64	8.58E-04
				FolShoot vs T0	-10.1	3.03E-05
MMB18_RS02470	rmuC	DNA recombination protein RmuC	NA	No Seed vs T0	-4.25	2.62E-05
				Root vs T0	-6.36	2.45E-10
				Shoot vs T0	-6.43	1.62E-09
				FolRoot vs T0	-5.12	6.15E-07
				FolShoot vs T0	-6.35	5.20E-10
MMB18_RS02500	NA	hypothetical protein	NA	No Seed vs T0	-9.21	1.34E-08
				Root vs T0	-5.27	1.33E-05
				Shoot vs T0	-5.04	9.45E-05
				FolRoot vs T0	-8.37	1.19E-07
				FolShoot vs T0	-10.4	2.40E-09
MMB18_RS02505	NA	helix-turn-helix domain-containing protein	GO:0003677	No Seed vs T0	-7.36	1.43E-04
				Root vs T0	-6.21	4.14E-04
				Shoot vs T0	-4.51	9.89E-03
				FolRoot vs T0	-6.31	8.58E-04
				FolShoot vs T0	-8.55	2.73E-05
MMB18_RS02545	trpC	indole-3-glycerol phosphate synthase TrpC	GO:0006568, GO:0004425	No Seed vs T0	-7.61	1.33E-06
				Root vs T0	-9.32	3.41E-08
				Shoot vs T0	-5.52	2.44E-05
				FolRoot vs T0	-3.6	2.63E-03
				FolShoot vs T0	-8.8	2.21E-07
MMB18_RS02550	trpD	anthranilate phosphoribosyltransferase	GO:0000162, GO:0004048	No Seed vs T0	-3.32	4.89E-07
				Root vs T0	-3.81	9.77E-09
				Shoot vs T0	-3.72	2.04E-07
				FolRoot vs T0	-2.51	2.31E-04
				FolShoot vs T0	-3.04	6.64E-06
MMB18_RS02570	rpe	ribulose-phosphate 3-epimerase	GO:0006098, GO:0004750	No Seed vs T0	-10	1.73E-08
				Root vs T0	-6.04	7.55E-06
				Shoot vs T0	-4.5	1.14E-03
				FolRoot vs T0	-6.47	1.25E-05
				FolShoot vs T0	-8.37	1.13E-06
MMB18_RS02600	pcaF	3-oxoadipyl-CoA thiolase	GO:0019619, GO:0016747	No Seed vs T0	-5.75	4.89E-09
				Root vs T0	-5.8	5.10E-09
				Shoot vs T0	-4.49	8.58E-06
				FolRoot vs T0	-5.54	7.53E-08
				FolShoot vs T0	-5.97	2.04E-08
MMB18_RS02610	NA	enoyl-CoA hydratase	NA	No Seed vs T0	-6.65	4.16E-11
				Root vs T0	-5.8	1.00E-09
				Shoot vs T0	-7.07	2.22E-10
				FolRoot vs T0	-5.38	3.29E-08
				FolShoot vs T0	-7.86	2.33E-10
MMB18_RS02640	NA	molybdopterin-containing oxidoreductase family protein	GO:0016491, GO:0043546	No Seed vs T0	-4.63	4.72E-18
				Root vs T0	-4.64	4.96E-18
				Shoot vs T0	-5.15	3.37E-18
				FolRoot vs T0	-5.06	3.05E-19
				FolShoot vs T0	-4.31	4.51E-15
MMB18_RS02785	NA	prepilin peptidase	GO:0004190, GO:0016020	No Seed vs T0	-7.68	2.11E-08
				Root vs T0	-10.4	9.25E-10
				Shoot vs T0	-5.9	4.88E-06
				FolRoot vs T0	-6.49	8.45E-07
				FolShoot vs T0	-9.87	7.90E-09
MMB18_RS02800	NA	HlyC/CorC family transporter	GO:0016020	No Seed vs T0	-5.14	6.69E-03
				Root vs T0	-6.09	1.28E-03
				Shoot vs T0	-6.35	1.55E-03
				FolRoot vs T0	-7.47	1.76E-04
				FolShoot vs T0	-5.26	6.05E-03
MMB18_RS03045	NA	phosphatidylglycerophosphatase A	NA	No Seed vs T0	-3.79	9.98E-03
				Root vs T0	-6.04	1.27E-04
				Shoot vs T0	-5.08	1.49E-03
				FolRoot vs T0	-4.84	1.93E-03
				FolShoot vs T0	-5.57	5.15E-04
MMB18_RS03090	gap	type I glyceraldehyde-3-phosphate	GO:0006006, GO:0016620,	No Seed vs T0	-5.57	4.18E-11

		dehydrogenase	GO:0050661, GO:0051287	Root vs T0	-4.33	4.33E-08
				Shoot vs T0	-4.54	1.67E-07
				FolRoot vs T0	-2.74	5.70E-04
				FolShoot vs T0	-4.85	1.08E-08
MMB18_RS03305	erpA	iron-sulfur cluster insertion protein ErpA	GO:0016226, GO:0051536	No Seed vs T0	-7.11	3.46E-06
				Root vs T0	-6.07	9.42E-06
				Shoot vs T0	-6.28	3.97E-06
				FolRoot vs T0	-6.54	3.13E-05
				FolShoot vs T0	-4.88	2.07E-05
MMB18_RS03335	NA	oxygenase MpaB family protein	GO:0016491	No Seed vs T0	-3.46	5.83E-07
				Root vs T0	-2.76	5.41E-05
				Shoot vs T0	-3.59	2.39E-06
				FolRoot vs T0	-3.24	7.97E-06
				FolShoot vs T0	-3.61	7.89E-07
MMB18_RS03430	NA	THUMP domain-containing class I SAM-dependent RNA methyltransferase	GO:0001510, GO:0008757, GO:1904047	No Seed vs T0	-3.8	5.27E-04
				Root vs T0	-4.11	1.85E-04
				Shoot vs T0	-5.31	5.66E-06
				FolRoot vs T0	-3.38	2.92E-03
				FolShoot vs T0	-4.24	1.34E-04
MMB18_RS03435	NA	site-specific recombinase	NA	No Seed vs T0	-3.6	1.88E-10
				Root vs T0	-3.67	9.47E-11
				Shoot vs T0	-3.99	1.20E-10
				FolRoot vs T0	-3.97	2.11E-11
				FolShoot vs T0	-3.62	5.58E-10
MMB18_RS03450	NA	paraquat-inducible protein A	NA	No Seed vs T0	-5.57	3.57E-04
				Root vs T0	-9.6	8.76E-07
				Shoot vs T0	-6.71	9.86E-05
				FolRoot vs T0	-5.04	1.76E-03
				FolShoot vs T0	-7.58	3.45E-05
MMB18_RS03455	NA	paraquat-inducible protein A	NA	No Seed vs T0	-6.63	5.60E-07
				Root vs T0	-8.01	4.54E-07
				Shoot vs T0	-6.46	3.08E-06
				FolRoot vs T0	-5.87	1.19E-05
				FolShoot vs T0	-5.58	1.89E-05
MMB18_RS03460	NA	cytochrome b	GO:0022904, GO:0009055, GO:0020037, GO:0046872, GO:0016020	No Seed vs T0	-6.65	1.04E-09
				Root vs T0	-9.4	3.62E-09
				Shoot vs T0	-8.54	1.57E-09
				FolRoot vs T0	-9.89	7.75E-10
				FolShoot vs T0	-9.85	6.33E-10
MMB18_RS03540	NA	YggS family pyridoxal phosphate-dependent enzyme	GO:0030170	No Seed vs T0	-7.12	1.73E-06
				Root vs T0	-6.99	2.84E-06
				Shoot vs T0	-4.51	9.18E-05
				FolRoot vs T0	-4.21	1.68E-04
				FolShoot vs T0	-7.39	1.93E-06
MMB18_RS03560	NA	FAD-binding oxidoreductase	GO:0016491, GO:0071949	No Seed vs T0	-3.75	4.36E-07
				Root vs T0	-2.54	3.99E-04
				Shoot vs T0	-3.09	8.97E-05
				FolRoot vs T0	-4.41	1.20E-07
				FolShoot vs T0	-4.9	1.92E-08
MMB18_RS03570	hmpA	NO-inducible flavohemoprotein	GO:0051409, GO:0008941, GO:0019825, GO:0020037, GO:0071949	No Seed vs T0	-5.63	7.33E-09
				Root vs T0	-6.01	5.56E-09
				Shoot vs T0	-5.02	5.32E-07
				FolRoot vs T0	-3.28	2.52E-04
				FolShoot vs T0	-4.63	1.08E-06
MMB18_RS03610	glnE	bifunctional [glutamate--ammonia ligase]-adenylyl-L-tyrosine phosphorylase/[glutamate--ammonia-ligase] adenylyltransferase	GO:0008882	No Seed vs T0	-4.43	6.01E-04
				Root vs T0	-6.15	2.82E-06
				Shoot vs T0	-5.37	1.02E-04
				FolRoot vs T0	-3.73	5.37E-03
				FolShoot vs T0	-3.94	2.67E-03
MMB18_RS03645	grpE	nucleotide exchange factor GrpE	GO:0006457, GO:0000774, GO:0051087	No Seed vs T0	-6.37	2.94E-06
				Root vs T0	-4.56	6.03E-05
				Shoot vs T0	-3.33	4.42E-03
				FolRoot vs T0	-3.56	1.83E-03
				FolShoot vs T0	-5.38	2.94E-05
MMB18_RS03670	panB	3-methyl-2-oxobutanoate hydroxymethyltransferase	GO:0015940, GO:0003864	No Seed vs T0	-3.34	1.12E-05
				Root vs T0	-7.34	5.15E-07
				Shoot vs T0	-3.75	1.15E-05
				FolRoot vs T0	-6.05	4.50E-06
				FolShoot vs T0	-5.57	4.57E-07
MMB18_RS03690	NA	HAD family hydrolase	GO:0016787	No Seed vs T0	-4.94	1.55E-04
				Root vs T0	-6.23	3.30E-06

				Shoot vs T0	-8.51	7.77E-08
				FolRoot vs T0	-7.74	5.62E-07
				FolShoot vs T0	-9.36	7.94E-08
MMB18_RS03715	tetR	tetracycline resistance transcriptional repressor TetR	NA	No Seed vs T0	-3.88	1.13E-06
				Root vs T0	-3.5	1.13E-05
				Shoot vs T0	-4.94	2.70E-08
				FolRoot vs T0	-3.33	4.96E-05
				FolShoot vs T0	-3.11	1.26E-04
MMB18_RS03735	NA	cupin domain-containing protein	NA	No Seed vs T0	-8.33	8.00E-16
				Root vs T0	-7.82	4.78E-17
				Shoot vs T0	-7.1	2.19E-18
				FolRoot vs T0	-9.2	4.60E-11
				FolShoot vs T0	-7.64	5.98E-16
MMB18_RS03750	NA	capsular polysaccharide biosynthesis protein	NA	No Seed vs T0	-7.15	1.78E-13
				Root vs T0	-6.83	1.02E-12
				Shoot vs T0	-6.17	9.37E-10
				FolRoot vs T0	-4.54	1.16E-06
				FolShoot vs T0	-7.04	4.13E-12
MMB18_RS03820	NA	glycosyltransferase family 2 protein	GO:0006486, GO:0016757	No Seed vs T0	-7.19	6.14E-15
				Root vs T0	-6.93	4.34E-14
				Shoot vs T0	-7.12	5.46E-13
				FolRoot vs T0	-6.19	2.99E-11
				FolShoot vs T0	-6.55	2.35E-12
MMB18_RS03825	NA	glycosyltransferase family 2 protein	GO:0006486, GO:0016757	No Seed vs T0	-5.85	3.25E-05
				Root vs T0	-5.99	2.05E-05
				Shoot vs T0	-7.44	5.11E-07
				FolRoot vs T0	-7.5	1.91E-07
				FolShoot vs T0	-6.85	1.37E-06
MMB18_RS03830	NA	phytanoyl-CoA dioxygenase family protein	GO:0046872, GO:0051213	No Seed vs T0	-3.15	2.25E-10
				Root vs T0	-3.12	3.03E-10
				Shoot vs T0	-4.27	2.08E-16
				FolRoot vs T0	-4.52	2.19E-19
				FolShoot vs T0	-4.33	9.40E-18
MMB18_RS03835	NA	capsule biosynthesis protein	NA	No Seed vs T0	-9.82	1.06E-05
				Root vs T0	-7.68	2.03E-04
				Shoot vs T0	-7.13	1.02E-03
				FolRoot vs T0	-7.46	4.64E-04
				FolShoot vs T0	-6.25	2.60E-03
MMB18_RS03840	NA	SDR family NAD(P)-dependent oxidoreductase	GO:0016491, GO:0070401, GO:0070403	No Seed vs T0	-5.56	3.07E-03
				Root vs T0	-6.7	4.15E-04
				Shoot vs T0	-7.22	3.47E-04
				FolRoot vs T0	-5.83	2.74E-03
				FolShoot vs T0	-8.49	4.23E-05
MMB18_RS03865	NA	GNAT family N-acetyltransferase	GO:0008080, GO:0016746	No Seed vs T0	-6.97	2.92E-05
				Root vs T0	-5.41	7.42E-04
				Shoot vs T0	-8.73	4.96E-06
				FolRoot vs T0	-6.6	1.35E-04
				FolShoot vs T0	-4.87	2.80E-03
MMB18_RS03885	NA	cupin domain-containing protein	NA	No Seed vs T0	-5.26	4.26E-11
				Root vs T0	-5.29	7.65E-11
				Shoot vs T0	-3.11	2.45E-05
				FolRoot vs T0	-4.83	5.22E-09
				FolShoot vs T0	-3.37	2.52E-06
MMB18_RS03910	NA	hypothetical protein	NA	No Seed vs T0	-5.11	1.26E-33
				Root vs T0	-4.94	3.90E-32
				Shoot vs T0	-4.97	1.98E-28
				FolRoot vs T0	-5.44	7.62E-27
				FolShoot vs T0	-6.43	1.87E-25
MMB18_RS03955	tolQ	protein TolQ	GO:0043213, GO:0016020	No Seed vs T0	-5.31	9.17E-06
				Root vs T0	-5.8	1.83E-06
				Shoot vs T0	-5.26	4.64E-05
				FolRoot vs T0	-4.03	9.44E-04
				FolShoot vs T0	-6.63	5.61E-07
MMB18_RS03970	glyA	serine hydroxymethyltransferase	GO:0019264, GO:0004372, GO:0008270, GO:0030170, GO:0070905	No Seed vs T0	-2.99	2.57E-04
				Root vs T0	-3.2	1.15E-04
				Shoot vs T0	-4.38	6.46E-06
				FolRoot vs T0	-2.22	8.71E-03
				FolShoot vs T0	-3.74	3.66E-05
MMB18_RS04025	NA	acyl-CoA dehydrogenase	NA	No Seed vs T0	-7.17	7.87E-11
				Root vs T0	-5.94	1.53E-09
				Shoot vs T0	-5.59	7.98E-08

				FolRoot vs T0	-5.09	2.22E-07
				FolShoot vs T0	-6.02	8.38E-09
MMB18_RS04075	NA	MFS transporter	GO:0055085, GO:0022857, GO:0005886, GO:0016020	No Seed vs T0	-5.7	8.03E-15
				Root vs T0	-6.7	1.19E-14
				Shoot vs T0	-5.11	1.62E-11
				FolRoot vs T0	-5.7	1.39E-12
				FolShoot vs T0	-6.88	6.92E-13
MMB18_RS04105	NA	helix-turn-helix domain-containing protein	GO:0006355, GO:0003700, GO:0043565	No Seed vs T0	-3.86	4.07E-04
				Root vs T0	-5.24	3.15E-06
				Shoot vs T0	-4.62	1.03E-04
				FolRoot vs T0	-3.56	1.62E-03
				FolShoot vs T0	-4.74	3.33E-05
MMB18_RS04140	NA	LysR family transcriptional regulator	GO:0001216, GO:0003677, GO:0003700	No Seed vs T0	-6.19	3.60E-08
				Root vs T0	-5.95	6.87E-08
				Shoot vs T0	-3.38	6.51E-04
				FolRoot vs T0	-3.65	1.54E-04
				FolShoot vs T0	-4.79	3.14E-06
MMB18_RS04145	NA	2-hydroxychromene-2-carboxylate isomerase	GO:1901170, GO:0018845, GO:0043295	No Seed vs T0	-4.79	1.64E-04
				Root vs T0	-6.7	6.66E-06
				Shoot vs T0	-7.31	4.59E-06
				FolRoot vs T0	-5.16	1.77E-04
				FolShoot vs T0	-7.67	5.81E-06
MMB18_RS04150	NA	TAXI family TRAP transporter solute-binding subunit	NA	No Seed vs T0	-6.45	6.91E-10
				Root vs T0	-5.11	1.07E-07
				Shoot vs T0	-4.41	1.35E-05
				FolRoot vs T0	-4.95	7.28E-07
				FolShoot vs T0	-4.77	1.26E-06
MMB18_RS04160	NA	D-(-)-3-hydroxybutyrate oligomer hydrolase	NA	No Seed vs T0	-4.45	2.38E-13
				Root vs T0	-5.86	5.49E-16
				Shoot vs T0	-6.04	8.43E-15
				FolRoot vs T0	-4.2	3.32E-11
				FolShoot vs T0	-3.69	1.42E-09
MMB18_RS04180	NA	DsbA family oxidoreductase	GO:0015036	No Seed vs T0	-6.59	9.68E-12
				Root vs T0	-8.64	2.49E-09
				Shoot vs T0	-4.89	3.94E-09
				FolRoot vs T0	-6.32	1.17E-09
				FolShoot vs T0	-7.5	9.18E-10
MMB18_RS04270	groES	co-chaperone GroES	GO:0006457, GO:0005524, GO:0016887	No Seed vs T0	-9.63	2.20E-06
				Root vs T0	-8.56	2.84E-05
				Shoot vs T0	-5.04	4.15E-03
				FolRoot vs T0	-6.22	6.35E-04
				FolShoot vs T0	-9	1.07E-05
MMB18_RS04280	NA	hypothetical protein	NA	No Seed vs T0	-5.76	3.34E-08
				Root vs T0	-7.04	8.92E-10
				Shoot vs T0	-5.17	3.19E-06
				FolRoot vs T0	-5.1	1.74E-06
				FolShoot vs T0	-5.04	1.80E-06
MMB18_RS04375	NA	ABC transporter permease	GO:0055085, GO:0042626, GO:0140359, GO:0016020, GO:0043190	No Seed vs T0	-8.01	1.88E-04
				Root vs T0	-11.1	3.35E-06
				Shoot vs T0	-6.98	1.55E-03
				FolRoot vs T0	-7.26	9.24E-04
				FolShoot vs T0	-10.6	1.04E-05
MMB18_RS04380	NA	ABC transporter ATP-binding protein	NA	No Seed vs T0	-6.88	3.19E-23
				Root vs T0	-7.8	6.92E-23
				Shoot vs T0	-6.27	2.19E-18
				FolRoot vs T0	-6.45	9.66E-20
				FolShoot vs T0	-5.72	1.03E-17
MMB18_RS04430	NA	protease pro-enzyme activation domain-containing protein	GO:0008236	No Seed vs T0	-5.14	2.18E-07
				Root vs T0	-5.18	2.08E-07
				Shoot vs T0	-4.9	3.97E-06
				FolRoot vs T0	-3.41	7.69E-04
				FolShoot vs T0	-5.37	1.98E-07
MMB18_RS04435	NA	MraY family glycosyltransferase	GO:0009252, GO:0016780, GO:0046872	No Seed vs T0	-8.16	7.66E-06
				Root vs T0	-9.35	3.35E-06
				Shoot vs T0	-6.46	1.96E-04
				FolRoot vs T0	-5.46	1.01E-03
				FolShoot vs T0	-4.25	8.65E-03
MMB18_RS04445	NA	glycosyltransferase family 4 protein	GO:0006486, GO:0016757	No Seed vs T0	-7.04	3.71E-04
				Root vs T0	-5.82	2.52E-03
				Shoot vs T0	-5.35	8.36E-03
				FolRoot vs T0	-6.48	1.49E-03

				FolShoot vs T0	-5.97	2.63E-03
MMB18_RS04550	NA	hypothetical protein	NA	No Seed vs T0	-8.2	3.34E-08
				Root vs T0	-6.72	2.38E-07
				Shoot vs T0	-5.21	7.79E-05
				FolRoot vs T0	-4.04	1.24E-03
				FolShoot vs T0	-6.96	5.72E-07
MMB18_RS04560	NA	YkgJ family cysteine cluster protein	GO:0051536	No Seed vs T0	-6.98	3.86E-06
				Root vs T0	-5.54	6.31E-06
				Shoot vs T0	-6.97	2.79E-06
				FolRoot vs T0	-5.77	3.07E-05
				FolShoot vs T0	-7.16	4.67E-06
MMB18_RS04650	NA	hypothetical protein	NA	No Seed vs T0	-5.52	5.44E-05
				Root vs T0	-7.08	1.80E-05
				Shoot vs T0	-4.59	1.10E-03
				FolRoot vs T0	-4.64	7.75E-04
				FolShoot vs T0	-4.42	1.10E-03
MMB18_RS04690	NA	DUF1993 domain-containing protein	NA	No Seed vs T0	-7.93	4.27E-09
				Root vs T0	-5.21	1.84E-09
				Shoot vs T0	-5.38	1.12E-08
				FolRoot vs T0	-4.15	1.04E-06
				FolShoot vs T0	-8.19	8.04E-09
MMB18_RS04710	NA	Bcr/CflA family multidrug efflux MFS transporter	GO:0042908, GO:1990961, GO:0042910, GO:0016020	No Seed vs T0	-3.02	1.20E-03
				Root vs T0	-6.51	1.89E-11
				Shoot vs T0	-5.84	1.06E-08
				FolRoot vs T0	-5.38	2.49E-08
				FolShoot vs T0	-6.68	5.96E-11
MMB18_RS04765	NA	DNA translocase FtsK	GO:0007059, GO:0051301, GO:0003677, GO:0005524, GO:0015616	No Seed vs T0	-2.97	4.10E-03
				Root vs T0	-6.84	4.44E-11
				Shoot vs T0	-6.14	1.89E-08
				FolRoot vs T0	-4.53	1.45E-05
				FolShoot vs T0	-5.62	6.50E-08
MMB18_RS04830	NA	chloride channel protein	GO:0006821, GO:0005247	No Seed vs T0	-3.47	1.71E-19
				Root vs T0	-3.27	1.65E-17
				Shoot vs T0	-3.56	3.32E-17
				FolRoot vs T0	-2.52	7.10E-11
				FolShoot vs T0	-2.64	1.08E-11
MMB18_RS04835	waaC	lipopolysaccharide heptosyltransferase I	GO:0009244, GO:0008920	No Seed vs T0	-7.89	4.56E-05
				Root vs T0	-4.76	1.98E-03
				Shoot vs T0	-4.47	5.79E-03
				FolRoot vs T0	-6.53	4.79E-04
				FolShoot vs T0	-4.15	8.07E-03
MMB18_RS04860	NA	hypothetical protein	NA	No Seed vs T0	-5.34	6.92E-09
				Root vs T0	-6.09	1.06E-09
				Shoot vs T0	-6.78	2.04E-09
				FolRoot vs T0	-7.46	6.08E-08
				FolShoot vs T0	-4.49	1.09E-06
MMB18_RS04870	hemW	radical SAM family heme chaperone HemW	GO:0006779, GO:0051539, GO:0051989, GO:0005737	No Seed vs T0	-3.59	1.38E-07
				Root vs T0	-3.37	7.70E-07
				Shoot vs T0	-4.23	8.89E-09
				FolRoot vs T0	-3.15	7.17E-06
				FolShoot vs T0	-3.12	6.59E-06
MMB18_RS04875	rdgB	RdgB/HAM1 family non-canonical purine NTP pyrophosphatase	GO:0006281, GO:0035870, GO:0036222	No Seed vs T0	-5.66	8.52E-05
				Root vs T0	-7.11	4.10E-06
				Shoot vs T0	-7.53	4.36E-06
				FolRoot vs T0	-6.03	7.98E-05
				FolShoot vs T0	-7.82	3.52E-06
MMB18_RS04920	greB	transcription elongation factor GreB	GO:0032784, GO:0003677, GO:0070063	No Seed vs T0	-3.25	9.50E-05
				Root vs T0	-3.43	7.16E-05
				Shoot vs T0	-3.71	8.35E-05
				FolRoot vs T0	-5.09	1.07E-04
				FolShoot vs T0	-4.86	2.37E-05
MMB18_RS04925	NA	porin	GO:0015288, GO:0009279	No Seed vs T0	-4.12	1.26E-05
				Root vs T0	-3.22	6.30E-04
				Shoot vs T0	-3.28	9.90E-04
				FolRoot vs T0	-2.89	3.05E-03
				FolShoot vs T0	-4.41	5.66E-06
MMB18_RS04940	NA	exonuclease domain-containing protein	GO:0004527	No Seed vs T0	-3.35	1.03E-05
				Root vs T0	-2.72	2.95E-04
				Shoot vs T0	-3.55	2.20E-05
				FolRoot vs T0	-2.46	1.63E-03
				FolShoot vs T0	-2.52	1.11E-03

MMB18_RS05005	pepN	aminopeptidase N	GO:0006508, GO:0004177, GO:0008237, GO:0008270	No Seed vs T0	-4.12	3.70E-12
				Root vs T0	-5.14	2.14E-17
				Shoot vs T0	-4.5	2.30E-12
				FolRoot vs T0	-2.64	1.55E-05
				FolShoot vs T0	-4.91	3.41E-15
MMB18_RS05020	guaD	guanine deaminase	GO:0006147, GO:0008270, GO:0008892	No Seed vs T0	-3.87	1.03E-07
				Root vs T0	-5.46	3.95E-12
				Shoot vs T0	-4.23	1.14E-07
				FolRoot vs T0	-4.65	2.64E-09
				FolShoot vs T0	-4.1	7.12E-08
MMB18_RS05025	NA	adenosine deaminase	GO:0006146, GO:0000034	No Seed vs T0	-3.72	1.72E-04
				Root vs T0	-3.8	1.39E-04
				Shoot vs T0	-4.93	1.11E-05
				FolRoot vs T0	-3.39	9.73E-04
				FolShoot vs T0	-4.05	1.04E-04
MMB18_RS05030	xdhC	xanthine dehydrogenase accessory protein XdhC	GO:0009115, GO:0065003, GO:0043546	No Seed vs T0	-6.32	2.08E-10
				Root vs T0	-5.72	1.17E-09
				Shoot vs T0	-5.92	6.47E-09
				FolRoot vs T0	-5.19	5.59E-08
				FolShoot vs T0	-6.03	5.39E-09
MMB18_RS05050	NA	gamma-glutamyl-gamma-aminobutyrate hydrolase family protein	GO:0016787, GO:0016811	No Seed vs T0	-4.48	2.29E-05
				Root vs T0	-4.86	5.24E-06
				Shoot vs T0	-5.36	4.62E-06
				FolRoot vs T0	-5.19	3.79E-06
				FolShoot vs T0	-4.53	3.19E-05
MMB18_RS05100	NA	substrate-binding domain-containing protein	NA	No Seed vs T0	-3.04	1.98E-11
				Root vs T0	-2.99	4.44E-11
				Shoot vs T0	-3.32	3.73E-11
				FolRoot vs T0	-2.36	3.53E-07
				FolShoot vs T0	-3.1	8.78E-11
MMB18_RS05135	gyrA	DNA gyrase subunit A	GO:0006259, GO:0006265, GO:0003677, GO:0003916, GO:0003918, GO:0005524	No Seed vs T0	-3.17	6.96E-04
				Root vs T0	-3.99	7.67E-05
				Shoot vs T0	-3.59	5.85E-04
				FolRoot vs T0	-2.96	2.84E-03
				FolShoot vs T0	-2.83	3.52E-03
MMB18_RS05145	serC	3-phosphoserine/phosphohydroxythreonine transaminase	GO:0006564, GO:0003824, GO:0004648	No Seed vs T0	-3.02	4.92E-04
				Root vs T0	-3.69	2.32E-05
				Shoot vs T0	-4.75	1.06E-06
				FolRoot vs T0	-4.21	4.38E-06
				FolShoot vs T0	-3.68	3.87E-05
MMB18_RS05160	aroA	3-phosphoshikimate 1-carboxyvinyltransferase	GO:0009073, GO:0003866	No Seed vs T0	-6.14	1.47E-05
				Root vs T0	-5.12	1.43E-04
				Shoot vs T0	-4.64	1.14E-03
				FolRoot vs T0	-5.7	1.08E-04
				FolShoot vs T0	-5.9	6.41E-05
MMB18_RS05220	NA	histone deacetylase family protein	NA	No Seed vs T0	-4.2	3.14E-06
				Root vs T0	-5.08	4.85E-08
				Shoot vs T0	-4.58	3.72E-06
				FolRoot vs T0	-6.53	7.75E-09
				FolShoot vs T0	-5.06	1.99E-07
MMB18_RS05235	NA	methionine ABC transporter permease	GO:0048473, GO:0033232, GO:0042626	No Seed vs T0	-6.83	3.34E-13
				Root vs T0	-6.79	4.94E-13
				Shoot vs T0	-7.79	4.16E-13
				FolRoot vs T0	-5.1	2.54E-08
				FolShoot vs T0	-6.66	8.61E-12
MMB18_RS05265	NA	PA0069 family radical SAM protein	GO:0003824, GO:0051536	No Seed vs T0	-6.7	5.57E-10
				Root vs T0	-6.05	7.75E-09
				Shoot vs T0	-5.32	1.56E-06
				FolRoot vs T0	-4.48	1.86E-05
				FolShoot vs T0	-8.92	5.20E-10
MMB18_RS05275	NA	PQQ-dependent sugar dehydrogenase	GO:0016901, GO:0070968	No Seed vs T0	-5.94	6.75E-05
				Root vs T0	-5.88	8.83E-05
				Shoot vs T0	-3.86	7.49E-03
				FolRoot vs T0	-3.63	9.44E-03
				FolShoot vs T0	-4.73	1.07E-03
MMB18_RS05290	trmD	tRNA (guanosine(37)-N1)-methyltransferase TrmD	GO:0008033, GO:0052906	No Seed vs T0	-7.12	3.27E-06
				Root vs T0	-5.93	4.12E-06
				Shoot vs T0	-5.69	1.07E-05
				FolRoot vs T0	-3.25	3.95E-03
				FolShoot vs T0	-5.07	4.29E-05
MMB18_RS05340	pmbA	metalloprotease PmbA	GO:0006508, GO:0008237	No Seed vs T0	-4.86	6.77E-13

				Root vs T0	-2.04	1.97E-03
				Shoot vs T0	-3.53	5.78E-07
				FolRoot vs T0	-2.32	6.01E-04
				FolShoot vs T0	-5.62	5.95E-14
MMB18_RS05430	NA	CreA family protein	NA	No Seed vs T0	-5.74	4.95E-08
				Root vs T0	-4.76	1.11E-06
				Shoot vs T0	-3.73	2.31E-04
				FolRoot vs T0	-3.69	1.54E-04
				FolShoot vs T0	-5.64	4.91E-07
MMB18_RS05480	NA	hypothetical protein	NA	No Seed vs T0	-7.83	1.45E-04
				Root vs T0	-6.69	5.21E-04
				Shoot vs T0	-4.74	8.27E-03
				FolRoot vs T0	-6.03	1.94E-03
				FolShoot vs T0	-8.17	7.56E-05
MMB18_RS05530	NA	Maf-like protein	NA	No Seed vs T0	-6.02	2.85E-06
				Root vs T0	-10.3	4.56E-09
				Shoot vs T0	-5.8	2.29E-05
				FolRoot vs T0	-6.23	6.13E-06
				FolShoot vs T0	-6.31	4.30E-06
MMB18_RS05640	NA	RNA-guided endonuclease InsQ/TnpB family protein	GO:0006310, GO:0032196, GO:0003677, GO:0004519, GO:0046872	No Seed vs T0	-5.43	3.48E-03
				Root vs T0	-6.56	4.21E-04
				Shoot vs T0	-6.57	8.94E-04
				FolRoot vs T0	-6.15	1.32E-03
				FolShoot vs T0	-6.39	7.21E-04
MMB18_RS05645	nagZ	beta-N-acetylhexosaminidase	GO:0005975, GO:0004553	No Seed vs T0	-5.53	4.18E-06
				Root vs T0	-4.12	4.47E-04
				Shoot vs T0	-4.08	1.11E-03
				FolRoot vs T0	-3.19	8.72E-03
				FolShoot vs T0	-3.69	2.00E-03
MMB18_RS05650	NA	sigma-54-dependent transcriptional regulator	GO:0000160, GO:0006355, GO:0000156, GO:0003677, GO:0005524	No Seed vs T0	-5.19	1.38E-06
				Root vs T0	-5.15	1.68E-06
				Shoot vs T0	-4.8	2.90E-05
				FolRoot vs T0	-4.44	5.52E-05
				FolShoot vs T0	-5.15	3.53E-06
MMB18_RS05665	uvrC	excinuclease ABC subunit UvrC	GO:0006289, GO:0009381, GO:0009380	No Seed vs T0	-2.87	9.51E-18
				Root vs T0	-3.15	1.72E-20
				Shoot vs T0	-3.22	2.92E-18
				FolRoot vs T0	-3.02	1.04E-17
				FolShoot vs T0	-2.79	1.60E-15
MMB18_RS05705	NA	GNAT family N-acetyltransferase	NA	No Seed vs T0	-5.19	2.10E-06
				Root vs T0	-6.47	1.09E-07
				Shoot vs T0	-5.02	1.91E-05
				FolRoot vs T0	-8.27	7.72E-07
				FolShoot vs T0	-8.19	1.71E-07
MMB18_RS05725	NA	GAF domain-containing protein	GO:0005515	No Seed vs T0	-5.61	4.90E-08
				Root vs T0	-7.12	3.32E-07
				Shoot vs T0	-3.92	5.35E-05
				FolRoot vs T0	-3.85	4.20E-05
				FolShoot vs T0	-3.84	3.62E-05
MMB18_RS05740	NA	GNAT family N-acetyltransferase	GO:0008080, GO:0016746	No Seed vs T0	-5.03	3.19E-04
				Root vs T0	-7.7	2.33E-05
				Shoot vs T0	-4.74	1.30E-03
				FolRoot vs T0	-4.01	4.73E-03
				FolShoot vs T0	-3.82	6.13E-03
MMB18_RS05785	NA	DUF2214 family protein	NA	No Seed vs T0	-4.72	4.28E-15
				Root vs T0	-4.29	2.77E-13
				Shoot vs T0	-3.91	4.06E-10
				FolRoot vs T0	-4.47	1.23E-12
				FolShoot vs T0	-4.08	1.81E-11
MMB18_RS05815	NA	alpha/beta fold hydrolase	NA	No Seed vs T0	-6.69	4.28E-15
				Root vs T0	-5.28	3.08E-14
				Shoot vs T0	-3.84	1.21E-08
				FolRoot vs T0	-4.39	8.40E-11
				FolShoot vs T0	-8.57	1.17E-10
MMB18_RS05900	aruF	arginine/ornithine succinyltransferase subunit alpha	NA	No Seed vs T0	-6.66	1.16E-05
				Root vs T0	-4.18	8.98E-04
				Shoot vs T0	-3.91	3.70E-03
				FolRoot vs T0	-7.42	3.56E-05
				FolShoot vs T0	-4.74	4.23E-04
MMB18_RS05905	astA	arginine N-succinyltransferase	GO:0006527, GO:0008791	No Seed vs T0	-3.57	2.34E-03
				Root vs T0	-6.03	1.04E-05

				Shoot vs T0	-4.35	7.92E-04
				FolRoot vs T0	-5.28	1.07E-04
				FolShoot vs T0	-5.35	7.43E-05
MMB18_RS05950	NA	NYN domain-containing protein	GO:0004540	No Seed vs T0	-4.66	8.16E-07
				Root vs T0	-3.87	3.58E-05
				Shoot vs T0	-3.06	1.99E-03
				FolRoot vs T0	-3.66	1.49E-04
				FolShoot vs T0	-2.71	4.34E-03
MMB18_RS05960	NA	efflux RND transporter permease subunit	GO:0055085, GO:0022857, GO:0016020	No Seed vs T0	-5.24	4.78E-08
				Root vs T0	-5.76	3.18E-09
				Shoot vs T0	-5.33	2.83E-07
				FolRoot vs T0	-3.4	5.37E-04
				FolShoot vs T0	-4.3	9.77E-06
MMB18_RS06170	NA	amino acid permease	GO:0006865, GO:0055085, GO:0015171, GO:0016020	No Seed vs T0	-5.57	2.33E-10
				Root vs T0	-4.48	1.26E-08
				Shoot vs T0	-3.31	3.58E-05
				FolRoot vs T0	-2.74	3.00E-04
				FolShoot vs T0	-3.23	2.49E-05
MMB18_RS06255	NA	MFS transporter	GO:0055085, GO:0022857, GO:0005886, GO:0016020	No Seed vs T0	-5	3.79E-05
				Root vs T0	-4.94	4.90E-05
				Shoot vs T0	-4.43	6.47E-04
				FolRoot vs T0	-4.16	7.77E-04
				FolShoot vs T0	-3.25	7.18E-03
MMB18_RS06410	NA	DUF2242 domain-containing protein	NA	No Seed vs T0	-7.26	6.53E-08
				Root vs T0	-4.68	9.43E-05
				Shoot vs T0	-4.71	2.59E-04
				FolRoot vs T0	-4.28	5.34E-04
				FolShoot vs T0	-5.81	5.94E-06
MMB18_RS06440	NA	3-hydroxyacyl-CoA dehydrogenase NAD-binding domain-containing protein	GO:0006631, GO:0070403	No Seed vs T0	-4.53	1.65E-06
				Root vs T0	-7.49	1.47E-07
				Shoot vs T0	-3.07	1.70E-03
				FolRoot vs T0	-3.8	8.33E-05
				FolShoot vs T0	-3.56	1.70E-04
MMB18_RS06645	phoR	phosphate regulon sensor histidine kinase PhoR	GO:0000160, GO:0006796, GO:0006817, GO:0000155	No Seed vs T0	-3.04	1.74E-04
				Root vs T0	-3.72	7.55E-06
				Shoot vs T0	-3.21	3.06E-04
				FolRoot vs T0	-2.94	5.35E-04
				FolShoot vs T0	-3.7	1.92E-05
MMB18_RS06660	NA	hypothetical protein	NA	No Seed vs T0	-4.05	4.85E-06
				Root vs T0	-3.8	1.65E-05
				Shoot vs T0	-3.5	2.31E-04
				FolRoot vs T0	-3.62	8.08E-05
				FolShoot vs T0	-3.67	5.61E-05
MMB18_RS06690	NA	MATE family efflux transporter	GO:0006855, GO:0042910, GO:0016020	No Seed vs T0	-6.24	4.06E-09
				Root vs T0	-5.04	2.80E-07
				Shoot vs T0	-4.18	4.79E-05
				FolRoot vs T0	-6.41	5.41E-08
				FolShoot vs T0	-5.5	2.10E-07
MMB18_RS06710	NA	aldose epimerase	NA	No Seed vs T0	-3.74	3.49E-03
				Root vs T0	-4.13	1.51E-03
				Shoot vs T0	-3.87	5.19E-03
				FolRoot vs T0	-4.28	2.00E-03
				FolShoot vs T0	-3.87	3.94E-03
MMB18_RS06715	NA	undecaprenyl-diphosphate phosphatase	NA	No Seed vs T0	-5.6	5.92E-05
				Root vs T0	-7.55	1.72E-05
				Shoot vs T0	-6.28	4.41E-05
				FolRoot vs T0	-3.57	6.54E-03
				FolShoot vs T0	-5.2	2.81E-04
MMB18_RS06725	NA	hypothetical protein	NA	No Seed vs T0	-4.89	2.90E-10
				Root vs T0	-4.87	3.41E-10
				Shoot vs T0	-5.81	7.25E-11
				FolRoot vs T0	-4.14	1.40E-07
				FolShoot vs T0	-4.45	2.22E-08
MMB18_RS06750	NA	acyl-CoA dehydrogenase	GO:0006631, GO:0003995, GO:0050660	No Seed vs T0	-5.77	4.00E-09
				Root vs T0	-5.87	2.77E-09
				Shoot vs T0	-5.51	1.84E-07
				FolRoot vs T0	-4.52	4.38E-06
				FolShoot vs T0	-8.67	4.16E-10
MMB18_RS06755	NA	NADPH:quinone oxidoreductase family protein	GO:0016491, GO:0016655, GO:0048038, GO:0070402	No Seed vs T0	-2.7	1.21E-04
				Root vs T0	-3.48	1.45E-06
				Shoot vs T0	-3.71	3.16E-06

				FolRoot vs T0	-3.51	4.38E-06
				FolShoot vs T0	-2.63	3.08E-04
MMB18_RS06945	NA	bifunctional alpha/beta hydrolase/class I SAM-dependent methyltransferase	GO:0008168, GO:0016787, GO:1904047	No Seed vs T0	-3.39	3.10E-09
				Root vs T0	-3.67	1.80E-10
				Shoot vs T0	-4.24	3.20E-11
				FolRoot vs T0	-3.82	1.94E-10
				FolShoot vs T0	-4.43	8.91E-13
MMB18_RS06980	bcsE	cellulose biosynthesis protein BcsE	GO:0035438	No Seed vs T0	-3.61	5.43E-05
				Root vs T0	-2.99	8.29E-04
				Shoot vs T0	-3.31	5.62E-04
				FolRoot vs T0	-2.99	1.21E-03
				FolShoot vs T0	-4.08	1.26E-05
MMB18_RS07125	NA	NUDIX domain-containing protein	GO:0016787	No Seed vs T0	-3.46	4.99E-04
				Root vs T0	-3.46	5.45E-04
				Shoot vs T0	-5.58	1.70E-05
				FolRoot vs T0	-5	1.33E-04
				FolShoot vs T0	-5.68	2.81E-05
MMB18_RS07145	NA	ABC transporter ATP-binding protein	GO:0005524, GO:0016887, GO:0042626, GO:0140359	No Seed vs T0	-3.28	1.06E-04
				Root vs T0	-5.57	1.23E-07
				Shoot vs T0	-3.25	4.48E-04
				FolRoot vs T0	-2.55	3.50E-03
				FolShoot vs T0	-4.16	1.04E-05
MMB18_RS07255	NA	CHASE2 domain-containing protein	NA	No Seed vs T0	-3.8	1.35E-08
				Root vs T0	-3.24	1.04E-06
				Shoot vs T0	-3.22	6.40E-06
				FolRoot vs T0	-4.1	5.66E-09
				FolShoot vs T0	-4.62	1.63E-10
MMB18_RS07305	NA	FUSC family protein	GO:0055085, GO:0022857, GO:0005886	No Seed vs T0	-3.28	5.03E-09
				Root vs T0	-3.27	5.93E-09
				Shoot vs T0	-3.04	4.89E-07
				FolRoot vs T0	-2.85	8.45E-07
				FolShoot vs T0	-3.08	9.64E-08
MMB18_RS07345	NA	YihY family inner membrane protein	GO:0005886	No Seed vs T0	-5.55	6.72E-05
				Root vs T0	-7.26	9.81E-07
				Shoot vs T0	-6.53	1.73E-05
				FolRoot vs T0	-5.11	3.76E-04
				FolShoot vs T0	-5.21	2.49E-04
MMB18_RS07365	aroC	chorismate synthase	GO:0009073, GO:0004107	No Seed vs T0	-6.4	3.98E-17
				Root vs T0	-6.21	1.62E-16
				Shoot vs T0	-4.6	3.71E-11
				FolRoot vs T0	-4.76	2.90E-12
				FolShoot vs T0	-5.89	1.63E-14
MMB18_RS07420	NA	RelA/SpoT family protein	GO:0015970, GO:0008728, GO:0008893	No Seed vs T0	-6.57	2.45E-09
				Root vs T0	-4.6	2.59E-05
				Shoot vs T0	-5.14	6.87E-06
				FolRoot vs T0	-6.04	7.50E-08
				FolShoot vs T0	-3.68	8.99E-04
MMB18_RS07515	NA	YdcF family protein	GO:1904047	No Seed vs T0	-7.86	1.71E-07
				Root vs T0	-6.82	3.69E-07
				Shoot vs T0	-5.05	6.12E-07
				FolRoot vs T0	-5.76	1.43E-06
				FolShoot vs T0	-7.23	3.79E-07
MMB18_RS07530	NA	transposase	GO:0006310, GO:0015074, GO:0032196, GO:0003677, GO:0004803	No Seed vs T0	-2.47	2.11E-03
				Root vs T0	-2.08	9.94E-03
				Shoot vs T0	-3.46	8.88E-05
				FolRoot vs T0	-3.67	1.80E-05
				FolShoot vs T0	-2.88	5.15E-04
MMB18_RS07605	typA	translational GTPase TypA	GO:0006412, GO:0006950, GO:0003924, GO:0005525	No Seed vs T0	-5.02	2.34E-06
				Root vs T0	-8.42	3.39E-13
				Shoot vs T0	-7	2.15E-09
				FolRoot vs T0	-4.49	3.90E-05
				FolShoot vs T0	-8.83	2.58E-12
MMB18_RS07610	NA	2-oxoglutarate dehydrogenase E1 component	GO:0006099, GO:0004591, GO:0030976	No Seed vs T0	-2.44	1.92E-03
				Root vs T0	-5.19	2.56E-08
				Shoot vs T0	-2.98	6.02E-04
				FolRoot vs T0	-3.12	2.08E-04
				FolShoot vs T0	-5.02	2.34E-07
MMB18_RS07715	NA	sigma 54-interacting transcriptional regulator	GO:0006355, GO:0005524, GO:0008134	No Seed vs T0	-3.92	6.59E-05
				Root vs T0	-4.23	2.44E-05
				Shoot vs T0	-3.51	9.50E-04
				FolRoot vs T0	-3.66	3.75E-04

				FolShoot vs T0	-3.99	1.18E-04
MMB18_RS07905	NA	amino acid ABC transporter permease	GO:0006865, GO:0042626, GO:0140359	No Seed vs T0	-2.56	3.28E-04
				Root vs T0	-3.19	9.91E-06
				Shoot vs T0	-3.42	1.25E-05
				FolRoot vs T0	-3.12	3.18E-05
				FolShoot vs T0	-3.7	1.08E-06
MMB18_RS07925	aat	leucyl/phenylalanyl-tRNA--protein transferase	GO:0006508, GO:0008914	No Seed vs T0	-9.27	2.93E-06
				Root vs T0	-5.35	9.52E-04
				Shoot vs T0	-5.62	1.10E-03
				FolRoot vs T0	-7.73	1.54E-04
				FolShoot vs T0	-4.75	3.89E-03
MMB18_RS08040	NA	sensor domain-containing diguanylate cyclase	NA	No Seed vs T0	-4.88	1.82E-07
				Root vs T0	-6.06	3.01E-09
				Shoot vs T0	-6.27	1.20E-08
				FolRoot vs T0	-4.46	4.02E-06
				FolShoot vs T0	-6.71	9.67E-09
MMB18_RS08050	NA	phospholipase D family protein	NA	No Seed vs T0	-6.18	5.42E-06
				Root vs T0	-4.94	5.41E-05
				Shoot vs T0	-6.16	1.12E-05
				FolRoot vs T0	-6.1	4.15E-05
				FolShoot vs T0	-3.6	2.64E-03
MMB18_RS08160	NA	DUF805 domain-containing protein	GO:0016020	No Seed vs T0	-7.27	2.11E-06
				Root vs T0	-4.94	3.95E-06
				Shoot vs T0	-4.91	8.58E-06
				FolRoot vs T0	-3.43	4.24E-04
				FolShoot vs T0	-5.58	6.36E-06
MMB18_RS08180	NA	LacI family DNA-binding transcriptional regulator	GO:0006355, GO:0003677, GO:0003700	No Seed vs T0	-6.94	2.23E-06
				Root vs T0	-3.6	6.13E-03
				Shoot vs T0	-4.83	7.69E-04
				FolRoot vs T0	-4.83	4.81E-04
				FolShoot vs T0	-6.51	1.50E-05
MMB18_RS08195	NA	PrkA family serine protein kinase	GO:0004672	No Seed vs T0	-5.61	6.72E-09
				Root vs T0	-5.25	3.62E-08
				Shoot vs T0	-3.23	9.02E-04
				FolRoot vs T0	-3.5	2.16E-04
				FolShoot vs T0	-4.93	4.72E-07
MMB18_RS08210	NA	MFS family transporter	GO:0016740	No Seed vs T0	-6.94	2.89E-04
				Root vs T0	-5.16	6.23E-03
				Shoot vs T0	-5.99	2.76E-03
				FolRoot vs T0	-5.52	4.68E-03
				FolShoot vs T0	-5.42	4.70E-03
MMB18_RS08360	NA	ABC transporter ATP-binding protein	GO:0005524, GO:0016887, GO:0042626, GO:0140359	No Seed vs T0	-5.15	1.36E-05
				Root vs T0	-5.61	3.54E-06
				Shoot vs T0	-4.76	1.83E-04
				FolRoot vs T0	-3.53	3.25E-03
				FolShoot vs T0	-5.82	5.56E-06
MMB18_RS08390	NA	non-ribosomal peptide synthetase	NA	No Seed vs T0	-4.08	6.38E-18
				Root vs T0	-3.06	3.58E-11
				Shoot vs T0	-2.67	5.78E-08
				FolRoot vs T0	-3.02	2.00E-10
				FolShoot vs T0	-3.04	1.75E-10
MMB18_RS08495	cobN	cobaltochelatase subunit CobN	GO:0009236, GO:0051116	No Seed vs T0	-3.23	2.97E-09
				Root vs T0	-3.47	1.93E-10
				Shoot vs T0	-3.4	4.62E-09
				FolRoot vs T0	-3.12	2.27E-08
				FolShoot vs T0	-3.36	1.72E-09
MMB18_RS08515	NA	DUF1993 domain-containing protein	NA	No Seed vs T0	-3.36	1.85E-06
				Root vs T0	-2.59	1.77E-04
				Shoot vs T0	-3.41	1.02E-05
				FolRoot vs T0	-2.15	2.64E-03
				FolShoot vs T0	-3.02	3.30E-05
MMB18_RS08545	NA	IclR family transcriptional regulator	GO:0006355, GO:0003677, GO:0003700	No Seed vs T0	-4.4	2.39E-04
				Root vs T0	-6	6.17E-06
				Shoot vs T0	-3.93	2.25E-03
				FolRoot vs T0	-3.65	3.11E-03
				FolShoot vs T0	-3.7	2.41E-03
MMB18_RS08590	NA	cobalt-precorrin-5B (C(1))-methyltransferase	GO:0009236, GO:0008168	No Seed vs T0	-4.14	5.67E-13
				Root vs T0	-3.95	4.80E-12
				Shoot vs T0	-4.01	1.33E-10
				FolRoot vs T0	-3.99	3.32E-11
				FolShoot vs T0	-5.46	4.23E-15

MMB18_RS08750	NA	5-formyltetrahydrofolate cyclo-ligase	NA	No Seed vs T0	-4.02	7.71E-08
				Root vs T0	-4.77	3.40E-08
				Shoot vs T0	-2.78	1.29E-04
				FolRoot vs T0	-4.34	9.97E-07
				FolShoot vs T0	-3.05	2.34E-05
MMB18_RS09095	NA	DUF3022 domain-containing protein	NA	No Seed vs T0	-3.2	1.14E-06
				Root vs T0	-2.82	1.34E-05
				Shoot vs T0	-3.79	5.11E-07
				FolRoot vs T0	-3.2	5.83E-06
				FolShoot vs T0	-4.1	7.91E-08
MMB18_RS09150	NA	efflux RND transporter periplasmic adaptor subunit	GO:0055085, GO:0022857, GO:0042802, GO:0016020	No Seed vs T0	-7.09	1.34E-04
				Root vs T0	-6.63	3.19E-04
				Shoot vs T0	-5.18	6.76E-03
				FolRoot vs T0	-5.02	7.55E-03
				FolShoot vs T0	-5.44	3.26E-03
MMB18_RS09155	NA	efflux RND transporter permease subunit	GO:0042908, GO:0015562, GO:0022857, GO:0016020	No Seed vs T0	-2.68	2.14E-05
				Root vs T0	-4.05	1.74E-10
				Shoot vs T0	-3.28	1.05E-06
				FolRoot vs T0	-3.3	3.57E-07
				FolShoot vs T0	-3.34	2.10E-07
MMB18_RS09200	NA	bifunctional protein tyrosine phosphatase family protein/NAD(P)/FAD-dependent oxidoreductase	GO:0016787	No Seed vs T0	-3.74	1.45E-04
				Root vs T0	-4.62	3.22E-06
				Shoot vs T0	-5.48	5.32E-07
				FolRoot vs T0	-2.89	4.75E-03
				FolShoot vs T0	-6.57	3.66E-09
MMB18_RS09245	NA	aldehyde dehydrogenase family protein	NA	No Seed vs T0	-4.35	1.23E-05
				Root vs T0	-4.84	1.27E-06
				Shoot vs T0	-4.45	2.71E-05
				FolRoot vs T0	-3.45	7.52E-04
				FolShoot vs T0	-4.27	2.55E-05
MMB18_RS09295	NA	4Fe-4S cluster-binding domain-containing protein	NA	No Seed vs T0	-2.11	4.56E-03
				Root vs T0	-2.38	1.56E-03
				Shoot vs T0	-2.18	7.26E-03
				FolRoot vs T0	-3.66	3.07E-05
				FolShoot vs T0	-2.8	4.84E-04
MMB18_RS09310	NA	IS5-like element ISBmu23 family transposase	GO:0006313, GO:0004803	No Seed vs T0	-4.92	1.02E-06
				Root vs T0	-5.6	4.33E-08
				Shoot vs T0	-5.04	3.32E-06
				FolRoot vs T0	-3.7	3.44E-04
				FolShoot vs T0	-3.84	1.62E-04
MMB18_RS09320	NA	hypothetical protein	NA	No Seed vs T0	-10.3	5.65E-07
				Root vs T0	-7.23	2.06E-05
				Shoot vs T0	-8.55	4.34E-06
				FolRoot vs T0	-7.69	2.27E-05
				FolShoot vs T0	-5.71	6.99E-04
MMB18_RS09335	NA	AsmA family protein	NA	No Seed vs T0	-6.38	5.80E-08
				Root vs T0	-6.45	4.95E-08
				Shoot vs T0	-4.49	2.23E-04
				FolRoot vs T0	-4.44	1.69E-04
				FolShoot vs T0	-6.9	4.69E-08
MMB18_RS09365	NA	LysR family transcriptional regulator	GO:0006355, GO:0001216, GO:0032993	No Seed vs T0	-4.92	1.63E-05
				Root vs T0	-4.84	2.35E-05
				Shoot vs T0	-3.74	9.09E-04
				FolRoot vs T0	-4.76	1.13E-04
				FolShoot vs T0	-4.55	1.12E-04
MMB18_RS09415	NA	lysozyme inhibitor LprI family protein	NA	No Seed vs T0	-6.72	8.37E-06
				Root vs T0	-5.66	8.64E-05
				Shoot vs T0	-5.32	5.25E-04
				FolRoot vs T0	-6.72	3.12E-05
				FolShoot vs T0	-5.03	5.81E-04
MMB18_RS09490	NA	ATP phosphoribosyltransferase regulatory subunit	GO:0000105	No Seed vs T0	-8.77	2.13E-08
				Root vs T0	-7.89	5.14E-08
				Shoot vs T0	-8.07	1.17E-07
				FolRoot vs T0	-6.65	2.55E-06
				FolShoot vs T0	-7.6	3.50E-07
MMB18_RS09570	NA	endonuclease/exonuclease/phosphatase family protein	GO:0003824	No Seed vs T0	-5.86	4.92E-07
				Root vs T0	-5.8	7.10E-07
				Shoot vs T0	-5.25	1.69E-05
				FolRoot vs T0	-5.16	1.39E-05
				FolShoot vs T0	-5.73	2.71E-06
MMB18_RS09660	NA	MATE family efflux transporter	GO:0006855, GO:0042910,	No Seed vs T0	-6.52	1.33E-07

			GO:0016020	Root vs T0	-6.27	2.95E-07
				Shoot vs T0	-6.41	8.46E-07
				FolRoot vs T0	-5.28	1.23E-05
				FolShoot vs T0	-4.72	5.15E-05
MMB18_RS09725	NA	Rrf2 family transcriptional regulator	NA	No Seed vs T0	-6.15	4.24E-04
				Root vs T0	-6.01	5.64E-04
				Shoot vs T0	-6.24	7.25E-04
				FolRoot vs T0	-5.07	4.30E-03
				FolShoot vs T0	-5.07	3.84E-03
MMB18_RS09885	lysM	peptidoglycan-binding protein LysM	NA	No Seed vs T0	-8.21	1.99E-07
				Root vs T0	-8.1	2.98E-07
				Shoot vs T0	-4.88	4.05E-05
				FolRoot vs T0	-5.21	8.16E-05
				FolShoot vs T0	-7.58	1.78E-06
MMB18_RS09890	NA	asparaginase	NA	No Seed vs T0	-3.09	2.43E-07
				Root vs T0	-3.61	5.93E-09
				Shoot vs T0	-2.97	5.12E-06
				FolRoot vs T0	-2.35	1.29E-04
				FolShoot vs T0	-3.24	3.68E-07
MMB18_RS09920	NA	mechanosensitive ion channel family protein	GO:0055085, GO:0008381, GO:0016020	No Seed vs T0	-7.64	3.72E-07
				Root vs T0	-4.52	5.00E-04
				Shoot vs T0	-4.39	1.55E-03
				FolRoot vs T0	-4.67	5.37E-04
				FolShoot vs T0	-6.15	1.35E-05
MMB18_RS09970	NA	alpha/beta hydrolase	GO:0016787	No Seed vs T0	-5.56	6.20E-05
				Root vs T0	-5.01	2.19E-04
				Shoot vs T0	-8.12	4.76E-06
				FolRoot vs T0	-6.33	9.56E-05
				FolShoot vs T0	-5.09	3.44E-04
MMB18_RS09980	NA	Dabb family protein	NA	No Seed vs T0	-3.48	5.49E-03
				Root vs T0	-4.99	4.05E-04
				Shoot vs T0	-3.99	3.62E-03
				FolRoot vs T0	-7.32	3.67E-05
				FolShoot vs T0	-5.74	2.81E-04
MMB18_RS10040	NA	acetyl-CoA C-acyltransferase	GO:0016747	No Seed vs T0	-8.07	4.27E-11
				Root vs T0	-6.35	3.42E-15
				Shoot vs T0	-4.36	8.44E-12
				FolRoot vs T0	-4.85	5.42E-13
				FolShoot vs T0	-4.84	2.20E-13
MMB18_RS10145	clpP	ATP-dependent Clp endopeptidase proteolytic subunit ClpP	GO:0006508, GO:0004176, GO:0004252, GO:0009368	No Seed vs T0	-8.25	9.74E-09
				Root vs T0	-5.29	2.95E-10
				Shoot vs T0	-5.09	3.57E-09
				FolRoot vs T0	-3.17	1.25E-05
				FolShoot vs T0	-8.59	2.48E-09
MMB18_RS10165	NA	2-dehydropantoate 2-reductase	GO:0015940, GO:0008677, GO:0050661	No Seed vs T0	-4.19	9.74E-07
				Root vs T0	-3.81	4.54E-06
				Shoot vs T0	-2.45	2.98E-03
				FolRoot vs T0	-2.58	1.39E-03
				FolShoot vs T0	-2.53	1.43E-03
MMB18_RS10210	NA	DUF1501 domain-containing protein	NA	No Seed vs T0	-4.91	1.80E-06
				Root vs T0	-5.14	1.23E-06
				Shoot vs T0	-3.52	7.34E-04
				FolRoot vs T0	-3.12	1.65E-03
				FolShoot vs T0	-4.3	3.61E-05
MMB18_RS10215	NA	DUF1800 domain-containing protein	NA	No Seed vs T0	-4.19	4.19E-05
				Root vs T0	-3.62	3.76E-04
				Shoot vs T0	-5.81	6.06E-07
				FolRoot vs T0	-4.16	8.96E-05
				FolShoot vs T0	-3.5	7.70E-04
MMB18_RS10290	mscL	large conductance mechanosensitive channel protein MscL	GO:0034220, GO:0008381, GO:0016020	No Seed vs T0	-3.4	3.37E-04
				Root vs T0	-5.14	3.76E-07
				Shoot vs T0	-2.99	3.22E-03
				FolRoot vs T0	-3.87	1.11E-04
				FolShoot vs T0	-3.11	1.38E-03
MMB18_RS10310	NA	LysR substrate-binding domain-containing protein	NA	No Seed vs T0	-6.36	1.09E-04
				Root vs T0	-6.09	1.83E-04
				Shoot vs T0	-5.8	5.83E-04
				FolRoot vs T0	-4.5	5.04E-03
				FolShoot vs T0	-4.9	2.21E-03
MMB18_RS10455	NA	LysR family transcriptional regulator	GO:0001216, GO:0003677, GO:0003700	No Seed vs T0	-6.81	8.65E-07
				Root vs T0	-4.02	2.23E-03

				Shoot vs T0	-5.05	3.62E-04
				FolRoot vs T0	-3.84	4.73E-03
				FolShoot vs T0	-5.21	1.21E-04
MMB18_RS10555	NA	pyruvate, water dikinase regulatory protein	GO:0006468, GO:0006470, GO:0004674, GO:0005524, GO:0016776	No Seed vs T0	-7.23	2.06E-04
				Root vs T0	-4.66	5.57E-03
				Shoot vs T0	-5.64	1.93E-03
				FolRoot vs T0	-5.7	1.98E-03
				FolShoot vs T0	-8.5	3.71E-05
MMB18_RS10600	rseP	RIP metalloprotease RseP	GO:0006508, GO:0004222, GO:0016020	No Seed vs T0	-8.17	4.44E-10
				Root vs T0	-4.93	1.50E-05
				Shoot vs T0	-4.32	3.16E-04
				FolRoot vs T0	-5.73	1.64E-06
				FolShoot vs T0	-7.73	8.19E-09
MMB18_RS10705	cls	cardiolipin synthase	GO:0032049, GO:0008808, GO:0016780	No Seed vs T0	-3.5	1.47E-04
				Root vs T0	-3.62	9.06E-05
				Shoot vs T0	-4.03	6.25E-05
				FolRoot vs T0	-3.07	1.28E-03
				FolShoot vs T0	-3.57	1.71E-04
MMB18_RS10775	aceA	isocitrate lyase	GO:0019752, GO:0004451	No Seed vs T0	-6.4	1.88E-10
				Root vs T0	-6.43	2.36E-10
				Shoot vs T0	-5.48	7.83E-08
				FolRoot vs T0	-4.54	1.59E-06
				FolShoot vs T0	-4.6	1.04E-06
MMB18_RS10800	NA	gamma-glutamylcyclotransferase family protein	GO:0061929	No Seed vs T0	-6.4	3.49E-06
				Root vs T0	-5.53	1.21E-05
				Shoot vs T0	-6.19	8.44E-06
				FolRoot vs T0	-7.47	1.55E-05
				FolShoot vs T0	-6.85	6.36E-06
MMB18_RS10810	NA	GNAT family N-acetyltransferase	GO:0016747	No Seed vs T0	-8.7	2.17E-06
				Root vs T0	-6.71	9.74E-05
				Shoot vs T0	-6.05	1.82E-04
				FolRoot vs T0	-6.36	3.20E-04
				FolShoot vs T0	-8.08	1.23E-05
MMB18_RS10855	NA	patatin-like phospholipase family protein	GO:0052689	No Seed vs T0	-8.76	1.67E-08
				Root vs T0	-7.67	8.95E-09
				Shoot vs T0	-6.46	3.40E-07
				FolRoot vs T0	-4.51	8.94E-05
				FolShoot vs T0	-6.99	9.92E-08
MMB18_RS10865	NA	ABC transporter ATP-binding protein	GO:0005524, GO:0016887, GO:0042626, GO:0140359	No Seed vs T0	-5.29	8.87E-06
				Root vs T0	-6.35	3.56E-07
				Shoot vs T0	-6.27	2.09E-06
				FolRoot vs T0	-4.92	6.13E-05
				FolShoot vs T0	-6.78	5.64E-07
MMB18_RS10870	NA	ABC transporter permease	NA	No Seed vs T0	-6.59	9.05E-06
				Root vs T0	-6.24	1.77E-05
				Shoot vs T0	-6.49	2.38E-05
				FolRoot vs T0	-4.35	1.84E-03
				FolShoot vs T0	-5.62	1.20E-04
MMB18_RS10880	NA	extracellular solute-binding protein	NA	No Seed vs T0	-2.85	5.65E-07
				Root vs T0	-3.52	8.62E-10
				Shoot vs T0	-3.5	1.66E-08
				FolRoot vs T0	-2.14	2.89E-04
				FolShoot vs T0	-3.12	1.14E-07
MMB18_RS10915	NA	sensor histidine kinase	GO:0000160, GO:0007165, GO:0000155, GO:0004673, GO:0005524, GO:0016020	No Seed vs T0	-5.02	1.38E-06
				Root vs T0	-5.23	8.68E-07
				Shoot vs T0	-4.41	5.76E-05
				FolRoot vs T0	-3.62	4.45E-04
				FolShoot vs T0	-4.93	6.59E-06
MMB18_RS10970	NA	AAA domain-containing protein	GO:0004386	No Seed vs T0	-5.62	3.35E-21
				Root vs T0	-5.45	3.41E-20
				Shoot vs T0	-5.05	9.88E-16
				FolRoot vs T0	-3.98	1.71E-11
				FolShoot vs T0	-5.31	6.79E-18
MMB18_RS10975	NA	hypothetical protein	NA	No Seed vs T0	-10.9	3.94E-05
				Root vs T0	-6.53	6.65E-03
				Shoot vs T0	-9.81	2.48E-04
				FolRoot vs T0	-7.42	3.52E-03
				FolShoot vs T0	-7.49	2.82E-03
MMB18_RS10990	NA	ankyrin repeat domain-containing protein	GO:0005515	No Seed vs T0	-6.39	1.91E-05
				Root vs T0	-7.64	2.05E-06
				Shoot vs T0	-5.26	7.95E-04

				FolRoot vs T0	-6.56	3.42E-05
				FolShoot vs T0	-8.08	2.47E-06
MMB18_RS10995	NA	PAAR-like domain-containing protein	NA	No Seed vs T0	-10.2	2.55E-05
				Root vs T0	-9.13	8.50E-05
				Shoot vs T0	-10.1	3.04E-05
				FolRoot vs T0	-6.38	3.44E-03
				FolShoot vs T0	-7.38	8.19E-04
MMB18_RS11000	NA	DUF2169 family type VI secretion system accessory protein	NA	No Seed vs T0	-6.38	1.41E-05
				Root vs T0	-6.78	5.53E-06
				Shoot vs T0	-6.68	2.30E-05
				FolRoot vs T0	-8.16	2.64E-06
				FolShoot vs T0	-8.61	6.17E-07
MMB18_RS11005	NA	type VI secretion system Vgr family protein	GO:0033103, GO:0033104	No Seed vs T0	-5.72	1.17E-09
				Root vs T0	-5.43	5.65E-09
				Shoot vs T0	-6.61	4.99E-10
				FolRoot vs T0	-3.89	3.53E-05
				FolShoot vs T0	-6	1.79E-09
MMB18_RS11010	tssF	type VI secretion system baseplate subunit TssF	GO:0033103	No Seed vs T0	-5.68	4.79E-06
				Root vs T0	-5.68	5.56E-06
				Shoot vs T0	-4.84	1.69E-04
				FolRoot vs T0	-4.92	9.65E-05
				FolShoot vs T0	-4.93	8.02E-05
MMB18_RS11020	NA	DUF3592 domain-containing protein	NA	No Seed vs T0	-9.12	1.31E-07
				Root vs T0	-9.01	1.89E-07
				Shoot vs T0	-5.51	6.80E-05
				FolRoot vs T0	-5.96	5.65E-05
				FolShoot vs T0	-5.92	3.46E-05
MMB18_RS11075	NA	hypothetical protein	NA	No Seed vs T0	-9.06	3.72E-08
				Root vs T0	-8	1.41E-06
				Shoot vs T0	-8.03	8.46E-07
				FolRoot vs T0	-6.17	1.66E-05
				FolShoot vs T0	-5.76	1.14E-05
MMB18_RS11095	NA	helix-turn-helix domain-containing protein	GO:0003677	No Seed vs T0	-5.25	3.23E-11
				Root vs T0	-4.22	1.68E-08
				Shoot vs T0	-4.21	2.39E-07
				FolRoot vs T0	-3.04	5.83E-05
				FolShoot vs T0	-4.2	7.39E-08
MMB18_RS11125	NA	hypothetical protein	NA	No Seed vs T0	-3.78	3.61E-03
				Root vs T0	-6.59	3.02E-07
				Shoot vs T0	-6.26	3.54E-06
				FolRoot vs T0	-6.33	1.59E-06
				FolShoot vs T0	-7.19	5.48E-08
MMB18_RS11130	NA	H-NS family nucleoid-associated regulatory protein	GO:0006355, GO:0003677, GO:0030527	No Seed vs T0	-6.95	4.34E-04
				Root vs T0	-8.52	1.09E-04
				Shoot vs T0	-8.51	1.05E-04
				FolRoot vs T0	-8.05	3.68E-04
				FolShoot vs T0	-8.97	4.71E-05
MMB18_RS11180	NA	helix-turn-helix transcriptional regulator	GO:0006355, GO:0003677, GO:0003700	No Seed vs T0	-9.61	1.28E-14
				Root vs T0	-5.96	1.65E-11
				Shoot vs T0	-6.56	2.12E-11
				FolRoot vs T0	-9.63	6.08E-12
				FolShoot vs T0	-9.25	6.58E-14
MMB18_RS11240	NA	RNA methyltransferase	NA	No Seed vs T0	-2.95	1.91E-06
				Root vs T0	-2.78	7.35E-06
				Shoot vs T0	-2.78	2.38E-05
				FolRoot vs T0	-2.75	1.76E-05
				FolShoot vs T0	-3.29	2.52E-07
MMB18_RS11270	NA	cupin domain-containing protein	NA	No Seed vs T0	-2.55	4.59E-06
				Root vs T0	-2.16	1.08E-04
				Shoot vs T0	-2.65	6.83E-06
				FolRoot vs T0	-2.11	2.43E-04
				FolShoot vs T0	-2.88	3.77E-07
MMB18_RS11310	NA	L-threonylcarbamoyladenylate synthase	GO:0006400, GO:0003725, GO:0061710	No Seed vs T0	-4.98	6.52E-06
				Root vs T0	-7.96	9.44E-07
				Shoot vs T0	-6.32	1.66E-06
				FolRoot vs T0	-4.17	1.92E-04
				FolShoot vs T0	-4.23	1.41E-04
MMB18_RS11400	prfB	peptide chain release factor 2	GO:0006415, GO:0016149, GO:0005737, exception ribosomal slippage	No Seed vs T0	-4.21	4.31E-05
				Root vs T0	-4.94	5.33E-06
				Shoot vs T0	-4.3	1.31E-04
				FolRoot vs T0	-4.5	5.71E-05

				FolShoot vs T0	-5.25	9.13E-06
MMB18_RS11465	NA	lactate utilization protein B	GO:0046872, GO:0051539	No Seed vs T0	-5.98	8.41E-10
				Root vs T0	-5.99	9.62E-10
				Shoot vs T0	-4.84	1.43E-06
				FolRoot vs T0	-4.9	4.06E-07
				FolShoot vs T0	-5.66	2.02E-08
MMB18_RS11470	NA	(Fe-S)-binding protein	GO:0016491, GO:0046872, GO:0051536	No Seed vs T0	-9.2	4.65E-07
				Root vs T0	-6.07	1.31E-05
				Shoot vs T0	-8.34	9.73E-07
				FolRoot vs T0	-7.13	9.59E-06
				FolShoot vs T0	-6.41	1.68E-05
MMB18_RS11490	NA	hypothetical protein	NA	No Seed vs T0	-5.61	3.86E-04
				Root vs T0	-4.12	4.68E-03
				Shoot vs T0	-4.95	1.88E-03
				FolRoot vs T0	-5.21	1.67E-03
				FolShoot vs T0	-3.97	8.67E-03
MMB18_RS11550	NA	prolyl oligopeptidase family serine peptidase	GO:0006508, GO:0004252	No Seed vs T0	-5.73	2.79E-08
				Root vs T0	-5.04	4.73E-07
				Shoot vs T0	-6.27	4.81E-08
				FolRoot vs T0	-4.05	5.93E-05
				FolShoot vs T0	-5.4	4.21E-07
MMB18_RS11580	NA	EI24 domain-containing protein	NA	No Seed vs T0	-3.48	1.20E-04
				Root vs T0	-3.02	8.67E-04
				Shoot vs T0	-5.2	2.65E-07
				FolRoot vs T0	-2.98	1.44E-03
				FolShoot vs T0	-4.87	3.33E-07
MMB18_RS11600	argA	amino-acid N-acetyltransferase	GO:0006526, GO:0004042	No Seed vs T0	-3.77	9.92E-03
				Root vs T0	-7.25	1.70E-05
				Shoot vs T0	-6.51	7.37E-05
				FolRoot vs T0	-5.33	6.36E-04
				FolShoot vs T0	-4.21	4.91E-03
MMB18_RS11605	NA	oxidative damage protection protein	GO:0005506	No Seed vs T0	-4.9	6.90E-04
				Root vs T0	-4.98	7.56E-04
				Shoot vs T0	-4.13	3.82E-03
				FolRoot vs T0	-4.78	2.45E-03
				FolShoot vs T0	-4.17	3.77E-03
MMB18_RS11915	uvrB	excinuclease ABC subunit UvrB	GO:0006289, GO:0003677, GO:0005515, GO:0005524, GO:0016787, GO:0016887, GO:0009380	No Seed vs T0	-10	6.23E-10
				Root vs T0	-8.17	7.04E-10
				Shoot vs T0	-8.31	3.35E-09
				FolRoot vs T0	-6.4	5.56E-07
				FolShoot vs T0	-5.73	4.63E-06
MMB18_RS11925	NA	3-hydroxybutyrate dehydrogenase	NA	No Seed vs T0	-2.36	1.45E-09
				Root vs T0	-2.17	2.28E-08
				Shoot vs T0	-2.13	4.57E-07
				FolRoot vs T0	-2.23	7.45E-08
				FolShoot vs T0	-2.55	1.66E-09
MMB18_RS11950	cueR	Cu(I)-responsive transcriptional regulator	GO:0045893, GO:0046688, GO:0003677, GO:0003700, GO:0005507	No Seed vs T0	-7.18	1.93E-04
				Root vs T0	-6.72	3.32E-04
				Shoot vs T0	-5.25	4.46E-03
				FolRoot vs T0	-8.86	3.61E-05
				FolShoot vs T0	-6.9	3.76E-04
MMB18_RS12010	NA	putative toxin-antitoxin system toxin component, PIN family	GO:0004521, GO:0005515	No Seed vs T0	-7.91	4.66E-08
				Root vs T0	-5.92	5.82E-09
				Shoot vs T0	-5.73	8.92E-09
				FolRoot vs T0	-7.34	7.32E-07
				FolShoot vs T0	-8.25	1.29E-08
MMB18_RS12030	NA	oxepin-CoA hydrolase, alternative type	NA	No Seed vs T0	-6.45	1.10E-04
				Root vs T0	-9.78	1.02E-06
				Shoot vs T0	-6.03	5.25E-04
				FolRoot vs T0	-5.45	1.25E-03
				FolShoot vs T0	-8.38	2.07E-05
MMB18_RS12050	NA	acyl-CoA dehydrogenase family protein	GO:0003995, GO:0050660	No Seed vs T0	-6.1	1.51E-09
				Root vs T0	-5.75	5.93E-09
				Shoot vs T0	-5.26	4.95E-07
				FolRoot vs T0	-6.05	2.04E-08
				FolShoot vs T0	-5.8	3.11E-08
MMB18_RS12055	NA	acyl-CoA dehydrogenase family protein	GO:0016627	No Seed vs T0	-5.66	1.21E-03
				Root vs T0	-7.18	8.13E-05
				Shoot vs T0	-6.7	3.85E-04
				FolRoot vs T0	-5.37	3.06E-03
				FolShoot vs T0	-9.13	1.29E-05

MMB18_RS12095	NA	NADH-quinone oxidoreductase subunit J	GO:0008137	No Seed vs T0	-3.28	6.72E-05
				Root vs T0	-2.64	1.06E-03
				Shoot vs T0	-2.33	7.21E-03
				FolRoot vs T0	-3.3	1.92E-04
				FolShoot vs T0	-4.03	1.56E-05
MMB18_RS12220	NA	acetolactate synthase 3 catalytic subunit	GO:0009082, GO:0003984	No Seed vs T0	-6.34	4.36E-06
				Root vs T0	-6.13	8.61E-06
				Shoot vs T0	-5.49	1.50E-04
				FolRoot vs T0	-6.7	3.17E-06
				FolShoot vs T0	-8.43	5.48E-08
MMB18_RS12285	NA	alanyl-tRNA editing protein	GO:0006419, GO:0002161, GO:0002196, GO:0003676, GO:0004813, GO:0005524, GO:0046872	No Seed vs T0	-5.57	3.82E-03
				Root vs T0	-6.78	8.98E-04
				Shoot vs T0	-7.21	7.14E-04
				FolRoot vs T0	-5.86	4.26E-03
				FolShoot vs T0	-8.71	1.00E-04
MMB18_RS12290	NA	group II truncated hemoglobin	GO:0005344, GO:0019825, GO:0020037, GO:0046872	No Seed vs T0	-6.41	3.04E-07
				Root vs T0	-7.75	6.03E-07
				Shoot vs T0	-5.01	4.24E-06
				FolRoot vs T0	-3.41	4.45E-04
				FolShoot vs T0	-6.5	9.64E-07
MMB18_RS12310	NA	DUF333 domain-containing protein	NA	No Seed vs T0	-4.4	2.21E-04
				Root vs T0	-7.69	5.90E-06
				Shoot vs T0	-3.78	2.76E-03
				FolRoot vs T0	-5.35	1.15E-04
				FolShoot vs T0	-4.03	1.06E-03
MMB18_RS12345	NA	quinone oxidoreductase family protein	GO:0003960, GO:0070402	No Seed vs T0	-5	9.03E-06
				Root vs T0	-6.65	6.33E-08
				Shoot vs T0	-5.96	2.02E-06
				FolRoot vs T0	-3.65	1.44E-03
				FolShoot vs T0	-6.56	3.61E-07
MMB18_RS12395	NA	Maf family protein	GO:0009117, GO:0009146, GO:0000166, GO:0047429	No Seed vs T0	-4.71	1.65E-06
				Root vs T0	-5.69	2.97E-07
				Shoot vs T0	-5.98	4.73E-07
				FolRoot vs T0	-6.54	3.83E-06
				FolShoot vs T0	-4.59	9.74E-06
MMB18_RS12405	NA	YggL family protein	NA	No Seed vs T0	-2.95	2.05E-04
				Root vs T0	-2.89	2.77E-04
				Shoot vs T0	-4.25	3.14E-06
				FolRoot vs T0	-4.15	2.65E-06
				FolShoot vs T0	-4.08	2.76E-06
MMB18_RS12415	NA	MFS transporter	GO:0055085, GO:0022857	No Seed vs T0	-6.7	3.09E-04
				Root vs T0	-5.38	3.41E-03
				Shoot vs T0	-6.1	1.83E-03
				FolRoot vs T0	-5.12	6.96E-03
				FolShoot vs T0	-5.79	2.07E-03
MMB18_RS12445	NA	O-antigen ligase family protein	GO:0009103, GO:0008754, GO:0008755, GO:0005886	No Seed vs T0	-6.4	4.46E-05
				Root vs T0	-4.04	9.36E-03
				Shoot vs T0	-7.68	7.76E-06
				FolRoot vs T0	-5.43	6.99E-04
				FolShoot vs T0	-9.45	8.99E-07
MMB18_RS12450	NA	glycosyltransferase family 9 protein	GO:0016757	No Seed vs T0	-4.05	3.37E-03
				Root vs T0	-7.79	1.42E-06
				Shoot vs T0	-5.04	7.95E-04
				FolRoot vs T0	-5.61	1.38E-04
				FolShoot vs T0	-5.06	4.09E-04
MMB18_RS12520	NA	glycoside hydrolase family 15 protein	NA	No Seed vs T0	-4.91	8.55E-13
				Root vs T0	-5.06	3.67E-13
				Shoot vs T0	-6.31	2.80E-14
				FolRoot vs T0	-3.02	8.76E-06
				FolShoot vs T0	-6.11	5.25E-14
MMB18_RS12550	NA	DUF1841 family protein	NA	No Seed vs T0	-8.97	8.09E-09
				Root vs T0	-5.94	3.40E-07
				Shoot vs T0	-7.17	3.30E-07
				FolRoot vs T0	-7.43	3.55E-06
				FolShoot vs T0	-7.44	6.71E-07
MMB18_RS12555	NA	c-type cytochrome	GO:0009055, GO:0020037, GO:0046872	No Seed vs T0	-3.99	2.94E-05
				Root vs T0	-5.11	1.95E-06
				Shoot vs T0	-4.51	2.44E-05
				FolRoot vs T0	-5.7	1.17E-05
				FolShoot vs T0	-7.08	1.88E-06
MMB18_RS12595	NA	DUF3311 domain-containing protein	NA	No Seed vs T0	-6.75	7.16E-04

				Root vs T0	-6.64	8.92E-04
				Shoot vs T0	-4.72	7.56E-03
				FolRoot vs T0	-7.14	4.66E-04
				FolShoot vs T0	-5.59	2.87E-03
MMB18_RS12635	NA	VOC family protein	NA	No Seed vs T0	-8.08	1.01E-06
				Root vs T0	-9.72	2.78E-08
				Shoot vs T0	-7.19	1.81E-06
				FolRoot vs T0	-4.84	2.36E-04
				FolShoot vs T0	-7.45	1.88E-06
MMB18_RS12655	NA	PhzF family phenazine biosynthesis protein	GO:0009058, GO:0016853, GO:0005737	No Seed vs T0	-3.42	4.45E-04
				Root vs T0	-2.66	6.72E-03
				Shoot vs T0	-3.78	3.06E-04
				FolRoot vs T0	-2.66	8.68E-03
				FolShoot vs T0	-3.4	6.53E-04
MMB18_RS12700	NA	DUF4399 domain-containing protein	NA	No Seed vs T0	-5.88	3.08E-12
				Root vs T0	-5.75	8.33E-12
				Shoot vs T0	-5.34	2.15E-09
				FolRoot vs T0	-6.01	3.88E-11
				FolShoot vs T0	-10.1	1.06E-11
MMB18_RS12740	eco	serine protease inhibitor ecotin	GO:0004867	No Seed vs T0	-5.14	4.72E-04
				Root vs T0	-3.98	3.99E-03
				Shoot vs T0	-5.59	4.39E-04
				FolRoot vs T0	-6.7	3.50E-04
				FolShoot vs T0	-6.6	1.81E-04
MMB18_RS12760	NA	MFS transporter	GO:0055085, GO:0022857, GO:0016020	No Seed vs T0	-2.61	5.18E-05
				Root vs T0	-2.94	9.26E-06
				Shoot vs T0	-4.28	1.21E-07
				FolRoot vs T0	-3.24	8.78E-06
				FolShoot vs T0	-2.87	3.60E-05
MMB18_RS12775	NA	hypothetical protein	NA	No Seed vs T0	-9.15	1.44E-08
				Root vs T0	-8.08	6.46E-07
				Shoot vs T0	-8.12	3.50E-07
				FolRoot vs T0	-7.61	4.71E-06
				FolShoot vs T0	-6.33	1.93E-06
MMB18_RS12780	NA	hypothetical protein	NA	No Seed vs T0	-8.43	6.50E-07
				Root vs T0	-5.93	3.35E-06
				Shoot vs T0	-5.79	1.20E-05
				FolRoot vs T0	-7.86	5.99E-06
				FolShoot vs T0	-7.87	1.47E-06
MMB18_RS12805	NA	ATP-binding protein	NA	No Seed vs T0	-3.19	1.58E-09
				Root vs T0	-3.33	3.92E-10
				Shoot vs T0	-3.24	1.96E-08
				FolRoot vs T0	-2.51	3.80E-06
				FolShoot vs T0	-3.48	5.43E-10
MMB18_RS12870	NA	GNAT family N-acetyltransferase	GO:0008080	No Seed vs T0	-3.97	3.21E-12
				Root vs T0	-3.71	5.03E-11
				Shoot vs T0	-4.01	1.52E-10
				FolRoot vs T0	-3.53	1.74E-09
				FolShoot vs T0	-4.69	5.85E-13
MMB18_RS12920	ppc	phosphoenolpyruvate carboxylase	GO:0006099, GO:0015977, GO:0008964	No Seed vs T0	-3.92	1.11E-22
				Root vs T0	-4.09	2.67E-24
				Shoot vs T0	-3.34	3.24E-15
				FolRoot vs T0	-3.19	2.85E-15
				FolShoot vs T0	-4.18	2.54E-23
MMB18_RS12970	NA	OmpA family protein	GO:0015288, GO:0042834, GO:0009279, GO:0016020	No Seed vs T0	-3.01	9.32E-04
				Root vs T0	-4.84	3.95E-07
				Shoot vs T0	-4.84	2.94E-06
				FolRoot vs T0	-3.33	4.43E-04
				FolShoot vs T0	-5	7.47E-07
MMB18_RS12985	NA	translocation/assembly module TamB domain-containing protein	GO:0009306, GO:0005886	No Seed vs T0	-2.92	3.94E-06
				Root vs T0	-4.24	1.88E-11
				Shoot vs T0	-4.72	3.09E-12
				FolRoot vs T0	-3.75	5.66E-09
				FolShoot vs T0	-4.37	1.14E-11
MMB18_RS13030	NA	cobyric acid synthase	GO:0009236, GO:0003824	No Seed vs T0	-2.19	1.40E-04
				Root vs T0	-2.44	2.35E-05
				Shoot vs T0	-2.17	4.74E-04
				FolRoot vs T0	-2.13	3.65E-04
				FolShoot vs T0	-3.01	6.62E-07
MMB18_RS13045	cobD	threonine-phosphate decarboxylase CobD	GO:0009236, GO:0003824	No Seed vs T0	-2.1	1.70E-05
				Root vs T0	-2.43	9.10E-07

				Shoot vs T0	-2.05	1.14E-04
				FolRoot vs T0	-2.17	2.36E-05
				FolShoot vs T0	-2.8	9.54E-08
MMB18_RS13055	cobC	alpha-ribazole phosphatase	GO:0009236, GO:0043755	No Seed vs T0	-5.84	8.42E-05
				Root vs T0	-5.26	2.88E-04
				Shoot vs T0	-4.99	1.11E-03
				FolRoot vs T0	-6.15	1.69E-04
				FolShoot vs T0	-5.48	3.45E-04
MMB18_RS13140	lptF	LPS export ABC transporter permease LptF	GO:0055085, GO:0015221, GO:0043190	No Seed vs T0	-3.06	1.14E-04
				Root vs T0	-2.82	3.34E-04
				Shoot vs T0	-2.89	7.97E-04
				FolRoot vs T0	-2.37	3.73E-03
				FolShoot vs T0	-2.61	1.39E-03
MMB18_RS13160	NA	sulfate adenylyltransferase subunit 1	NA	No Seed vs T0	-2.79	1.84E-05
				Root vs T0	-3.04	2.93E-06
				Shoot vs T0	-3.88	1.55E-08
				FolRoot vs T0	-4.17	3.32E-10
				FolShoot vs T0	-3.7	2.22E-08
MMB18_RS13165	cysD	sulfate adenylyltransferase subunit CysD	GO:0000103, GO:0004781	No Seed vs T0	-9.99	2.91E-07
				Root vs T0	-7.76	1.94E-06
				Shoot vs T0	-7.51	7.26E-06
				FolRoot vs T0	-9.41	2.35E-06
				FolShoot vs T0	-7	1.72E-05
MMB18_RS13170	NA	phosphoadenylyl-sulfate reductase	GO:0019379, GO:0003824, GO:0004604	No Seed vs T0	-7.8	3.62E-07
				Root vs T0	-7.63	5.95E-07
				Shoot vs T0	-8.53	1.45E-07
				FolRoot vs T0	-5.77	5.24E-06
				FolShoot vs T0	-4	3.44E-04
MMB18_RS13180	NA	nitrite/sulfite reductase	GO:0016491, GO:0020037, GO:0046872, GO:0050311, GO:0051536, GO:0051539	No Seed vs T0	-6.09	4.49E-04
				Root vs T0	-11.6	4.33E-08
				Shoot vs T0	-8.14	1.66E-05
				FolRoot vs T0	-6.08	6.87E-04
				FolShoot vs T0	-5.74	1.14E-03
MMB18_RS13225	NA	LysR family transcriptional regulator	GO:0006355, GO:0003677, GO:0003700	No Seed vs T0	-4.6	1.45E-05
				Root vs T0	-3.62	5.36E-04
				Shoot vs T0	-3.18	4.21E-03
				FolRoot vs T0	-3.3	2.25E-03
				FolShoot vs T0	-4.8	1.62E-05
MMB18_RS13300	NA	branched-chain amino acid ABC transporter permease	GO:0015803, GO:0042626, GO:0140359	No Seed vs T0	-4.42	1.23E-07
				Root vs T0	-5.07	2.57E-09
				Shoot vs T0	-5.42	6.96E-09
				FolRoot vs T0	-5.19	5.48E-09
				FolShoot vs T0	-5.03	1.22E-08
MMB18_RS13335	nadB	L-aspartate oxidase	GO:0009435, GO:0008734	No Seed vs T0	-5.42	5.45E-05
				Root vs T0	-6.52	4.11E-06
				Shoot vs T0	-4.39	1.98E-03
				FolRoot vs T0	-6.16	2.50E-05
				FolShoot vs T0	-6.64	9.77E-06
MMB18_RS13405	clpA	ATP-dependent Clp protease ATP-binding subunit ClpA	GO:0006508, GO:0043335, GO:0004176, GO:0016887, GO:0009368	No Seed vs T0	-4.21	4.10E-10
				Root vs T0	-5.36	5.64E-15
				Shoot vs T0	-4.92	1.60E-11
				FolRoot vs T0	-3.98	7.75E-09
				FolShoot vs T0	-5.4	3.17E-14
MMB18_RS13420	NA	multicopper oxidase family protein	NA	No Seed vs T0	-5.67	3.34E-13
				Root vs T0	-5.99	1.39E-13
				Shoot vs T0	-5.09	5.17E-10
				FolRoot vs T0	-4.48	3.92E-09
				FolShoot vs T0	-5.21	5.96E-11
MMB18_RS13440	NA	pseudouridine synthase	GO:0001522, GO:0009451, GO:0003723, GO:0009982, GO:0016866	No Seed vs T0	-3.78	1.10E-03
				Root vs T0	-5.52	3.05E-06
				Shoot vs T0	-5.67	9.24E-06
				FolRoot vs T0	-3.94	9.90E-04
				FolShoot vs T0	-4.33	2.60E-04
MMB18_RS13490	mdtD	multidrug transporter subunit MdtD	GO:0055085, GO:0022857, GO:0016020	No Seed vs T0	-4.5	1.69E-03
				Root vs T0	-6.75	9.07E-06
				Shoot vs T0	-6.1	1.02E-04
				FolRoot vs T0	-5.55	2.47E-04
				FolShoot vs T0	-5.58	2.00E-04
MMB18_RS13500	NA	class I SAM-dependent methyltransferase	GO:0032259, GO:0008168, GO:1904047	No Seed vs T0	-8.17	1.39E-06
				Root vs T0	-6.48	3.57E-06
				Shoot vs T0	-5.37	4.06E-05

				FolRoot vs T0	-3.41	4.21E-03
				FolShoot vs T0	-7.62	3.16E-06
MMB18_RS13530	xseA	exodeoxyribonuclease VII large subunit	GO:0006308, GO:0008855, GO:0009318	No Seed vs T0	-4.91	1.72E-08
				Root vs T0	-4.56	1.27E-07
				Shoot vs T0	-4.71	6.12E-07
				FolRoot vs T0	-3.77	1.76E-05
				FolShoot vs T0	-7.32	6.33E-10
MMB18_RS13550	adk	adenylate kinase	GO:0006139, GO:0004017, GO:0005524	No Seed vs T0	-9.15	1.47E-07
				Root vs T0	-6.26	1.14E-05
				Shoot vs T0	-5.11	2.16E-04
				FolRoot vs T0	-5.32	1.41E-04
				FolShoot vs T0	-5.87	4.51E-05
MMB18_RS13600	ybaK	Cys-tRNA(Pro) deacylase	GO:0043039, GO:0002161	No Seed vs T0	-7.56	8.07E-09
				Root vs T0	-5.55	3.41E-10
				Shoot vs T0	-4.81	2.48E-08
				FolRoot vs T0	-5.73	1.09E-08
				FolShoot vs T0	-3.84	8.84E-07
MMB18_RS13635	NA	EamA family transporter	GO:0016020	No Seed vs T0	-5.9	1.26E-05
				Root vs T0	-5.82	1.69E-05
				Shoot vs T0	-5.84	4.75E-05
				FolRoot vs T0	-5.46	9.52E-05
				FolShoot vs T0	-8.4	1.38E-06
MMB18_RS13670	msrA	peptide-methionine (S)-S-oxide reductase MsrA	GO:0006979, GO:0030091, GO:0008113, GO:0033744	No Seed vs T0	-8.28	4.05E-06
				Root vs T0	-4.92	2.43E-04
				Shoot vs T0	-6.41	2.72E-05
				FolRoot vs T0	-5.41	2.08E-04
				FolShoot vs T0	-5.18	2.64E-04
MMB18_RS13740	xylB	xylulokinase	GO:0005997, GO:0004856	No Seed vs T0	-2.65	4.74E-03
				Root vs T0	-4.66	9.35E-07
				Shoot vs T0	-4.86	2.70E-06
				FolRoot vs T0	-2.51	9.73E-03
				FolShoot vs T0	-4.13	1.95E-05
MMB18_RS13845	NA	efflux transporter outer membrane subunit	GO:0006810, GO:0015288, GO:0009279	No Seed vs T0	-3.44	1.28E-14
				Root vs T0	-3.68	3.71E-16
				Shoot vs T0	-3.31	7.58E-12
				FolRoot vs T0	-2.63	8.44E-09
				FolShoot vs T0	-2.92	1.66E-10
MMB18_RS13850	bpeB	efflux RND transporter permease BpeB	NA	No Seed vs T0	-5.29	2.45E-11
				Root vs T0	-5.6	2.82E-12
				Shoot vs T0	-3.93	1.99E-06
				FolRoot vs T0	-4.99	7.65E-10
				FolShoot vs T0	-4.34	5.36E-08
MMB18_RS13895	NA	ATP-binding protein	NA	No Seed vs T0	-5.6	3.58E-04
				Root vs T0	-4.36	3.63E-03
				Shoot vs T0	-5	2.05E-03
				FolRoot vs T0	-4.15	7.97E-03
				FolShoot vs T0	-5.2	1.33E-03
MMB18_RS13995	NA	M48 family metalloprotease	GO:0006508, GO:0004222	No Seed vs T0	-3.2	1.64E-05
				Root vs T0	-3.83	4.40E-07
				Shoot vs T0	-3.58	1.07E-05
				FolRoot vs T0	-3.13	5.26E-05
				FolShoot vs T0	-3.11	4.90E-05
MMB18_RS14000	moaC	cyclic pyranopterin monophosphate synthase MoaC	GO:0006777, GO:0061799	No Seed vs T0	-9.79	2.32E-07
				Root vs T0	-6.36	4.00E-05
				Shoot vs T0	-7.45	1.39E-05
				FolRoot vs T0	-7.31	5.05E-05
				FolShoot vs T0	-6.16	1.21E-04
MMB18_RS14075	NA	hypothetical protein	NA	No Seed vs T0	-5.91	1.03E-05
				Root vs T0	-6.96	3.51E-07
				Shoot vs T0	-5.29	1.80E-04
				FolRoot vs T0	-5.25	1.35E-04
				FolShoot vs T0	-4.88	3.07E-04
MMB18_RS14090	NA	hypothetical protein	NA	No Seed vs T0	-3.93	4.51E-03
				Root vs T0	-7.71	2.19E-06
				Shoot vs T0	-6.98	9.42E-06
				FolRoot vs T0	-6.25	4.28E-05
				FolShoot vs T0	-7.64	4.00E-06
MMB18_RS14135	NA	hypothetical protein	NA	No Seed vs T0	-8.88	7.50E-04
				Root vs T0	-7.81	3.24E-03
				Shoot vs T0	-7.77	3.48E-03
				FolRoot vs T0	-8.3	2.20E-03

				FolShoot vs T0	-6.8	8.66E-03
MMB18_RS14145	NA	DUF6088 family protein	NA	No Seed vs T0	-10	4.74E-07
				Root vs T0	-8.96	7.43E-06
				Shoot vs T0	-7.04	7.01E-05
				FolRoot vs T0	-5.23	1.60E-03
				FolShoot vs T0	-6.78	1.11E-04
MMB18_RS14205	NA	LysR family transcriptional regulator	GO:0006355, GO:0003677, GO:0003700	No Seed vs T0	-2.54	5.83E-04
				Root vs T0	-3.53	3.00E-06
				Shoot vs T0	-2.69	7.69E-04
				FolRoot vs T0	-3.13	5.71E-05
				FolShoot vs T0	-2.78	2.80E-04
MMB18_RS14265	NA	DUF1272 domain-containing protein	NA	No Seed vs T0	-8.32	4.17E-08
				Root vs T0	-6.39	4.40E-06
				Shoot vs T0	-6	1.68E-06
				FolRoot vs T0	-5.96	1.99E-05
				FolShoot vs T0	-4.87	1.09E-05
MMB18_RS14310	NA	gamma-glutamyltransferase family protein	GO:0016787	No Seed vs T0	-4.32	1.68E-04
				Root vs T0	-5.28	1.12E-05
				Shoot vs T0	-3.66	2.82E-03
				FolRoot vs T0	-6.05	1.13E-05
				FolShoot vs T0	-3.13	6.99E-03
MMB18_RS14335	glpK	glycerol kinase GlpK	GO:0006072, GO:0004370	No Seed vs T0	-5.05	6.74E-11
				Root vs T0	-5.78	4.80E-12
				Shoot vs T0	-3.79	1.74E-06
				FolRoot vs T0	-3.09	3.53E-05
				FolShoot vs T0	-3.8	5.40E-07
MMB18_RS14385	NA	gamma-glutamylcyclotransferase	NA	No Seed vs T0	-5.67	7.02E-07
				Root vs T0	-6.37	1.07E-07
				Shoot vs T0	-4.06	6.05E-04
				FolRoot vs T0	-5.26	8.85E-06
				FolShoot vs T0	-6.58	3.26E-07
MMB18_RS14430	NA	M48 family metallopeptidase	GO:0006508, GO:0008233, GO:0008237, GO:0008270, GO:0016787, GO:0046872	No Seed vs T0	-2.47	5.79E-07
				Root vs T0	-2.69	5.50E-08
				Shoot vs T0	-3.57	3.20E-11
				FolRoot vs T0	-3.49	1.17E-11
				FolShoot vs T0	-3.49	1.06E-11
MMB18_RS14440	gloA	lactoylglutathione lyase	GO:0051596, GO:0004462, GO:0046872	No Seed vs T0	-5.3	3.09E-08
				Root vs T0	-6.16	5.34E-09
				Shoot vs T0	-4.83	1.79E-06
				FolRoot vs T0	-9.11	5.22E-09
				FolShoot vs T0	-4.93	5.90E-07
MMB18_RS14450	rsmA	16S rRNA (adenine(1518)-N(6)/adenine(1519)-N(6))-dimethyltransferase RsmA	GO:0006364, GO:0000179	No Seed vs T0	-5.77	5.32E-05
				Root vs T0	-9.08	5.18E-07
				Shoot vs T0	-4.25	3.20E-03
				FolRoot vs T0	-4.71	9.73E-04
				FolShoot vs T0	-4.14	2.74E-03
MMB18_RS14480	NA	ABCB family ABC transporter ATP-binding protein/permease	GO:0005524, GO:0016887, GO:0042626, GO:0140359	No Seed vs T0	-4.91	2.40E-09
				Root vs T0	-6.09	1.34E-12
				Shoot vs T0	-4.81	5.14E-08
				FolRoot vs T0	-5.62	1.19E-10
				FolShoot vs T0	-4.63	4.03E-08
MMB18_RS14485	NA	acyl-CoA thioesterase	GO:0016787, GO:0016790	No Seed vs T0	-7.59	1.04E-09
				Root vs T0	-6.48	1.43E-11
				Shoot vs T0	-7.63	2.85E-10
				FolRoot vs T0	-4.94	1.60E-10
				FolShoot vs T0	-6.26	7.68E-11
MMB18_RS14505	NA	acetyl-CoA C-acyltransferase	GO:0016746	No Seed vs T0	-4.5	5.01E-04
				Root vs T0	-6.03	5.95E-06
				Shoot vs T0	-5.52	9.58E-05
				FolRoot vs T0	-5.11	1.57E-04
				FolShoot vs T0	-9.19	1.50E-07
MMB18_RS14510	NA	3-hydroxyacyl-CoA dehydrogenase/enoyl-CoA hydratase family protein	NA	No Seed vs T0	-5.71	6.29E-17
				Root vs T0	-5.44	1.37E-15
				Shoot vs T0	-6.26	2.04E-16
				FolRoot vs T0	-4.08	2.25E-09
				FolShoot vs T0	-4.88	1.58E-12
MMB18_RS14580	NA	HIT family protein	GO:1904047	No Seed vs T0	-5.93	4.04E-06
				Root vs T0	-3.21	5.17E-03
				Shoot vs T0	-4.92	1.32E-04
				FolRoot vs T0	-5.96	2.32E-05
				FolShoot vs T0	-3.61	2.39E-03

MMB18_RS14590	ilvA	threonine ammonia-lyase, biosynthetic	GO:0009097, GO:0004794	No Seed vs T0	-7.66	1.71E-07
				Root vs T0	-8.01	7.36E-08
				Shoot vs T0	-7.46	1.73E-06
				FolRoot vs T0	-5.41	2.31E-04
				FolShoot vs T0	-6.53	8.68E-06
MMB18_RS14605	NA	RidA family protein	NA	No Seed vs T0	-7.47	7.70E-06
				Root vs T0	-6.51	2.61E-05
				Shoot vs T0	-4.67	2.46E-04
				FolRoot vs T0	-7.85	3.72E-06
				FolShoot vs T0	-4.94	1.53E-04
MMB18_RS14635	NA	MDR family MFS transporter	GO:0055085, GO:0022857, GO:0016020	No Seed vs T0	-4.83	6.59E-05
				Root vs T0	-4.54	1.68E-04
				Shoot vs T0	-4.47	6.23E-04
				FolRoot vs T0	-3.21	9.32E-03
				FolShoot vs T0	-3.54	3.68E-03
MMB18_RS14670	NA	FadR/GntR family transcriptional regulator	GO:0003677, GO:0003700	No Seed vs T0	-6.2	2.88E-04
				Root vs T0	-6.61	1.85E-04
				Shoot vs T0	-6.27	5.05E-04
				FolRoot vs T0	-7.01	3.31E-04
				FolShoot vs T0	-6.64	2.79E-04
MMB18_RS14700	NA	glycosyltransferase family 2 protein	GO:0016757	No Seed vs T0	-3.56	7.42E-06
				Root vs T0	-3.17	5.81E-05
				Shoot vs T0	-3.76	1.48E-05
				FolRoot vs T0	-2.79	6.36E-04
				FolShoot vs T0	-2.85	4.11E-04
MMB18_RS14705	NA	endoribonuclease L-PSP	NA	No Seed vs T0	-4.74	4.27E-08
				Root vs T0	-3.61	1.40E-05
				Shoot vs T0	-5.24	7.05E-08
				FolRoot vs T0	-4.66	3.76E-07
				FolShoot vs T0	-5.36	3.12E-08
MMB18_RS14715	NA	phosphopantetheine-binding protein	NA	No Seed vs T0	-3.25	1.45E-09
				Root vs T0	-2.5	7.93E-07
				Shoot vs T0	-2.74	1.04E-06
				FolRoot vs T0	-2.36	1.11E-05
				FolShoot vs T0	-3.59	3.44E-09
MMB18_RS14750	NA	beta-ketoacyl-[acyl-carrier-protein] synthase family protein	NA	No Seed vs T0	-6.16	2.26E-06
				Root vs T0	-3.67	2.69E-03
				Shoot vs T0	-4.37	9.69E-04
				FolRoot vs T0	-3.71	3.44E-03
				FolShoot vs T0	-3.34	7.46E-03
MMB18_RS14755	NA	beta-ketoacyl synthase chain length factor	NA	No Seed vs T0	-5.96	3.13E-05
				Root vs T0	-6.12	2.61E-05
				Shoot vs T0	-6.1	6.31E-05
				FolRoot vs T0	-6.29	6.42E-05
				FolShoot vs T0	-5.36	2.32E-04
MMB18_RS14765	NA	hypothetical protein	NA	No Seed vs T0	-8.02	4.15E-08
				Root vs T0	-5.48	1.70E-06
				Shoot vs T0	-4.09	1.20E-05
				FolRoot vs T0	-6.48	1.74E-05
				FolShoot vs T0	-7.4	5.01E-07
MMB18_RS14775	phbB	acetoacetyl-CoA reductase	GO:0042619, GO:0018454, GO:0005737	No Seed vs T0	-4.91	3.25E-04
				Root vs T0	-3.48	5.21E-03
				Shoot vs T0	-4.61	1.02E-03
				FolRoot vs T0	-3.76	5.21E-03
				FolShoot vs T0	-3.51	7.27E-03
MMB18_RS14795	NA	DNA-3-methyladenine glycosylase	GO:0006284, GO:0003905	No Seed vs T0	-8.77	8.82E-07
				Root vs T0	-5.76	8.08E-05
				Shoot vs T0	-5.45	2.46E-04
				FolRoot vs T0	-4.35	1.93E-03
				FolShoot vs T0	-5.17	4.11E-04
MMB18_RS14830	NA	NUDIX hydrolase	NA	No Seed vs T0	-2.77	1.89E-07
				Root vs T0	-2.55	1.59E-06
				Shoot vs T0	-3.31	2.37E-08
				FolRoot vs T0	-2.88	2.84E-07
				FolShoot vs T0	-3	8.68E-08
MMB18_RS14850	kdsD	arabinose 5-phosphate isomerase KdsD	NA	No Seed vs T0	-4.73	5.87E-08
				Root vs T0	-3.81	3.23E-06
				Shoot vs T0	-3.3	1.43E-04
				FolRoot vs T0	-2.76	7.58E-04
				FolShoot vs T0	-5.64	1.17E-07
MMB18_RS14965	rsmD	16S rRNA (guanine(966)-N(2))-	GO:0031167, GO:0052913	No Seed vs T0	-3.75	1.54E-04

		methyltransferase RsmD		Root vs T0	-5.41	1.31E-07
				Shoot vs T0	-5.5	8.67E-07
				FolRoot vs T0	-3.75	2.55E-04
				FolShoot vs T0	-5.62	1.91E-07
MMB18_RS14975	maiA	maleylacetoacetate isomerase	GO:0006559, GO:0006572, GO:0016034, GO:0005737	No Seed vs T0	-8.56	7.17E-10
				Root vs T0	-8.45	1.02E-09
				Shoot vs T0	-5.54	7.96E-13
				FolRoot vs T0	-5	1.49E-11
				FolShoot vs T0	-8.9	1.41E-10
MMB18_RS15070	NA	ABC transporter ATP-binding protein	NA	No Seed vs T0	-5.71	3.01E-04
				Root vs T0	-8.05	9.97E-06
				Shoot vs T0	-4.75	1.89E-03
				FolRoot vs T0	-3.93	7.76E-03
				FolShoot vs T0	-5.57	5.87E-04
MMB18_RS15075	NA	ABC transporter permease	GO:0042626, GO:0140359, GO:0016020, GO:0043190	No Seed vs T0	-5.73	1.61E-03
				Root vs T0	-7.88	2.03E-04
				Shoot vs T0	-6.23	1.29E-03
				FolRoot vs T0	-4.79	9.62E-03
				FolShoot vs T0	-6.41	1.05E-03
MMB18_RS15180	NA	ComF family protein	GO:0030420	No Seed vs T0	-5.17	4.92E-07
				Root vs T0	-4.2	2.17E-05
				Shoot vs T0	-5.98	2.99E-07
				FolRoot vs T0	-3.5	5.35E-04
				FolShoot vs T0	-5.14	2.16E-06
MMB18_RS15185	trmL	tRNA (uridine(34)/cytosine(34)/5-carboxymethylaminomethyluridine(34)-2'-O)-methyltransferase TrmL	GO:0006400, GO:0008175	No Seed vs T0	-2.42	2.05E-12
				Root vs T0	-2.68	2.73E-14
				Shoot vs T0	-2.74	8.19E-13
				FolRoot vs T0	-2.91	2.11E-14
				FolShoot vs T0	-3.04	2.40E-15
MMB18_RS15225	NA	HesA/MoeB/ThiF family protein	GO:0016779	No Seed vs T0	-9.78	2.02E-09
				Root vs T0	-7.87	2.30E-07
				Shoot vs T0	-6.28	5.46E-07
				FolRoot vs T0	-5.39	4.96E-06
				FolShoot vs T0	-6.55	4.37E-07
MMB18_RS15230	ptsP	phosphoenolpyruvate--protein phosphotransferase	GO:0009401, GO:0008965	No Seed vs T0	-7.45	3.71E-11
				Root vs T0	-5.81	1.33E-07
				Shoot vs T0	-7.28	1.82E-09
				FolRoot vs T0	-7.64	7.10E-11
				FolShoot vs T0	-7.17	4.94E-10
MMB18_RS15250	gshA	glutamate--cysteine ligase	GO:0006750, GO:0004357	No Seed vs T0	-5.54	2.79E-08
				Root vs T0	-4.14	2.63E-05
				Shoot vs T0	-3.2	1.92E-03
				FolRoot vs T0	-4.8	2.52E-06
				FolShoot vs T0	-4.57	5.45E-06
MMB18_RS15290	NA	sensor histidine kinase	GO:0007165, GO:0000155, GO:0004673, GO:0005524	No Seed vs T0	-6.83	6.56E-07
				Root vs T0	-5.94	1.57E-06
				Shoot vs T0	-4.05	6.41E-04
				FolRoot vs T0	-3.31	3.12E-03
				FolShoot vs T0	-7.72	1.18E-06
MMB18_RS15295	NA	dicarboxylate/amino acid:cation symporter	GO:0006835, GO:0015293	No Seed vs T0	-6.52	1.45E-12
				Root vs T0	-6.39	3.70E-12
				Shoot vs T0	-5.43	5.90E-09
				FolRoot vs T0	-6.33	7.10E-11
				FolShoot vs T0	-5.88	2.24E-10
MMB18_RS15330	NA	LysR family transcriptional regulator	GO:0006355, GO:0003677, GO:0003700	No Seed vs T0	-4.27	3.80E-05
				Root vs T0	-4.49	1.69E-05
				Shoot vs T0	-5.31	4.24E-06
				FolRoot vs T0	-4.2	9.76E-05
				FolShoot vs T0	-4.73	1.30E-05
MMB18_RS15420	NA	ABC transporter ATP-binding protein	GO:0005524, GO:0016887, GO:0042626, GO:0140359	No Seed vs T0	-3.69	6.01E-05
				Root vs T0	-4.81	6.20E-07
				Shoot vs T0	-2.89	3.09E-03
				FolRoot vs T0	-3.99	4.11E-05
				FolShoot vs T0	-4.53	5.06E-06
MMB18_RS15455	NA	hypothetical protein	NA	No Seed vs T0	-8.5	9.07E-06
				Root vs T0	-8.4	1.16E-05
				Shoot vs T0	-6.67	2.40E-04
				FolRoot vs T0	-6.96	4.25E-04
				FolShoot vs T0	-6.99	1.99E-04
MMB18_RS15470	NA	patatin-like phospholipase family protein	GO:0052689	No Seed vs T0	-7.09	8.74E-09
				Root vs T0	-6.95	1.60E-08

				Shoot vs T0	-4.85	9.57E-05
				FolRoot vs T0	-7.26	3.97E-08
				FolShoot vs T0	-7.4	2.76E-08
MMB18_RS15475	NA	PhaM family polyhydroxyalkanoate granule multifunctional regulatory protein	NA	No Seed vs T0	-4.39	2.16E-04
				Root vs T0	-4.2	3.89E-04
				Shoot vs T0	-5.15	1.02E-04
				FolRoot vs T0	-4.81	2.40E-04
				FolShoot vs T0	-5.13	1.15E-04
MMB18_RS15480	NA	enoyl-CoA hydratase/isomerase family protein	NA	No Seed vs T0	-4.54	5.92E-05
				Root vs T0	-3.29	3.72E-03
				Shoot vs T0	-4.71	9.57E-05
				FolRoot vs T0	-3.92	7.46E-04
				FolShoot vs T0	-5.33	5.38E-06
MMB18_RS15495	NA	DUF3108 domain-containing protein	NA	No Seed vs T0	-3.6	3.53E-05
				Root vs T0	-3.69	2.61E-05
				Shoot vs T0	-4.03	3.11E-05
				FolRoot vs T0	-3.16	4.61E-04
				FolShoot vs T0	-2.83	1.38E-03
MMB18_RS15555	NA	MBL fold metallo-hydrolase	NA	No Seed vs T0	-4.41	7.01E-07
				Root vs T0	-5.06	2.36E-08
				Shoot vs T0	-4.16	1.23E-05
				FolRoot vs T0	-3.17	4.97E-04
				FolShoot vs T0	-3.56	7.75E-05
MMB18_RS15560	aceK	bifunctional isocitrate dehydrogenase kinase/phosphatase	GO:0006006, GO:0008772, GO:0016791	No Seed vs T0	-4.29	3.91E-03
				Root vs T0	-4.08	6.18E-03
				Shoot vs T0	-5.12	1.10E-03
				FolRoot vs T0	-4.19	6.38E-03
				FolShoot vs T0	-5.89	1.14E-04
MMB18_RS15590	bioF	8-amino-7-oxononanoate synthase	GO:0009102, GO:0008710	No Seed vs T0	-4.64	2.04E-06
				Root vs T0	-4.41	5.65E-06
				Shoot vs T0	-2.61	9.25E-03
				FolRoot vs T0	-2.84	3.71E-03
				FolShoot vs T0	-3.49	3.47E-04
MMB18_RS15600	bioB	biotin synthase BioB	GO:0009102, GO:0004076, GO:0051536, GO:1904047	No Seed vs T0	-8.54	2.51E-09
				Root vs T0	-5.71	3.40E-08
				Shoot vs T0	-4.5	2.57E-05
				FolRoot vs T0	-6.48	2.42E-08
				FolShoot vs T0	-5.92	7.16E-08
MMB18_RS15615	NA	hypothetical protein	NA	No Seed vs T0	-6.86	5.20E-06
				Root vs T0	-8.09	3.38E-06
				Shoot vs T0	-4.61	6.11E-04
				FolRoot vs T0	-6.81	3.42E-05
				FolShoot vs T0	-4.92	2.07E-04
MMB18_RS15625	NA	Na+/H+ antiporter	GO:0006814, GO:0006885, GO:1902600, GO:0015385, GO:0016020	No Seed vs T0	-2.89	4.46E-21
				Root vs T0	-3.32	1.53E-24
				Shoot vs T0	-2.55	5.99E-15
				FolRoot vs T0	-2.62	4.24E-16
				FolShoot vs T0	-2.56	1.36E-15
MMB18_RS15655	NA	sulfate ABC transporter substrate-binding protein	GO:0005524, GO:0042626, GO:0140359	No Seed vs T0	-6.43	3.04E-05
				Root vs T0	-7.36	7.79E-06
				Shoot vs T0	-4.95	1.92E-03
				FolRoot vs T0	-4.24	5.62E-03
				FolShoot vs T0	-7.34	1.61E-05
MMB18_RS15670	NA	type II toxin-antitoxin system Phd/YefM family antitoxin	GO:0110001	No Seed vs T0	-4.12	2.14E-05
				Root vs T0	-4.93	2.24E-06
				Shoot vs T0	-7.05	5.46E-07
				FolRoot vs T0	-4.08	8.08E-05
				FolShoot vs T0	-4.77	1.14E-05
MMB18_RS15690	NA	LysR substrate-binding domain-containing protein	NA	No Seed vs T0	-6.25	2.66E-05
				Root vs T0	-5.15	3.39E-04
				Shoot vs T0	-3.96	8.44E-03
				FolRoot vs T0	-4.72	1.44E-03
				FolShoot vs T0	-5.44	2.85E-04
MMB18_RS15745	NA	NAD(P)H-dependent flavin oxidoreductase	GO:0004497, GO:0010181, GO:0016703	No Seed vs T0	-5.43	1.06E-05
				Root vs T0	-5.99	2.59E-06
				Shoot vs T0	-4.57	4.62E-04
				FolRoot vs T0	-3.57	3.92E-03
				FolShoot vs T0	-6.74	1.94E-06
MMB18_RS15750	NA	LysR family transcriptional regulator	GO:0006355, GO:0001216, GO:0032993	No Seed vs T0	-4.95	6.22E-12
				Root vs T0	-5.08	6.07E-12
				Shoot vs T0	-4.95	2.78E-10

				FolRoot vs T0	-4.77	4.18E-10
				FolShoot vs T0	-5.17	7.39E-11
MMB18_RS15755	NA	porin	GO:0015288, GO:0009279, GO:0016020	No Seed vs T0	-7.26	2.48E-05
				Root vs T0	-7.43	1.86E-05
				Shoot vs T0	-6.39	3.99E-04
				FolRoot vs T0	-5.81	8.25E-04
				FolShoot vs T0	-5.78	7.48E-04
MMB18_RS15780	NA	AGE family epimerase/isomerase	NA	No Seed vs T0	-3.49	2.12E-12
				Root vs T0	-3.4	7.62E-12
				Shoot vs T0	-2.97	1.77E-08
				FolRoot vs T0	-2.08	2.79E-05
				FolShoot vs T0	-3.45	3.89E-11
MMB18_RS16080	NA	5-formyltetrahydrofolate cyclo-ligase	GO:0006730, GO:0030272	No Seed vs T0	-4.38	5.28E-05
				Root vs T0	-4.58	2.85E-05
				Shoot vs T0	-5.65	3.92E-06
				FolRoot vs T0	-4.05	3.19E-04
				FolShoot vs T0	-6.36	7.82E-07
MMB18_RS16115	NA	ABC transporter ATP-binding protein	GO:0016887, GO:0042626, GO:0140359	No Seed vs T0	-5.54	6.74E-15
				Root vs T0	-4.86	3.10E-12
				Shoot vs T0	-4.7	3.31E-10
				FolRoot vs T0	-3.55	4.25E-07
				FolShoot vs T0	-3.38	1.21E-06
MMB18_RS16125	NA	ABC transporter permease subunit	GO:0055085, GO:0016020	No Seed vs T0	-3.39	2.50E-06
				Root vs T0	-3.96	6.41E-08
				Shoot vs T0	-4.17	2.09E-07
				FolRoot vs T0	-3.62	1.72E-06
				FolShoot vs T0	-4.12	8.12E-08
MMB18_RS16135	NA	MFS transporter	GO:0055085, GO:0022857	No Seed vs T0	-5.59	2.38E-10
				Root vs T0	-4.78	1.28E-08
				Shoot vs T0	-5.26	1.92E-08
				FolRoot vs T0	-4.64	1.17E-07
				FolShoot vs T0	-4.89	3.70E-08
MMB18_RS16145	NA	NAD(P)/FAD-dependent oxidoreductase	GO:0000166, GO:0016491	No Seed vs T0	-4.33	1.31E-08
				Root vs T0	-5.17	3.58E-11
				Shoot vs T0	-3.83	2.38E-06
				FolRoot vs T0	-4.29	4.93E-08
				FolShoot vs T0	-4.38	2.63E-08
MMB18_RS16190	NA	XdhC family protein	GO:0070403	No Seed vs T0	-3.04	5.40E-08
				Root vs T0	-3.45	9.61E-10
				Shoot vs T0	-3.71	1.82E-09
				FolRoot vs T0	-2.68	3.52E-06
				FolShoot vs T0	-3.49	2.63E-09
MMB18_RS16285	mgrA	L-glyceraldehyde 3-phosphate reductase	GO:0047834	No Seed vs T0	-6.14	4.28E-15
				Root vs T0	-7.1	1.37E-15
				Shoot vs T0	-4.87	6.75E-10
				FolRoot vs T0	-5.21	1.64E-11
				FolShoot vs T0	-6.06	2.64E-13
MMB18_RS16335	NA	class I SAM-dependent rRNA methyltransferase	GO:0006364, GO:0008168, GO:1904047	No Seed vs T0	-5.9	9.09E-07
				Root vs T0	-5.03	1.99E-05
				Shoot vs T0	-5.85	6.40E-06
				FolRoot vs T0	-3.34	5.86E-03
				FolShoot vs T0	-4.17	4.65E-04
MMB18_RS16390	metX	homoserine O-succinyltransferase MetX	GO:0009086, GO:0008899	No Seed vs T0	-5.04	3.20E-06
				Root vs T0	-4.69	1.40E-05
				Shoot vs T0	-6.39	1.72E-07
				FolRoot vs T0	-4.9	1.30E-05
				FolShoot vs T0	-5.2	3.80E-06
MMB18_RS16400	NA	AmpG family muropeptide MFS transporter	NA	No Seed vs T0	-4.02	1.62E-09
				Root vs T0	-4.34	1.33E-10
				Shoot vs T0	-4.12	1.32E-08
				FolRoot vs T0	-2.9	1.99E-05
				FolShoot vs T0	-3.55	1.99E-07
MMB18_RS16405	NA	M48 family metallopeptidase	GO:0006508, GO:0004222, GO:0046872	No Seed vs T0	-4.15	1.78E-05
				Root vs T0	-6.04	9.74E-09
				Shoot vs T0	-5.45	6.57E-07
				FolRoot vs T0	-4.52	9.47E-06
				FolShoot vs T0	-5.72	1.32E-07
MMB18_RS16565	NA	patatin-like phospholipase family protein	GO:0052689	No Seed vs T0	-4.04	2.35E-08
				Root vs T0	-4.84	1.31E-10
				Shoot vs T0	-4.74	5.90E-09
				FolRoot vs T0	-3.22	1.37E-05

				FolShoot vs T0	-3.82	3.28E-07
MMB18_RS16580	NA	DUF3592 domain-containing protein	NA	No Seed vs T0	-2.32	2.47E-03
				Root vs T0	-3.72	1.24E-06
				Shoot vs T0	-4.21	5.45E-07
				FolRoot vs T0	-3.15	6.45E-05
				FolShoot vs T0	-4.34	7.69E-08
MMB18_RS16585	NA	CGNR zinc finger domain-containing protein	GO:0003676, GO:0008270	No Seed vs T0	-5.29	2.88E-07
				Root vs T0	-5.85	1.41E-08
				Shoot vs T0	-7.24	1.35E-10
				FolRoot vs T0	-10.4	9.67E-13
				FolShoot vs T0	-12.4	2.99E-14
MMB18_RS16600	NA	amino acid ABC transporter permease	GO:0006865, GO:0042626, GO:0140359	No Seed vs T0	-4.18	1.48E-07
				Root vs T0	-4.32	8.75E-08
				Shoot vs T0	-3.86	6.78E-06
				FolRoot vs T0	-4.84	6.91E-08
				FolShoot vs T0	-4.57	1.34E-07
MMB18_RS16645	NA	phosphonate utilization associated transcriptional regulator	NA	No Seed vs T0	-5.8	2.82E-05
				Root vs T0	-6.43	4.90E-06
				Shoot vs T0	-6.72	8.58E-06
				FolRoot vs T0	-5.07	3.62E-04
				FolShoot vs T0	-5.05	3.20E-04
MMB18_RS16685	NA	VOC family protein	NA	No Seed vs T0	-9.69	3.27E-08
				Root vs T0	-8.62	1.01E-06
				Shoot vs T0	-6.26	1.20E-05
				FolRoot vs T0	-5.9	3.17E-05
				FolShoot vs T0	-6.34	1.23E-05
MMB18_RS16695	sap1	surface attachment protein Sap1	NA	No Seed vs T0	-8.37	5.11E-07
				Root vs T0	-9.23	2.62E-08
				Shoot vs T0	-8.3	4.17E-07
				FolRoot vs T0	-6.89	7.98E-06
				FolShoot vs T0	-8.71	1.75E-07
MMB18_RS16720	NA	hypothetical protein	NA	No Seed vs T0	-11.5	2.77E-07
				Root vs T0	-8.06	3.81E-05
				Shoot vs T0	-8.9	1.94E-05
				FolRoot vs T0	-5.05	9.41E-03
				FolShoot vs T0	-9.37	1.18E-05
MMB18_RS17095	NA	alkene reductase	GO:0010181, GO:0016491	No Seed vs T0	-4.08	3.81E-07
				Root vs T0	-5.84	2.82E-12
				Shoot vs T0	-5.22	3.29E-09
				FolRoot vs T0	-5.14	1.05E-09
				FolShoot vs T0	-5.17	8.65E-10
MMB18_RS17100	NA	ArsR/SmtB family transcription factor	GO:0006355, GO:0003677, GO:0003700	No Seed vs T0	-3.91	6.89E-03
				Root vs T0	-7.63	5.48E-05
				Shoot vs T0	-4.48	4.21E-03
				FolRoot vs T0	-5.88	6.87E-04
				FolShoot vs T0	-6.13	2.86E-04
MMB18_RS17225	NA	TetR/AcrR family transcriptional regulator	GO:0006355, GO:0003677, GO:0003700	No Seed vs T0	-5.01	1.76E-07
				Root vs T0	-4.63	1.02E-06
				Shoot vs T0	-4.27	2.44E-05
				FolRoot vs T0	-3.01	1.52E-03
				FolShoot vs T0	-4.67	2.31E-06
MMB18_RS17340	ribA	GTP cyclohydrolase II	GO:0003935	No Seed vs T0	-3.69	2.95E-06
				Root vs T0	-3.7	2.96E-06
				Shoot vs T0	-3.5	3.36E-05
				FolRoot vs T0	-3.06	1.66E-04
				FolShoot vs T0	-3.19	7.51E-05
MMB18_RS17400	NA	hypothetical protein	NA	No Seed vs T0	-4.7	8.36E-04
				Root vs T0	-4.97	5.28E-04
				Shoot vs T0	-5.04	9.50E-04
				FolRoot vs T0	-7.28	1.19E-04
				FolShoot vs T0	-4.05	4.79E-03
MMB18_RS17425	NA	hypothetical protein	NA	No Seed vs T0	-3.22	1.63E-04
				Root vs T0	-4.31	1.71E-05
				Shoot vs T0	-2.88	1.51E-03
				FolRoot vs T0	-4.32	1.12E-04
				FolShoot vs T0	-3.04	7.53E-04
MMB18_RS17875	NA	helix-turn-helix domain-containing protein	GO:0006355, GO:0003677	No Seed vs T0	-3.83	7.22E-09
				Root vs T0	-3.19	5.59E-07
				Shoot vs T0	-2.7	6.86E-05
				FolRoot vs T0	-2.81	2.06E-05
				FolShoot vs T0	-2.83	1.63E-05

MMB18_RS17950	NA	LysR family transcriptional regulator	GO:0006355, GO:0001216, GO:0032993	No Seed vs T0	-3.22	2.81E-05
				Root vs T0	-7.79	1.35E-07
				Shoot vs T0	-4.87	2.39E-07
				FolRoot vs T0	-2.08	8.05E-03
				FolShoot vs T0	-4.29	1.18E-06
MMB18_RS18015	NA	alpha/beta hydrolase	GO:0016787	No Seed vs T0	-4.1	1.69E-05
				Root vs T0	-3.44	2.61E-04
				Shoot vs T0	-3.17	1.68E-03
				FolRoot vs T0	-2.95	2.49E-03
				FolShoot vs T0	-2.9	2.50E-03
MMB18_RS18215	NA	isochorismatase family protein	NA	No Seed vs T0	-4.65	1.40E-05
				Root vs T0	-4.5	2.38E-05
				Shoot vs T0	-3.06	2.69E-03
				FolRoot vs T0	-3.44	8.02E-04
				FolShoot vs T0	-3.19	1.35E-03
MMB18_RS18255	mgtA	magnesium-translocating P-type ATPase	GO:0015693, GO:0015444, GO:0016020	No Seed vs T0	-4.7	3.05E-07
				Root vs T0	-4.7	3.56E-07
				Shoot vs T0	-3.83	8.02E-05
				FolRoot vs T0	-3.18	6.69E-04
				FolShoot vs T0	-3.5	1.52E-04
MMB18_RS18320	NA	porin	GO:0015288, GO:0009279	No Seed vs T0	-3.24	9.12E-05
				Root vs T0	-3.45	3.29E-05
				Shoot vs T0	-2.72	2.07E-03
				FolRoot vs T0	-2.94	6.12E-04
				FolShoot vs T0	-2.82	8.56E-04
MMB18_RS18595	NA	virulence factor family protein	NA	No Seed vs T0	-6.99	3.94E-09
				Root vs T0	-5.65	2.53E-07
				Shoot vs T0	-4.83	2.70E-05
				FolRoot vs T0	-4.43	5.65E-05
				FolShoot vs T0	-4.74	1.63E-05
MMB18_RS18745	NA	LysR family transcriptional regulator	GO:0006355, GO:0003677, GO:0003700	No Seed vs T0	-4.46	1.29E-07
				Root vs T0	-2.93	2.21E-04
				Shoot vs T0	-2.38	4.95E-03
				FolRoot vs T0	-2.98	3.38E-04
				FolShoot vs T0	-2.59	1.47E-03
MMB18_RS19300	NA	type II toxin-antitoxin system MqsR family toxin	NA	No Seed vs T0	-7.12	3.59E-04
				Root vs T0	-7.77	1.91E-04
				Shoot vs T0	-6.99	4.39E-04
				FolRoot vs T0	-6.07	2.01E-03
				FolShoot vs T0	-8.21	7.96E-05
MMB18_RS19450	NA	(2Fe-2S)-binding protein	GO:0046872, GO:0051536, GO:0051537	No Seed vs T0	-4.97	3.19E-03
				Root vs T0	-7.54	1.03E-04
				Shoot vs T0	-4.79	5.47E-03
				FolRoot vs T0	-5.3	5.15E-03
				FolShoot vs T0	-4.76	6.06E-03
MMB18_RS19670	NA	penicillin-binding protein 1A	GO:0006508, GO:0009252, GO:0008658, GO:0008955, GO:0009002	No Seed vs T0	-2.6	9.29E-08
				Root vs T0	-3.1	3.45E-10
				Shoot vs T0	-2.03	1.01E-04
				FolRoot vs T0	-2.35	3.46E-06
				FolShoot vs T0	-2.34	3.03E-06
MMB18_RS19820	NA	DUF1493 family protein	NA	No Seed vs T0	-6.28	5.61E-08
				Root vs T0	-7.85	1.66E-07
				Shoot vs T0	-8.73	3.67E-08
				FolRoot vs T0	-4.87	1.17E-05
				FolShoot vs T0	-7.59	1.28E-07
MMB18_RS19915	NA	LysR family transcriptional regulator	GO:0006355, GO:0003677, GO:0003700	No Seed vs T0	-5.88	1.71E-05
				Root vs T0	-4.8	1.74E-04
				Shoot vs T0	-3.85	4.00E-03
				FolRoot vs T0	-3.56	5.64E-03
				FolShoot vs T0	-4.3	9.60E-04
MMB18_RS19995	kch	voltage-gated potassium channel protein	NA	No Seed vs T0	-3.05	1.07E-04
				Root vs T0	-3.7	3.95E-06
				Shoot vs T0	-3.58	3.57E-05
				FolRoot vs T0	-4.57	2.59E-07
				FolShoot vs T0	-3.72	8.54E-06
MMB18_RS20540	NA	LysR family transcriptional regulator	GO:0006355, GO:0003677, GO:0003700	No Seed vs T0	-4.75	4.21E-08
				Root vs T0	-4.45	2.40E-07
				Shoot vs T0	-3.65	5.88E-05
				FolRoot vs T0	-3.91	9.60E-06
				FolShoot vs T0	-4.18	2.31E-06
MMB18_RS21350	NA	MarR family winged helix-turn-helix	GO:0006355, GO:0003700	No Seed vs T0	-4.62	7.70E-05

		transcriptional regulator		Root vs T0	-5.89	6.93E-05
				Shoot vs T0	-4.71	1.44E-04
				FolRoot vs T0	-3.89	9.78E-04
				FolShoot vs T0	-5.17	1.04E-04
MMB18_RS21595	selB	selenocysteine-specific translation elongation factor	GO:0001514, GO:0003723, GO:0003746, GO:0003924, GO:0005525, GO:0005737	No Seed vs T0	-4.59	9.54E-06
				Root vs T0	-3.63	3.01E-04
				Shoot vs T0	-2.8	8.44E-03
				FolRoot vs T0	-3.5	8.04E-04
				FolShoot vs T0	-3.17	1.98E-03
MMB18_RS21755	NA	phosphoribosylanthranilate isomerase	GO:0000162, GO:0004640	No Seed vs T0	-4.1	2.32E-04
				Root vs T0	-6.24	2.30E-07
				Shoot vs T0	-5.11	3.34E-05
				FolRoot vs T0	-4.43	1.44E-04
				FolShoot vs T0	-4.3	1.84E-04
MMB18_RS21780	leuB	3-isopropylmalate dehydrogenase	GO:0009098, GO:0003862	No Seed vs T0	-3.84	3.10E-03
				Root vs T0	-5.85	5.65E-06
				Shoot vs T0	-4.22	1.63E-03
				FolRoot vs T0	-6.61	7.65E-07
				FolShoot vs T0	-4.43	6.72E-04
MMB18_RS21785	leuD	3-isopropylmalate dehydratase small subunit	GO:0009098, GO:0003861, GO:0009316	No Seed vs T0	-2.32	2.49E-05
				Root vs T0	-2.62	1.87E-06
				Shoot vs T0	-2.61	9.23E-06
				FolRoot vs T0	-2.19	1.19E-04
				FolShoot vs T0	-2.05	2.66E-04
MMB18_RS21970	NA	triacylglycerol lipase	NA	No Seed vs T0	-3.77	8.13E-09
				Root vs T0	-6.04	1.54E-13
				Shoot vs T0	-3.35	1.84E-06
				FolRoot vs T0	-4.83	1.18E-10
				FolShoot vs T0	-5.1	2.83E-11
MMB18_RS22040	NA	mechanosensitive ion channel domain-containing protein	GO:0055085, GO:0016020	No Seed vs T0	-4.65	8.69E-09
				Root vs T0	-3.96	5.87E-07
				Shoot vs T0	-2.75	9.75E-04
				FolRoot vs T0	-2.44	2.52E-03
				FolShoot vs T0	-2.87	3.20E-04
MMB18_RS22170	NA	dienelactone hydrolase family protein	GO:0016787	No Seed vs T0	-6.15	1.93E-06
				Root vs T0	-4.17	3.58E-04
				Shoot vs T0	-4.7	2.42E-04
				FolRoot vs T0	-3.54	3.29E-03
				FolShoot vs T0	-4.66	1.55E-04
MMB18_RS22175	NA	NAD(P)/FAD-dependent oxidoreductase	GO:0006116, GO:0003954	No Seed vs T0	-5.92	5.67E-06
				Root vs T0	-5.26	5.07E-05
				Shoot vs T0	-4.12	2.42E-03
				FolRoot vs T0	-3.54	8.38E-03
				FolShoot vs T0	-4.72	3.33E-04
MMB18_RS22180	polA	DNA polymerase I	GO:0006261, GO:0003676, GO:0003677, GO:0003887, GO:0008408	No Seed vs T0	-3.6	1.35E-10
				Root vs T0	-3.62	1.16E-10
				Shoot vs T0	-3.3	4.02E-08
				FolRoot vs T0	-3.07	9.13E-08
				FolShoot vs T0	-3.65	3.13E-10
MMB18_RS22185	NA	TIGR00730 family Rossman fold protein	GO:0009691, GO:0016787	No Seed vs T0	-4.5	5.33E-04
				Root vs T0	-5.13	9.55E-05
				Shoot vs T0	-6.45	9.03E-06
				FolRoot vs T0	-4.67	5.55E-04
				FolShoot vs T0	-4.13	1.88E-03
MMB18_RS22195	NA	AMP nucleosidase	GO:0009116, GO:0044209, GO:0008714	No Seed vs T0	-7.81	8.58E-09
				Root vs T0	-5.98	8.11E-07
				Shoot vs T0	-5.47	2.13E-05
				FolRoot vs T0	-5.04	3.94E-05
				FolShoot vs T0	-4.21	4.38E-04
MMB18_RS22255	NA	TOBE domain-containing protein	NA	No Seed vs T0	-4.72	7.95E-06
				Root vs T0	-3.87	1.20E-04
				Shoot vs T0	-4.73	3.00E-05
				FolRoot vs T0	-3.86	2.85E-04
				FolShoot vs T0	-3.08	2.39E-03
MMB18_RS22270	NA	LysR family transcriptional regulator	GO:0006355, GO:0003677, GO:0003700	No Seed vs T0	-3.61	9.97E-06
				Root vs T0	-5.48	6.69E-10
				Shoot vs T0	-4.03	6.77E-06
				FolRoot vs T0	-5.08	2.51E-08
				FolShoot vs T0	-4.74	9.05E-08
MMB18_RS22275	NA	DMT family transporter	GO:0055085, GO:0022857, GO:0016020	No Seed vs T0	-4.66	1.47E-04
				Root vs T0	-5.59	1.08E-05

				Shoot vs T0	-3.95	2.48E-03
				FolRoot vs T0	-5.67	2.39E-05
				FolShoot vs T0	-3.73	2.76E-03
MMB18_RS22560	NA	voltage-gated chloride channel family protein	NA	No Seed vs T0	-2.3	4.95E-03
				Root vs T0	-3.53	1.65E-05
				Shoot vs T0	-3.47	8.13E-05
				FolRoot vs T0	-2.45	3.74E-03
				FolShoot vs T0	-2.51	2.53E-03
MMB18_RS22795	NA	AraC family transcriptional regulator	GO:0006355, GO:0003677, GO:0003700	No Seed vs T0	-3.57	2.82E-05
				Root vs T0	-4.21	1.04E-06
				Shoot vs T0	-3.21	4.27E-04
				FolRoot vs T0	-2.4	6.57E-03
				FolShoot vs T0	-3.93	8.25E-06
MMB18_RS23095	argG	argininosuccinate synthase	GO:0006526, GO:0004055, GO:0005524, GO:0042803	No Seed vs T0	-3.65	1.22E-03
				Root vs T0	-4.89	1.56E-05
				Shoot vs T0	-4.09	5.92E-04
				FolRoot vs T0	-4.63	6.88E-05
				FolShoot vs T0	-4.97	1.73E-05
MMB18_RS23210	NA	DUF1176 domain-containing protein	NA	No Seed vs T0	-4.46	2.81E-06
				Root vs T0	-2.71	2.28E-03
				Shoot vs T0	-2.55	7.66E-03
				FolRoot vs T0	-2.57	5.56E-03
				FolShoot vs T0	-3.36	3.46E-04
MMB18_RS23275	NA	phosphotransferase enzyme family protein	GO:0016310, GO:0005524, GO:0016301	No Seed vs T0	-5.54	2.83E-05
				Root vs T0	-6.49	1.10E-06
				Shoot vs T0	-5.46	8.79E-05
				FolRoot vs T0	-5.81	1.99E-05
				FolShoot vs T0	-5.91	1.18E-05
MMB18_RS23375	NA	DUF802 domain-containing protein	NA	No Seed vs T0	-3.96	2.82E-67
				Root vs T0	-3.51	1.95E-53
				Shoot vs T0	-3.37	3.29E-44
				FolRoot vs T0	-3.3	2.03E-46
				FolShoot vs T0	-3.11	4.07E-41
MMB18_RS23795	NA	DUF2760 domain-containing protein	NA	No Seed vs T0	-5.97	8.87E-06
				Root vs T0	-4.4	2.68E-04
				Shoot vs T0	-3.77	3.21E-03
				FolRoot vs T0	-4.48	4.56E-04
				FolShoot vs T0	-4.94	1.51E-04
MMB18_RS24230	NA	MarR family winged helix-turn-helix transcriptional regulator	GO:0006355, GO:0003700	No Seed vs T0	-3.22	4.81E-03
				Root vs T0	-3.95	5.55E-04
				Shoot vs T0	-3.54	3.63E-03
				FolRoot vs T0	-4.26	3.65E-04
				FolShoot vs T0	-4.56	1.31E-04
MMB18_RS24310	NA	GNAT family N-acetyltransferase	GO:0008080	No Seed vs T0	-9.1	2.35E-08
				Root vs T0	-4.54	3.79E-05
				Shoot vs T0	-3.59	1.89E-03
				FolRoot vs T0	-4.18	2.44E-04
				FolShoot vs T0	-3.21	3.17E-03
MMB18_RS24320	NA	LysR substrate-binding domain-containing protein	NA	No Seed vs T0	-6.41	1.37E-06
				Root vs T0	-4.65	2.35E-04
				Shoot vs T0	-4.3	1.42E-03
				FolRoot vs T0	-4.63	4.12E-04
				FolShoot vs T0	-5.04	1.19E-04
MMB18_RS24325	NA	hypothetical protein	NA	No Seed vs T0	-5.54	1.45E-04
				Root vs T0	-5.96	4.98E-05
				Shoot vs T0	-5.51	4.16E-04
				FolRoot vs T0	-4.88	1.15E-03
				FolShoot vs T0	-4.51	2.28E-03
MMB18_RS24495	NA	DUF2946 domain-containing protein	NA	No Seed vs T0	-3.39	1.82E-05
				Root vs T0	-4.81	3.32E-07
				Shoot vs T0	-4	9.19E-06
				FolRoot vs T0	-4.33	6.77E-06
				FolShoot vs T0	-2.8	5.21E-04
MMB18_RS24580	NA	Lrp/AsnC family transcriptional regulator	GO:0043565	No Seed vs T0	-3.21	6.40E-05
				Root vs T0	-2.35	2.56E-03
				Shoot vs T0	-2.4	4.76E-03
				FolRoot vs T0	-3.18	2.21E-04
				FolShoot vs T0	-3.15	2.02E-04
MMB18_RS24620	NA	TetR/AcrR family transcriptional regulator	GO:0006355, GO:0003677, GO:0003700	No Seed vs T0	-5.32	3.09E-05
				Root vs T0	-7.12	4.68E-08
				Shoot vs T0	-7.94	2.48E-08

				FolRoot vs T0	-5.84	9.49E-06
				FolShoot vs T0	-6.43	1.03E-06
MMB18_RS24625	NA	efflux RND transporter periplasmic adaptor subunit	GO:0055085, GO:0022857, GO:0042802, GO:0016020	No Seed vs T0	-4.02	2.14E-05
				Root vs T0	-4.95	4.74E-07
				Shoot vs T0	-3.51	5.28E-04
				FolRoot vs T0	-3.35	5.73E-04
				FolShoot vs T0	-3.03	1.55E-03
MMB18_RS25470	atsR	hybrid sensor histidine kinase/response regulator AtsR	NA	No Seed vs T0	-5	3.42E-09
				Root vs T0	-3.24	4.98E-05
				Shoot vs T0	-3.15	2.46E-04
				FolRoot vs T0	-2.83	6.01E-04
				FolShoot vs T0	-2.36	3.63E-03
MMB18_RS25535	surE	5'/3'-nucleotidase SurE	GO:0008252	No Seed vs T0	-3.05	7.37E-04
				Root vs T0	-5.3	3.33E-09
				Shoot vs T0	-5.24	3.43E-08
				FolRoot vs T0	-3.56	1.19E-04
				FolShoot vs T0	-4.39	1.28E-06
MMB18_RS25695	NA	response regulator transcription factor	GO:0000160	No Seed vs T0	-7.32	2.72E-10
				Root vs T0	-6.53	2.93E-09
				Shoot vs T0	-5.15	4.78E-06
				FolRoot vs T0	-5.12	2.38E-06
				FolShoot vs T0	-7.2	3.95E-09
MMB18_RS25880	mqo	malate dehydrogenase (quinone)	GO:0006099, GO:0008924	No Seed vs T0	-3.9	2.42E-07
				Root vs T0	-3.31	1.15E-05
				Shoot vs T0	-3.53	1.06E-05
				FolRoot vs T0	-4.67	3.87E-09
				FolShoot vs T0	-3.36	1.23E-05
MMB18_RS26230	NA	LysR substrate-binding domain-containing protein	NA	No Seed vs T0	-4.39	5.94E-05
				Root vs T0	-4.78	1.67E-05
				Shoot vs T0	-3.55	2.21E-03
				FolRoot vs T0	-3.93	5.05E-04
				FolShoot vs T0	-3.04	5.81E-03
MMB18_RS26375	NA	universal stress protein	GO:0042802	No Seed vs T0	-5.1	1.57E-05
				Root vs T0	-5.02	2.37E-05
				Shoot vs T0	-2.97	4.35E-03
				FolRoot vs T0	-2.67	7.97E-03
				FolShoot vs T0	-3.84	3.82E-04
MMB18_RS26480	NA	3-hydroxybutyrate dehydrogenase	NA	No Seed vs T0	-2.25	4.48E-05
				Root vs T0	-2.01	2.70E-04
				Shoot vs T0	-2.38	8.69E-05
				FolRoot vs T0	-2.27	1.02E-04
				FolShoot vs T0	-2.64	7.77E-06
MMB18_RS26590	NA	RNA polymerase factor sigma-70	NA	No Seed vs T0	-4.64	5.40E-04
				Root vs T0	-5.4	1.01E-04
				Shoot vs T0	-4.14	4.03E-03
				FolRoot vs T0	-4.94	5.50E-04
				FolShoot vs T0	-6.7	2.50E-05
MMB18_RS26610	NA	response regulator	GO:0000160, GO:0000156	No Seed vs T0	-4.7	6.44E-05
				Root vs T0	-8.22	5.24E-07
				Shoot vs T0	-4.16	6.47E-04
				FolRoot vs T0	-3.69	1.67E-03
				FolShoot vs T0	-6.16	2.77E-05
MMB18_RS26715	NA	DUF6306 domain-containing protein	NA	No Seed vs T0	-6.15	9.01E-06
				Root vs T0	-5.96	1.30E-05
				Shoot vs T0	-3.7	1.76E-03
				FolRoot vs T0	-3.76	1.19E-03
				FolShoot vs T0	-4.23	3.52E-04
MMB18_RS26945	NA	SDR family oxidoreductase	GO:0016491	No Seed vs T0	-5.01	4.14E-04
				Root vs T0	-5.63	1.04E-04
				Shoot vs T0	-4.14	5.86E-03
				FolRoot vs T0	-7.46	2.62E-05
				FolShoot vs T0	-4.74	1.17E-03
MMB18_RS26965	NA	PRC-barrel domain-containing protein	GO:0019684, GO:0009055	No Seed vs T0	-5.31	1.24E-07
				Root vs T0	-9.03	3.87E-09
				Shoot vs T0	-3.2	9.48E-04
				FolRoot vs T0	-4.22	1.80E-05
				FolShoot vs T0	-5.68	5.26E-07
MMB18_RS27150	merR	Hg(II)-responsive transcriptional regulator	GO:0006355, GO:0046689, GO:0003677, GO:0045340	No Seed vs T0	-4.97	2.53E-04
				Root vs T0	-7.48	3.03E-06
				Shoot vs T0	-4.81	1.02E-03
				FolRoot vs T0	-4.17	2.86E-03

				FolShoot vs T0	-4.45	1.34E-03
MMB18_RS27190	dmeF	CDF family Co(II)/Ni(II) efflux transporter DmeF	GO:0006812, GO:0055085, GO:0008324, GO:0016020	No Seed vs T0	-5.33	2.80E-07
				Root vs T0	-5.07	1.03E-06
				Shoot vs T0	-4.35	6.55E-05
				FolRoot vs T0	-4.72	9.59E-06
				FolShoot vs T0	-3.29	1.77E-03
MMB18_RS27195	NA	metal/formaldehyde-sensitive transcriptional repressor	NA	No Seed vs T0	-8.83	1.82E-07
				Root vs T0	-7.11	4.54E-06
				Shoot vs T0	-7.83	2.87E-06
				FolRoot vs T0	-5.1	1.05E-03
				FolShoot vs T0	-5.1	9.00E-04
MMB18_RS27355	NA	hypothetical protein	NA	No Seed vs T0	-3.67	2.20E-03
				Root vs T0	-4.31	3.03E-04
				Shoot vs T0	-4.83	9.88E-05
				FolRoot vs T0	-4.38	3.47E-04
				FolShoot vs T0	-4.27	3.82E-04
MMB18_RS27480	NA	H-NS family nucleoid-associated regulatory protein	GO:0006355, GO:0003677, GO:0030527	No Seed vs T0	-4.3	2.60E-03
				Root vs T0	-6.31	1.27E-04
				Shoot vs T0	-4.25	5.50E-03
				FolRoot vs T0	-4.84	1.80E-03
				FolShoot vs T0	-3.92	7.70E-03
MMB18_RS27600	NA	DUF1254 domain-containing protein	NA	No Seed vs T0	-4.25	1.28E-09
				Root vs T0	-3.81	3.91E-08
				Shoot vs T0	-2.94	6.25E-05
				FolRoot vs T0	-4.2	8.68E-09
				FolShoot vs T0	-4.82	1.74E-10
MMB18_RS27690	ltaE	low-specificity L-threonine aldolase	GO:0006520, GO:0016829	No Seed vs T0	-2.99	6.20E-11
				Root vs T0	-2.61	8.88E-09
				Shoot vs T0	-2.66	5.84E-08
				FolRoot vs T0	-2.05	1.23E-05
				FolShoot vs T0	-2.33	5.62E-07
MMB18_RS28350	ceoR	putative multidrug efflux transcriptional regulator CeoR	NA	No Seed vs T0	-5.39	4.58E-07
				Root vs T0	-5.48	3.59E-07
				Shoot vs T0	-4.71	3.23E-05
				FolRoot vs T0	-4.92	8.05E-06
				FolShoot vs T0	-6.65	4.36E-08
MMB18_RS28500	NA	site-2 protease family protein	NA	No Seed vs T0	-5.82	4.17E-08
				Root vs T0	-6.29	5.24E-07
				Shoot vs T0	-3.53	1.66E-06
				FolRoot vs T0	-4.11	7.65E-07
				FolShoot vs T0	-6.7	4.57E-07
MMB18_RS28540	NA	DoxX family protein	GO:0005886	No Seed vs T0	-6.42	6.30E-05
				Root vs T0	-7.11	3.57E-05
				Shoot vs T0	-4.57	7.73E-04
				FolRoot vs T0	-3.52	6.03E-03
				FolShoot vs T0	-5	3.74E-04
MMB18_RS29480	NA	energy transducer TonB	GO:0015031, GO:0015891, GO:0055085, GO:0031992, GO:0030288	No Seed vs T0	-5.35	6.96E-09
				Root vs T0	-5.93	4.68E-08
				Shoot vs T0	-2.59	3.29E-04
				FolRoot vs T0	-3.55	4.02E-06
				FolShoot vs T0	-3.85	8.38E-07
MMB18_RS29755	NA	APC family permease	GO:0055085, GO:0022857, GO:0016020	No Seed vs T0	-6.75	9.03E-09
				Root vs T0	-5.88	3.62E-07
				Shoot vs T0	-5.02	3.37E-05
				FolRoot vs T0	-4.88	3.43E-05
				FolShoot vs T0	-5.78	1.01E-06
MMB18_RS30240	NA	DUF2000 domain-containing protein	NA	No Seed vs T0	-8.56	2.21E-06
				Root vs T0	-6.07	1.20E-04
				Shoot vs T0	-6.55	8.21E-05
				FolRoot vs T0	-7.02	1.68E-04
				FolShoot vs T0	-4.8	1.18E-03
MMB18_RS30250	NA	AraC family transcriptional regulator	NA	No Seed vs T0	-5.37	4.44E-10
				Root vs T0	-4.55	6.34E-08
				Shoot vs T0	-2.97	7.63E-04
				FolRoot vs T0	-2.37	6.22E-03
				FolShoot vs T0	-4.58	1.42E-07
MMB18_RS30420	NA	DUF2917 domain-containing protein	NA	No Seed vs T0	-6.7	9.54E-06
				Root vs T0	-4.38	7.88E-04
				Shoot vs T0	-5.58	1.23E-04
				FolRoot vs T0	-4.19	2.02E-03
				FolShoot vs T0	-6.34	3.34E-05

MMB18_RS30430	NA	hypothetical protein	NA	No Seed vs T0	-6.11	4.92E-04
				Root vs T0	-8.35	1.99E-05
				Shoot vs T0	-4.6	6.30E-03
				FolRoot vs T0	-5.24	2.95E-03
				FolShoot vs T0	-5.9	9.44E-04
MMB18_RS30640	NA	LysR family transcriptional regulator	GO:0006355, GO:0003677, GO:0003700	No Seed vs T0	-7.9	5.61E-06
				Root vs T0	-4.03	3.67E-03
				Shoot vs T0	-4.29	4.00E-03
				FolRoot vs T0	-4.48	2.00E-03
				FolShoot vs T0	-5.36	2.87E-04
MMB18_RS31250	NA	AraC family transcriptional regulator	NA	No Seed vs T0	-6.53	1.29E-10
				Root vs T0	-3.76	1.79E-05
				Shoot vs T0	-4.55	2.70E-06
				FolRoot vs T0	-4.87	2.70E-07
				FolShoot vs T0	-3.95	1.32E-05
MMB18_RS31290	NA	TOBE domain-containing protein	GO:0015689	No Seed vs T0	-7.3	2.38E-04
				Root vs T0	-5.77	1.12E-03
				Shoot vs T0	-4.84	7.26E-03
				FolRoot vs T0	-4.9	6.43E-03
				FolShoot vs T0	-4.92	5.59E-03
MMB18_RS31365	NA	three-Cys-motif partner protein TcmP	NA	No Seed vs T0	-8.26	7.84E-07
				Root vs T0	-5.78	7.16E-05
				Shoot vs T0	-4.17	5.72E-03
				FolRoot vs T0	-6.28	4.73E-05
				FolShoot vs T0	-4.36	2.82E-03
MMB18_RS31370	NA	DUF5131 family protein	NA	No Seed vs T0	-6.38	3.70E-05
				Root vs T0	-6.44	3.25E-05
				Shoot vs T0	-5.18	1.36E-03
				FolRoot vs T0	-7.29	8.16E-06
				FolShoot vs T0	-5.16	9.68E-04
MMB18_RS31910	NA	hypothetical protein	NA	No Seed vs T0	-9.33	2.38E-10
				Root vs T0	-7.11	5.40E-07
				Shoot vs T0	-7.33	1.05E-06
				FolRoot vs T0	-5.89	5.05E-05
				FolShoot vs T0	-7.35	3.60E-07
MMB18_RS31940	NA	ThiF family adenylyltransferase	GO:0008641	No Seed vs T0	-4.71	9.22E-10
				Root vs T0	-4.84	3.41E-10
				Shoot vs T0	-3.83	2.36E-06
				FolRoot vs T0	-4.1	1.83E-07
				FolShoot vs T0	-4.12	1.42E-07
MMB18_RS32085	NA	LysR substrate-binding domain-containing protein	NA	No Seed vs T0	-6.5	5.21E-07
				Root vs T0	-6.71	3.50E-07
				Shoot vs T0	-4.84	2.85E-04
				FolRoot vs T0	-3.73	3.65E-03
				FolShoot vs T0	-4.83	1.50E-04
MMB18_RS32250	NA	lysozyme inhibitor LprI family protein	NA	No Seed vs T0	-3.93	3.08E-11
				Root vs T0	-3.79	1.31E-10
				Shoot vs T0	-2.39	1.01E-04
				FolRoot vs T0	-3.08	2.70E-07
				FolShoot vs T0	-2.53	1.72E-05
MMB18_RS32355	NA	MFS transporter	NA	No Seed vs T0	-7.26	3.72E-13
				Root vs T0	-5.72	9.87E-10
				Shoot vs T0	-5.06	3.44E-07
				FolRoot vs T0	-5.19	5.59E-08
				FolShoot vs T0	-3.8	4.73E-05
MMB18_RS32630	NA	ankyrin repeat domain-containing protein	NA	No Seed vs T0	-3.5	1.33E-12
				Root vs T0	-3.33	1.35E-11
				Shoot vs T0	-2.75	1.23E-07
				FolRoot vs T0	-3.32	1.27E-10
				FolShoot vs T0	-3.11	1.02E-09
MMB18_RS32635	shc	squalene--hopene cyclase	GO:0019746, GO:0016866, GO:0051007	No Seed vs T0	-4.44	2.42E-08
				Root vs T0	-4.02	4.02E-07
				Shoot vs T0	-2.6	1.78E-03
				FolRoot vs T0	-2.78	6.37E-04
				FolShoot vs T0	-2.41	2.67E-03
MMB18_RS32640	NA	TetR/AcrR family transcriptional regulator	GO:0006355, GO:0003677, GO:0003700	No Seed vs T0	-4.66	2.08E-05
				Root vs T0	-4.63	2.44E-05
				Shoot vs T0	-3.53	2.19E-03
				FolRoot vs T0	-4.92	2.23E-05
				FolShoot vs T0	-3.32	2.55E-03
MMB18_RS33005	NA	DUF4337 domain-containing protein	NA	No Seed vs T0	-8.49	3.81E-08

				Root vs T0	-4.33	1.38E-05
				Shoot vs T0	-3.4	8.35E-04
				FolRoot vs T0	-3.68	2.70E-04
				FolShoot vs T0	-3.23	8.76E-04
MMB18_RS33555	NA	GlxA family transcriptional regulator	GO:0006355, GO:0003677, GO:0003700	No Seed vs T0	-8.66	3.57E-08
				Root vs T0	-5.21	1.19E-04
				Shoot vs T0	-4.98	5.25E-04
				FolRoot vs T0	-5.08	2.76E-04
				FolShoot vs T0	-3.89	4.53E-03
MMB18_RS33675	NA	LysR family transcriptional regulator	GO:0006355, GO:0003677, GO:0003700	No Seed vs T0	-2.87	4.86E-07
				Root vs T0	-2.57	6.76E-06
				Shoot vs T0	-2.55	2.54E-05
				FolRoot vs T0	-2.82	1.69E-06
				FolShoot vs T0	-2.5	1.75E-05
MMB18_RS35115	NA	Dyp-type peroxidase	NA	No Seed vs T0	-3.21	2.21E-06
				Root vs T0	-2.72	6.20E-05
				Shoot vs T0	-4.01	4.02E-08
				FolRoot vs T0	-3.29	2.50E-06
				FolShoot vs T0	-3.12	6.06E-06
MMB18_RS35125	NA	HNH endonuclease family protein	NA	No Seed vs T0	-8.7	7.62E-17
				Root vs T0	-9.82	5.64E-15
				Shoot vs T0	-6.77	1.06E-11
				FolRoot vs T0	-7.75	2.35E-14
				FolShoot vs T0	-10.1	6.10E-14
MMB18_RS35370	NA	hypothetical protein	NA	No Seed vs T0	-3.27	2.57E-06
				Root vs T0	-4.35	4.47E-09
				Shoot vs T0	-3.77	1.27E-06
				FolRoot vs T0	-2.63	2.44E-04
				FolShoot vs T0	-2.61	2.35E-04
MMB18_RS36100	NA	TetR/AcrR family transcriptional regulator	GO:0006355, GO:0003677, GO:0003700	No Seed vs T0	-5.62	4.04E-06
				Root vs T0	-4.59	4.84E-05
				Shoot vs T0	-3.84	1.17E-03
				FolRoot vs T0	-5.11	4.97E-05
				FolShoot vs T0	-4.47	1.43E-04
MMB18_RS36920	NA	IS5-like element ISBmu23 family transposase	GO:0006313, GO:0004803	No Seed vs T0	-4.71	4.62E-05
				Root vs T0	-5.39	3.91E-06
				Shoot vs T0	-5.69	6.78E-06
				FolRoot vs T0	-4.25	3.71E-04
				FolShoot vs T0	-3.87	9.79E-04
MMB18_RS37315	NA	ATP-binding protein	NA	No Seed vs T0	-3.2	1.15E-09
				Root vs T0	-3.5	9.66E-11
				Shoot vs T0	-2.95	2.09E-07
				FolRoot vs T0	-2.84	2.17E-07
				FolShoot vs T0	-3.98	3.17E-11
MMB18_RS37795	NA	hypothetical protein	NA	No Seed vs T0	-6.53	5.83E-07
				Root vs T0	-6.24	1.26E-06
				Shoot vs T0	-6.84	1.21E-06
				FolRoot vs T0	-5.19	4.64E-05
				FolShoot vs T0	-9.31	6.50E-08
MMB18_RS37810	NA	ATP-binding protein	NA	No Seed vs T0	-6.05	1.57E-08
				Root vs T0	-3.3	2.24E-03
				Shoot vs T0	-4.63	3.23E-05
				FolRoot vs T0	-4.4	6.13E-05
				FolShoot vs T0	-4.87	6.61E-06
MMB18_RS37820	NA	CbrC family protein	NA	No Seed vs T0	-7.04	6.16E-09
				Root vs T0	-6.36	6.39E-10
				Shoot vs T0	-3.33	6.32E-08
				FolRoot vs T0	-2.76	1.79E-06
				FolShoot vs T0	-5.79	5.70E-10
MMB18_RS37870	NA	tyrosine-type recombinase/integrase	GO:0006310, GO:0009009	No Seed vs T0	-3.69	2.17E-06
				Root vs T0	-4.26	1.60E-07
				Shoot vs T0	-3.8	9.23E-06
				FolRoot vs T0	-2.76	4.94E-04
				FolShoot vs T0	-3.97	2.06E-06
MMB18_RS38755	NA	IS256-like element IS931 family transposase	GO:0006313, GO:0004803	No Seed vs T0	-5.45	1.40E-04
				Root vs T0	-5.54	1.08E-04
				Shoot vs T0	-7.5	1.38E-06
				FolRoot vs T0	-6.26	2.05E-05
				FolShoot vs T0	-5.84	5.49E-05
MMB18_RS38805	NA	IS30 family transposase	NA	No Seed vs T0	-8.52	4.11E-05
				Root vs T0	-5.45	2.61E-03

				Shoot vs T0	-6.68	8.35E-04
				FolRoot vs T0	-6.19	2.44E-03
				FolShoot vs T0	-5.01	6.81E-03
MMB18_RS38815	istB	IS21-like element helper ATPase IstB	GO:0005524	No Seed vs T0	-4.71	4.74E-09
				Root vs T0	-4.34	5.56E-08
				Shoot vs T0	-3.95	3.41E-06
				FolRoot vs T0	-2.44	3.01E-03
				FolShoot vs T0	-3.23	6.17E-05
MMB18_RS38860	NA	hypothetical protein	NA	No Seed vs T0	-6.03	1.37E-05
				Root vs T0	-5.36	1.36E-05
				Shoot vs T0	-6.08	8.58E-06
				FolRoot vs T0	-7.3	3.05E-06
				FolShoot vs T0	-5.61	2.04E-05
MMB18_RS39030	NA	recombinase family protein	GO:0006310, GO:0000150, GO:0003677	No Seed vs T0	-6.84	7.62E-04
				Root vs T0	-8.85	1.15E-04
				Shoot vs T0	-8.83	1.14E-04
				FolRoot vs T0	-6.18	3.13E-03
				FolShoot vs T0	-6.12	3.00E-03
MMB18_RS39045	NA	DNA translocase FtsK	GO:0007059, GO:0051301, GO:0003677, GO:0005524, GO:0016887	No Seed vs T0	-3.05	2.60E-03
				Root vs T0	-4	7.21E-05
				Shoot vs T0	-3.59	6.70E-04
				FolRoot vs T0	-2.84	6.61E-03
				FolShoot vs T0	-3.85	1.56E-04

Supplemental Table 2: The 110 B. contaminans genes involved in four secondary metabolite biosynthesis: ornibactin, pyochelin, occidiofungin, and pyrrolnitrin.

Gene ID	Cluster	Gene Name	Gene Product	Gene Ontology
MMB18_RS08275	Ornibactin	NA	ABC transporter substrate-binding protein	NA
MMB18_RS08280	Ornibactin	NA	amino acid ABC transporter permease	GO:0006865, GO:0042626, GO:0140359
MMB18_RS08285	Ornibactin	NA	amino acid ABC transporter permease	GO:0006865, GO:0042626, GO:0140359
MMB18_RS08290	Ornibactin	NA	M23 family metallopeptidase	GO:0008237, GO:0046872
MMB18_RS08295	Ornibactin	NA	TetR family transcriptional regulator	GO:0003677
MMB18_RS08300	Ornibactin	NA	MexX/AxyX family multidrug efflux RND transporter periplasmic adaptor subunit	NA
MMB18_RS08305	Ornibactin	NA	multidrug efflux RND transporter permease subunit	NA
MMB18_RS08310	Ornibactin	NA	efflux transporter outer membrane subunit	GO:0006810, GO:0015288, GO:0009279
MMB18_RS08320	Ornibactin	NA	fimbria/pilus outer membrane usher protein	GO:0009297, GO:0015473, GO:0016020
MMB18_RS08345	Ornibactin	NA	RNA polymerase factor sigma-70	GO:0006352, GO:0003677, GO:0016987
MMB18_RS08350	Ornibactin	NA	MbtH family protein	NA
MMB18_RS08355	Ornibactin	NA	TauD/TfdA family dioxygenase	GO:0016491
MMB18_RS08360	Ornibactin	NA	ABC transporter ATP-binding protein	GO:0005524, GO:0016887, GO:0042626, GO:0140359
MMB18_RS08365	Ornibactin	fhuB	Fe(3+)-hydroxamate ABC transporter permease FhuB	NA
MMB18_RS08370	Ornibactin	fhuF	siderophore-iron reductase FhuF	GO:0033214, GO:0008199, GO:0016491, GO:0051537
MMB18_RS08375	Ornibactin	NA	ABC transporter substrate-binding protein	GO:0022857, GO:0042626, GO:0140359, GO:0055052
MMB18_RS08380	Ornibactin	NA	cyclic peptide export ABC transporter	GO:0015833, GO:0005524, GO:1904680, GO:0016020
MMB18_RS08385	Ornibactin	NA	non-ribosomal peptide synthetase	NA
MMB18_RS08390	Ornibactin	NA	non-ribosomal peptide synthetase	NA
MMB18_RS08395	Ornibactin	NA	GNAT family N-acetyltransferase	GO:0008080, GO:0016746
MMB18_RS08400	Ornibactin	NA	lysine N(6)-hydroxylase/L-ornithine N(5)-oxygenase family protein	NA
MMB18_RS08405	Ornibactin	NA	TonB-dependent siderophore receptor	GO:0015891, GO:0055085, GO:0015267, GO:0046872, GO:0009279, GO:0016020
MMB18_RS08440	Ornibactin	NA	type IV toxin-antitoxin system AbiEi family antitoxin	NA
MMB18_RS08460	Ornibactin	NA	cobyrinate a,c-diamide synthase	GO:0042242
MMB18_RS08465	Ornibactin	cobO	cob(I)yrinic acid a,c-diamide adenosyltransferase	GO:0009236, GO:0008817
MMB18_RS08475	Ornibactin	cobA	uroporphyrinogen-III C-methyltransferase	GO:0019354, GO:0004851
MMB18_RS08480	Ornibactin	NA	EAL domain-containing protein	NA
MMB18_RS08485	Ornibactin	NA	HoxN/HupN/NixA family nickel/cobalt transporter	NA
MMB18_RS08490	Ornibactin	cobW	cobalamin biosynthesis protein CobW	GO:0009236
MMB18_RS30170	Pyochelin	NA	SymE family type I addiction module toxin	GO:0016070, GO:0003723, GO:0016788, GO:0005737
MMB18_RS30175	Pyochelin	NA	hypothetical protein	NA
MMB18_RS30190	Pyochelin	NA	high-affinity branched-chain amino acid ABC transporter permease LivM	GO:0015658, GO:0140359, GO:0016020, GO:0043190
MMB18_RS30200	Pyochelin	NA	ABC transporter ATP-binding protein	GO:0005524, GO:0016887, GO:0042626, GO:0140359

MMB18_RS30205	Pyochelin	NA	LysR substrate-binding domain-containing protein	NA
MMB18_RS30215	Pyochelin	NA	DMT family transporter	GO:0055085, GO:0022857, GO:0016020
MMB18_RS30235	Pyochelin	NA	hypothetical protein	NA
MMB18_RS30240	Pyochelin	NA	DUF2000 domain-containing protein	NA
MMB18_RS30245	Pyochelin	NA	AraC family transcriptional regulator	GO:0006355, GO:0003700, GO:0043565
MMB18_RS30250	Pyochelin	NA	AraC family transcriptional regulator	NA
MMB18_RS30265	Pyochelin	NA	helix-turn-helix transcriptional regulator	GO:0006355, GO:0003677, GO:0003700
MMB18_RS30270	Pyochelin	NA	aspartate aminotransferase family protein	GO:0008483, GO:0030170
MMB18_RS30275	Pyochelin	NA	LysR family transcriptional regulator	NA
MMB18_RS30280	Pyochelin	NA	isochorismate synthase	NA
MMB18_RS30295	Pyochelin	NA	(2,3-dihydroxybenzoyl)adenylate synthase	GO:0019290, GO:0008668
MMB18_RS30300	Pyochelin	NA	helix-turn-helix transcriptional regulator	GO:0006355, GO:0003700, GO:0043565
MMB18_RS30305	Pyochelin	NA	non-ribosomal peptide synthetase	NA
MMB18_RS30310	Pyochelin	NA	non-ribosomal peptide synthetase	NA
MMB18_RS30320	Pyochelin	NA	ABC transporter ATP-binding protein	GO:0005524, GO:0016887, GO:0042626, GO:0140359, GO:0055052
MMB18_RS30325	Pyochelin	NA	ABC transporter ATP-binding protein	GO:0005524, GO:0016887, GO:0042626, GO:0140359, GO:0055052
MMB18_RS30330	Pyochelin	NA	TonB-dependent siderophore receptor	GO:0015891, GO:0055085, GO:0015267, GO:0046872, GO:0009279, GO:0016020
MMB18_RS30340	Pyochelin	NA	PepSY-associated TM helix domain-containing protein	GO:0016020
MMB18_RS30345	Pyochelin	NA	RhtX/FptX family siderophore transporter	GO:0015891, GO:0015343
MMB18_RS30355	Pyochelin	NA	leucine-rich repeat-containing protein kinase family protein	GO:0006468, GO:0005515, GO:0005524
MMB18_RS30360	Pyochelin	NA	LysE family translocator	GO:0006865, GO:0016020
MMB18_RS30365	Pyochelin	NA	hypothetical protein	NA
MMB18_RS30375	Pyochelin	copC	copper homeostasis periplasmic binding protein CopC	GO:0006825, GO:0046688, GO:0005507, GO:0042597
MMB18_RS30380	Pyochelin	NA	DUF2946 domain-containing protein	NA
MMB18_RS30395	Pyochelin	NA	sigma-54-dependent Fis family transcriptional regulator	GO:0000160, GO:0006355, GO:0005524, GO:0043565
MMB18_RS30400	Pyochelin	NA	DUF2964 domain-containing protein	NA
MMB18_RS30405	Pyochelin	NA	DUF1488 domain-containing protein	NA
MMB18_RS30410	Pyochelin	NA	NCS2 family permease	GO:0015851, GO:0015205, GO:0005886
MMB18_RS32595	Occidiofungin	NA	glycoside hydrolase domain-containing protein	NA
MMB18_RS32600	Occidiofungin	NA	class I SAM-dependent methyltransferase	GO:0032259, GO:0008168, GO:0008757, GO:1904047
MMB18_RS32605	Occidiofungin	NA	hypothetical protein	NA
MMB18_RS32610	Occidiofungin	NA	alpha/beta fold hydrolase	NA
MMB18_RS32620	Occidiofungin	ampC	class C beta-lactamase	GO:0008800, GO:0030288
MMB18_RS32630	Occidiofungin	NA	ankyrin repeat domain-containing protein	NA
MMB18_RS32635	Occidiofungin	shc	squalene--hopene cyclase	GO:0019746, GO:0016866, GO:0051007

MMB18_RS32640	Occidiofungin	NA	TetR/AcrR family transcriptional regulator	GO:0006355, GO:0003677, GO:0003700
MMB18_RS32645	Occidiofungin	NA	cholesterol oxidase substrate-binding domain-containing protein	NA
MMB18_RS32655	Occidiofungin	NA	response regulator transcription factor	GO:0000160, GO:0006355, GO:0003677
MMB18_RS32660	Occidiofungin	NA	helix-turn-helix transcriptional regulator	GO:0006355, GO:0003677, GO:0003700
MMB18_RS32665	Occidiofungin	NA	cyclic peptide export ABC transporter	GO:0015833, GO:0005524, GO:1904680, GO:0016020
MMB18_RS32675	Occidiofungin	NA	glycosyltransferase family 25 protein	NA
MMB18_RS32680	Occidiofungin	NA	non-ribosomal peptide synthetase	NA
MMB18_RS32685	Occidiofungin	NA	non-ribosomal peptide synthetase	NA
MMB18_RS32690	Occidiofungin	NA	non-ribosomal peptide synthetase	NA
MMB18_RS32695	Occidiofungin	NA	MBL fold metallo-hydrolase	NA
MMB18_RS32700	Occidiofungin	NA	hybrid non-ribosomal peptide synthetase/type I polyketide synthase	NA
MMB18_RS32705	Occidiofungin	NA	bifunctional LLM class flavin-dependent oxidoreductase/SDR family oxidoreductase	GO:0016491, GO:0043783
MMB18_RS32710	Occidiofungin	NA	beta-ketoacyl synthase N-terminal-like domain-containing protein	NA
MMB18_RS32715	Occidiofungin	NA	TauD/TfdA family dioxygenase	GO:0016491
MMB18_RS32720	Occidiofungin	NA	diaminobutyrate--2-oxoglutarate transaminase family protein	GO:0019491, GO:0008483
MMB18_RS32735	Occidiofungin	NA	hypothetical protein	NA
MMB18_RS32740	Occidiofungin	NA	formylglycine-generating enzyme family protein	NA
MMB18_RS32750	Occidiofungin	NA	methyl-accepting chemotaxis protein	GO:0007165, GO:0016020
MMB18_RS32760	Occidiofungin	NA	ABC transporter ATP-binding protein	GO:0006810, GO:0005215, GO:0005524, GO:0016887, GO:0042626, GO:0140359
MMB18_RS36945	Pyrrolnitrin	NA	ABC transporter permease	GO:0042626, GO:0140359, GO:0016020, GO:0043190
MMB18_RS36950	Pyrrolnitrin	NA	ABC transporter permease	GO:0042626, GO:0140359, GO:0016020, GO:0043190
MMB18_RS36955	Pyrrolnitrin	NA	ABC transporter substrate-binding protein	GO:0006865, GO:0030288, GO:0055052
MMB18_RS36970	Pyrrolnitrin	NA	sugar ABC transporter permease	GO:0008643, GO:0042626, GO:0140359, GO:0016020, GO:0043190
MMB18_RS36975	Pyrrolnitrin	xylG	D-xylose ABC transporter ATP-binding protein	GO:0015753, GO:0005524, GO:0015614, GO:0016020
MMB18_RS36980	Pyrrolnitrin	xylF	D-xylose ABC transporter substrate-binding protein	GO:0015753, GO:0015614, GO:0033222, GO:0055052
MMB18_RS36985	Pyrrolnitrin	xylB	xylulokinase	GO:0005997, GO:0004856
MMB18_RS36990	Pyrrolnitrin	xylA	xylose isomerase	GO:0042732, GO:0009045
MMB18_RS36995	Pyrrolnitrin	NA	XylR family transcriptional regulator	GO:0006355, GO:0003700
MMB18_RS37000	Pyrrolnitrin	NA	aldose epimerase family protein	GO:0005975, GO:0016857, GO:0030246
MMB18_RS37005	Pyrrolnitrin	NA	glycoside hydrolase family 3 protein	GO:0005975, GO:0004553
MMB18_RS37010	Pyrrolnitrin	NA	Rieske 2Fe-2S domain-containing protein	GO:0051537
MMB18_RS37015	Pyrrolnitrin	NA	NAD(P)/FAD-dependent oxidoreductase	GO:0000166, GO:0016491, GO:0071949
MMB18_RS37020	Pyrrolnitrin	NA	monodechloroaminopyrrolnitrin synthase PrnB family protein	NA
MMB18_RS37025	Pyrrolnitrin	NA	tryptophan halogenase family protein	GO:0000166, GO:0016491
MMB18_RS37030	Pyrrolnitrin	NA	LysE family translocator	GO:0006865, GO:0016020

MMB18_RS37035	Pyrrolnitrin	NA	TetR/AcrR family transcriptional regulator	GO:0006355, GO:0003677, GO:0003700
MMB18_RS37040	Pyrrolnitrin	NA	hypothetical protein	NA
MMB18_RS37050	Pyrrolnitrin	NA	hypothetical protein	NA
MMB18_RS37055	Pyrrolnitrin	NA	hypothetical protein	NA
MMB18_RS37075	Pyrrolnitrin	NA	phosphatidylserine decarboxylase family protein	NA
MMB18_RS37095	Pyrrolnitrin	ligD	DNA ligase D	GO:0006303
MMB18_RS37105	Pyrrolnitrin	NA	DUF421 domain-containing protein	GO:0016020

Supplemental Table 3: The 138 B. contaminans genes that had relative fitness values greater than 2 (|log2FC\>2) and an adjusted p-value less than 0.01 for root and shoot groups when compared to the No Seed control.

Gene ID	Gene Name	Gene Product	Gene Ontology	Comparison	log2FC	padj
MMB18_RS00310	NA	type II secretion system protein N	NA	Root vs No Seed	-6.95	2.24E-03
				Shoot vs No Seed	-5.80	8.53E-03
MMB18_RS01245	NA	Mut7-C RNAse domain-containing protein	NA	Root vs No Seed	-5.99	5.08E-03
				Shoot vs No Seed	-6.21	2.65E-03
MMB18_RS02335	NA	HAD family hydrolase	GO:0016787	Root vs No Seed	-4.12	5.42E-03
				Shoot vs No Seed	-4.98	7.44E-04
MMB18_RS03700	purM	phosphoribosylformylglycinamidine cyclo-ligase	GO:0006189, GO:0004641	Root vs No Seed	-5.55	8.51E-03
				Shoot vs No Seed	-6.51	1.73E-03
MMB18_RS05230	NA	methionine ABC transporter ATP-binding protein	GO:0048473, GO:0005524, GO:0016887, GO:0033232, GO:0042626	Root vs No Seed	-2.15	7.55E-03
				Shoot vs No Seed	-2.55	1.25E-03
MMB18_RS05655	efp	elongation factor P	GO:0006414, GO:0003746, GO:0005737	Root vs No Seed	-6.67	7.99E-03
				Shoot vs No Seed	-7.01	3.22E-03
MMB18_RS08175	NA	ABC transporter permease	GO:0042626, GO:0140359	Root vs No Seed	-4.83	7.55E-03
				Shoot vs No Seed	-4.70	9.11E-03
MMB18_RS08350	NA	MbtH family protein	NA	Root vs No Seed	-10.60	3.75E-06
				Shoot vs No Seed	-5.64	4.96E-03
MMB18_RS09375	NA	cystathionine beta-lyase	GO:0019346, GO:0047804	Root vs No Seed	-4.94	1.27E-04
				Shoot vs No Seed	-3.98	2.57E-03
MMB18_RS10120	NA	SurA N-terminal domain-containing protein	NA	Root vs No Seed	-4.64	7.76E-03
				Shoot vs No Seed	-4.82	4.78E-03
MMB18_RS14315	NA	DeoR/GlpR family DNA-binding transcription regulator	GO:0003677, GO:0003700	Root vs No Seed	-6.87	4.96E-07
				Shoot vs No Seed	-5.58	9.25E-05
MMB18_RS14915	NA	tetratricopeptide repeat protein	NA	Root vs No Seed	-4.63	1.17E-04
				Shoot vs No Seed	-3.66	3.08E-03
MMB18_RS14925	ispE	4-(cytidine 5'-diphospho)-2-C-methyl-D-erythritol kinase	GO:0016114, GO:0050515	Root vs No Seed	-5.46	9.66E-04
				Shoot vs No Seed	-5.04	2.65E-03
MMB18_RS15245	gshB	glutathione synthase	GO:0006750, GO:0004363, GO:0005524, GO:0046872	Root vs No Seed	-5.71	2.00E-03
				Shoot vs No Seed	-7.51	5.28E-05
MMB18_RS16490	queC	7-cyano-7-deazaguanine synthase QueC	GO:0008616	Root vs No Seed	-6.57	4.23E-03
				Shoot vs No Seed	-6.76	2.30E-03
MMB18_RS16625	NA	putative 2-aminoethylphosphonate ABC transporter ATP-binding protein	GO:0033223, GO:0005524, GO:0033225, GO:0009898	Root vs No Seed	-4.90	5.46E-03
				Shoot vs No Seed	-6.23	3.05E-04
MMB18_RS19625	NA	DedA family protein/thiosulfate sulfurtransferase GlpE	NA	Root vs No Seed	-6.46	2.10E-04
				Shoot vs No Seed	-6.01	5.32E-04
MMB18_RS19975	NA	IclR family transcriptional regulator	GO:0006355, GO:0003677, GO:0003700	Root vs No Seed	-7.77	2.46E-06
				Shoot vs No Seed	-7.29	1.66E-05
MMB18_RS22130	NA	NAD(P)/FAD-dependent oxidoreductase	GO:0000166, GO:0050660, GO:0050661	Root vs No Seed	-5.52	7.77E-04
				Shoot vs No Seed	-4.89	3.66E-03
MMB18_RS22150	NA	polyprenyl synthetase family protein	GO:0008299, GO:0004659, GO:0046872	Root vs No Seed	-3.41	5.24E-03
				Shoot vs No Seed	-4.31	2.07E-04
MMB18_RS26645	shc	squalene--hopene cyclase	GO:0019746, GO:0016866, GO:0051007	Root vs No Seed	-2.80	4.51E-03
				Shoot vs No Seed	-4.27	7.54E-07
MMB18_RS35120	NA	DUF262 domain-containing protein	NA	Root vs No Seed	-6.94	1.59E-04
				Shoot vs No Seed	-5.53	3.03E-03
MMB18_RS35200	NA	hypothetical protein	NA	Root vs No Seed	5.57	1.68E-03
				Shoot vs No Seed	6.68	4.21E-05

MMB18_RS01315	NA	hypothetical protein	NA	Root vs No Seed	3.12	4.37E-03
MMB18_RS02150	tssG	type VI secretion system baseplate subunit TssG	GO:0033103	Root vs No Seed	4.82	7.55E-03
MMB18_RS02330	NA	cysteine hydrolase family protein	GO:0016787	Root vs No Seed	8.63	4.02E-09
MMB18_RS02430	NA	7-cyano-7-deazaguanine synthase	NA	Root vs No Seed	-4.95	4.23E-03
MMB18_RS02435	NA	hypothetical protein	NA	Root vs No Seed	-7.76	2.68E-04
MMB18_RS02510	ychF	redox-regulated ATPase YchF	GO:0005524, GO:0005525, GO:0016887	Root vs No Seed	5.14	7.85E-03
MMB18_RS03230	NA	LysR substrate-binding domain-containing protein	NA	Root vs No Seed	-6.53	6.36E-03
MMB18_RS03240	NA	glutamate/aspartate ABC transporter substrate-binding protein	GO:0001522, GO:0009451, GO:0003723, GO:0009982	Root vs No Seed	6.85	1.61E-04
MMB18_RS03290	NA	DUF1090 family protein	NA	Root vs No Seed	-10.86	3.23E-03
MMB18_RS04000	NA	type IV pilin protein	GO:0043683, GO:0016020	Root vs No Seed	7.41	1.21E-04
MMB18_RS04005	NA	DUF3318 domain-containing protein	NA	Root vs No Seed	6.12	7.55E-03
MMB18_RS04710	NA	Bcr/CflA family multidrug efflux MFS transporter	GO:0042908, GO:1990961, GO:0042910, GO:0016020	Root vs No Seed	-3.50	4.35E-03
MMB18_RS04765	NA	DNA translocase FtsK	GO:0007059, GO:0051301, GO:0003677, GO:0005524, GO:0015616	Root vs No Seed	-3.86	2.92E-03
MMB18_RS05340	pmbA	metalloprotease PmbA	GO:0006508, GO:0008237	Root vs No Seed	2.82	4.92E-04
MMB18_RS05580	rpoE	RNA polymerase sigma factor RpoE	GO:0006352, GO:0006355, GO:0003677, GO:0003700, GO:0016987	Root vs No Seed	-5.61	8.24E-03
MMB18_RS07000	bcsG	cellulose biosynthesis protein BcsG	GO:0030244	Root vs No Seed	5.52	2.96E-03
MMB18_RS07110	alaS	alanine--tRNA ligase	GO:0006419, GO:0000166, GO:0004813, GO:0005737	Root vs No Seed	9.94	1.74E-09
MMB18_RS07410	NA	alpha/beta fold hydrolase	NA	Root vs No Seed	-4.24	6.63E-03
MMB18_RS07630	NA	transposase	GO:0006310, GO:0015074, GO:0032196, GO:0003677, GO:0004803	Root vs No Seed	-4.90	7.76E-03
MMB18_RS09435	phaC	class I poly(R)-hydroxyalkanoic acid synthase	GO:0042619, GO:0016746, GO:0005737	Root vs No Seed	-4.62	7.85E-03
MMB18_RS10645	NA	[protein-PII] uridylyltransferase	GO:0008152, GO:0008773	Root vs No Seed	4.40	7.32E-03
MMB18_RS10985	NA	hypothetical protein	NA	Root vs No Seed	-6.24	2.96E-03
MMB18_RS11475	NA	IclR family transcriptional regulator	GO:0006355, GO:0003677, GO:0003700	Root vs No Seed	-4.93	3.45E-03
MMB18_RS12260	NA	TetR/AcrR family transcriptional regulator	NA	Root vs No Seed	6.24	1.44E-03
MMB18_RS12660	NA	EAL domain-containing protein	NA	Root vs No Seed	7.83	7.80E-05
MMB18_RS13185	NA	CysB family HTH-type transcriptional regulator	NA	Root vs No Seed	6.46	3.04E-03
MMB18_RS13270	NA	acetylornithine transaminase	NA	Root vs No Seed	-6.42	1.13E-04
MMB18_RS13650	NA	tRNA threonylcarbamoyladenosine dehydratase	GO:0008641	Root vs No Seed	7.14	7.77E-04
MMB18_RS15285	NA	sigma-54-dependent transcriptional regulator	GO:0000160, GO:0006355, GO:0000156, GO:0003677, GO:0005524	Root vs No Seed	6.10	8.24E-03
MMB18_RS16195	NA	PepSY-associated TM helix domain-containing protein	NA	Root vs No Seed	-2.51	3.22E-03
MMB18_RS16750	rnpA	ribonuclease P protein component	GO:0008033, GO:0000049, GO:0004526	Root vs No Seed	-7.70	2.65E-04
MMB18_RS17375	NA	autoinducer binding domain-containing protein	NA	Root vs No Seed	7.36	3.04E-14
MMB18_RS19045	NA	TonB-dependent receptor domain-containing protein	NA	Root vs No Seed	-3.83	9.83E-03

MMB18_RS19095	NA	YbaK/prolyl-tRNA synthetase associated domain-containing protein	NA	Root vs No Seed	-6.49	6.36E-03
MMB18_RS21750	trpB	tryptophan synthase subunit beta	GO:0000162, GO:0004834	Root vs No Seed	-4.35	4.78E-03
MMB18_RS21905	acnA	aconitate hydratase AcnA	GO:0006099, GO:0003994	Root vs No Seed	-6.39	7.77E-04
MMB18_RS21975	NA	TonB-dependent copper receptor	GO:0006825, GO:0005375, GO:0005507, GO:0019867	Root vs No Seed	5.12	7.77E-04
MMB18_RS22260	NA	CHAD domain-containing protein	NA	Root vs No Seed	4.62	8.24E-03
MMB18_RS25415	NA	TcfC E-set like domain-containing protein	NA	Root vs No Seed	-5.10	2.04E-05
MMB18_RS25705	NA	YadA-like family protein	NA	Root vs No Seed	3.52	6.36E-03
MMB18_RS25835	NA	AraC family transcriptional regulator	NA	Root vs No Seed	-4.95	3.25E-04
MMB18_RS31945	NA	hypothetical protein	NA	Root vs No Seed	-6.45	7.76E-03
MMB18_RS33300	NA	ABC transporter substrate-binding protein	NA	Root vs No Seed	7.26	8.49E-05
MMB18_RS34025	NA	MerR family transcriptional regulator	GO:0006355, GO:0003677, GO:0003700	Root vs No Seed	-5.52	8.51E-03
MMB18_RS35145	NA	family 1 encapsulin nanocompartment shell protein	GO:0140737	Root vs No Seed	-5.95	7.32E-04
MMB18_RS36045	NA	alkaline phosphatase D family protein	GO:0016787, GO:0046872	Root vs No Seed	-5.42	7.55E-03
MMB18_RS37000	NA	aldose epimerase family protein	GO:0005975, GO:0016857, GO:0030246	Root vs No Seed	6.65	7.77E-04
MMB18_RS00025	drmB	DUF1998 domain-containing protein	NA	Shoot vs No Seed	-5.42	8.05E-03
MMB18_RS00090	NA	23S ribosomal RNA	NA	Shoot vs No Seed	-3.75	1.94E-04
MMB18_RS00820	NA	hypothetical protein	NA	Shoot vs No Seed	-6.81	7.04E-04
MMB18_RS01390	NA	23S ribosomal RNA	NA	Shoot vs No Seed	-3.13	1.60E-03
MMB18_RS01980	NA	cytochrome b	GO:0022904, GO:0008121, GO:0009055, GO:0020037, GO:0046872	Shoot vs No Seed	-3.97	8.53E-03
MMB18_RS02220	NA	quinone oxidoreductase family protein	GO:0008270, GO:0016616, GO:0030554, GO:0048038	Shoot vs No Seed	-3.54	8.53E-03
MMB18_RS02630	NA	23S ribosomal RNA	NA	Shoot vs No Seed	-3.09	9.98E-04
MMB18_RS02795	NA	GspE/PulE family protein	GO:0009306, GO:0005524, GO:0016887, GO:0016020	Shoot vs No Seed	5.56	9.37E-03
MMB18_RS03055	NA	NADP-dependent malic enzyme	NA	Shoot vs No Seed	-3.50	7.12E-04
MMB18_RS03205	NA	GntP family permease	NA	Shoot vs No Seed	-4.99	4.20E-03
MMB18_RS03285	NA	OsmC family protein	NA	Shoot vs No Seed	-4.67	2.10E-03
MMB18_RS03320	dtd	D-aminoacyl-tRNA deacylase	GO:0008152, GO:0004045	Shoot vs No Seed	-7.24	5.01E-03
MMB18_RS03725	mutL	DNA mismatch repair endonuclease MutL	GO:0006298, GO:0005524, GO:0016887, GO:0030983, GO:0032300	Shoot vs No Seed	3.78	2.16E-03
MMB18_RS04370	NA	mannose-1-phosphate guanylyltransferase/mannose-6-phosphate isomerase	GO:0000271, GO:0016779	Shoot vs No Seed	-5.73	9.81E-03
MMB18_RS04520	urtD	urea ABC transporter ATP-binding protein UrtD	GO:0015840, GO:0005524, GO:0033221, GO:0009898, GO:0055052	Shoot vs No Seed	-2.17	1.82E-04
MMB18_RS05770	NA	ABC transporter ATP-binding protein	GO:0005524, GO:0016887, GO:0042626, GO:0140359, GO:0055052	Shoot vs No Seed	-5.19	5.44E-04
MMB18_RS06415	NA	LysR family transcriptional regulator	GO:0006355, GO:0003700	Shoot vs No Seed	-4.07	4.36E-03
MMB18_RS06975	NA	cellulose synthase subunit BcsC-related outer membrane protein	GO:0030244, GO:0019867	Shoot vs No Seed	-2.20	4.00E-03
MMB18_RS07005	NA	hypothetical protein	NA	Shoot vs No Seed	3.03	2.82E-03
MMB18_RS07370	NA	electron transfer flavoprotein-ubiquinone oxidoreductase	GO:0022900, GO:0004174, GO:0046872, GO:0051539	Shoot vs No Seed	-6.17	3.97E-09

MMB18_RS07490	bamC	outer membrane protein assembly factor BamC	NA	Shoot vs No Seed	-7.33	3.13E-03
MMB18_RS07620	lpdA	dihydrolipoyl dehydrogenase	GO:0004148, GO:0050660	Shoot vs No Seed	4.44	8.53E-03
MMB18_RS08125	lexA	transcriptional repressor LexA	GO:0009432, GO:0045892, GO:0004252	Shoot vs No Seed	-2.65	5.20E-03
MMB18_RS08185	rbsK	ribokinase	GO:0006014, GO:0004747	Shoot vs No Seed	-5.92	7.07E-03
MMB18_RS09185	NA	M949_RS01915 family surface polysaccharide biosynthesis protein	NA	Shoot vs No Seed	-6.34	4.36E-03
MMB18_RS09190	NA	sigma-54 interaction domain-containing protein	GO:0006355, GO:0005524, GO:0008134	Shoot vs No Seed	-4.44	5.95E-03
MMB18_RS09445	NA	RluA family pseudouridine synthase	GO:0001522, GO:0009451, GO:0003723, GO:0009982	Shoot vs No Seed	-3.15	4.62E-03
MMB18_RS09550	rlmN	23S rRNA (adenine(2503)-C(2))-methyltransferase RlmN	GO:0030488, GO:0070475, GO:0008757, GO:0016433	Shoot vs No Seed	-5.97	7.38E-05
MMB18_RS10110	NA	ABC transporter ATP-binding protein	GO:0005524, GO:0016887, GO:0042626, GO:0140359	Shoot vs No Seed	-2.49	8.32E-03
MMB18_RS11295	NA	class I SAM-dependent methyltransferase	GO:0032259, GO:0008168, GO:1904047	Shoot vs No Seed	4.38	2.16E-03
MMB18_RS11445	NA	IscS subfamily cysteine desulfurase	GO:0044571, GO:0030170, GO:0031071	Shoot vs No Seed	-7.39	1.49E-07
MMB18_RS11510	fixL	oxygen sensor histidine kinase FixL	NA	Shoot vs No Seed	-4.88	7.38E-05
MMB18_RS11585	NA	sterol desaturase family protein	GO:0006629, GO:0008610, GO:0016491	Shoot vs No Seed	-5.38	8.43E-04
MMB18_RS11960	NA	23S ribosomal RNA	NA	Shoot vs No Seed	-3.64	2.36E-04
MMB18_RS12160	NA	NAD(P)H-quinone oxidoreductase	GO:0003960, GO:0048038, GO:0070402	Shoot vs No Seed	-5.28	9.58E-03
MMB18_RS12420	NA	FUSC family protein	GO:0055085, GO:0022857, GO:0005886	Shoot vs No Seed	-3.55	8.32E-03
MMB18_RS12735	NA	TetR/AcrR family transcriptional regulator	GO:0006355, GO:0003677, GO:0003700	Shoot vs No Seed	-5.12	6.97E-04
MMB18_RS13705	kynA	tryptophan 2,3-dioxygenase	GO:0019441, GO:0004833	Shoot vs No Seed	-10.34	3.24E-05
MMB18_RS14530	nudB	dihydroneopterin triphosphate diphosphatase	GO:0046656, GO:0008828, GO:0019177	Shoot vs No Seed	3.96	1.65E-03
MMB18_RS15120	NA	COX15/CtaA family protein	GO:0006783, GO:0017004, GO:0016491, GO:0020037, GO:0046872	Shoot vs No Seed	-4.67	5.03E-03
MMB18_RS15170	NA	DUF2244 domain-containing protein	NA	Shoot vs No Seed	-5.31	5.45E-03
MMB18_RS15280	NA	peroxiredoxin family protein	GO:0051920	Shoot vs No Seed	-5.92	9.66E-03
MMB18_RS15580	NA	dienelactone hydrolase family protein	GO:0016787	Shoot vs No Seed	-7.16	7.72E-06
MMB18_RS15630	NA	indolepyruvate ferredoxin oxidoreductase family protein	NA	Shoot vs No Seed	-4.84	7.90E-04
MMB18_RS19890	NA	cytochrome P450	GO:0004497, GO:0005506, GO:0016705, GO:0020037	Shoot vs No Seed	2.10	3.67E-03
MMB18_RS20825	NA	helix-hairpin-helix domain-containing protein	NA	Shoot vs No Seed	2.46	4.11E-03
MMB18_RS20870	NA	HpnL family protein	NA	Shoot vs No Seed	-4.27	1.43E-03
MMB18_RS21270	NA	putative bifunctional diguanylate cyclase/phosphodiesterase	GO:0005525, GO:0046872, GO:0052621, GO:0071111, GO:0005886	Shoot vs No Seed	2.01	7.66E-03
MMB18_RS24680	NA	ATP-binding protein	GO:0005524	Shoot vs No Seed	2.15	9.11E-03
MMB18_RS26660	NA	MMPL family transporter	GO:0016020	Shoot vs No Seed	-2.99	3.25E-03
MMB18_RS27655	NA	LysR substrate-binding domain-containing protein	NA	Shoot vs No Seed	2.46	8.53E-03
MMB18_RS28645	NA	23S ribosomal RNA	NA	Shoot vs No Seed	-2.98	2.08E-03
MMB18_RS29525	NA	alpha/beta hydrolase	GO:0016787	Shoot vs No Seed	2.93	4.36E-03
MMB18_RS29535	NA	DinB family protein	NA	Shoot vs No Seed	4.50	8.33E-03
MMB18_RS29915	NA	thiolase family protein	GO:0016746	Shoot vs No Seed	4.19	3.05E-04
MMB18_RS30170	NA	SymE family type I addiction module toxin	GO:0016070, GO:0003723, GO:0016788, GO:0005737	Shoot vs No Seed	-5.78	5.51E-03

MMB18_RS30590	NA	hypothetical protein	NA	Shoot vs No Seed	3.26	4.53E-03
MMB18_RS31475	NA	2-keto-4-pentenoate hydratase	GO:0008684, GO:0030145	Shoot vs No Seed	5.34	8.32E-03
MMB18_RS31790	NA	electron transfer flavoprotein-ubiquinone oxidoreductase	GO:0022900, GO:0004174, GO:0046872, GO:0051539	Shoot vs No Seed	-5.64	1.94E-04
MMB18_RS32785	NA	LLM class flavin-dependent oxidoreductase	GO:0010181, GO:0016491	Shoot vs No Seed	3.02	1.18E-04
MMB18_RS33195	NA	sugar transferase	NA	Shoot vs No Seed	2.23	7.13E-03
MMB18_RS33925	NA	MFS transporter	GO:0055085, GO:0022857	Shoot vs No Seed	7.74	9.74E-04
MMB18_RS34570	NA	methyl-accepting chemotaxis protein	GO:0007165, GO:0016020	Shoot vs No Seed	2.14	3.21E-03
MMB18_RS35340	NA	site-specific integrase	NA	Shoot vs No Seed	2.88	4.56E-03
MMB18_RS35880	NA	electron transfer flavoprotein-ubiquinone oxidoreductase	GO:0022900, GO:0004174, GO:0046872, GO:0051539	Shoot vs No Seed	-4.62	5.32E-04
MMB18_RS37495	NA	electron transfer flavoprotein-ubiquinone oxidoreductase	GO:0022900, GO:0004174, GO:0046872, GO:0051539	Shoot vs No Seed	-5.33	1.23E-04
MMB18_RS37775	NA	23S ribosomal RNA	NA	Shoot vs No Seed	-3.55	2.96E-04
MMB18_RS37840	NA	hypothetical protein	NA	Shoot vs No Seed	2.86	8.53E-03

Supplemental Table 4: The 89 B. contaminans genes that had relative fitness values greater than 2 (|log2FC\>2) and an adjusted p-value less than 0.01 for the FOL challenged root group when compared to the unchallenged root group.

Gene ID	Gene Name	Gene Product	Gene Ontology	log2FC	Relative Fitness	padj
MMB18_RS07930	NA	NUDIX hydrolase	GO:0009132, GO:0016817, GO:0046872	-19.82	19.82	1.08E-10
MMB18_RS04580	NA	tRNA-Gly	NA	-9.51	9.51	3.61E-04
MMB18_RS21035	NA	LysR substrate-binding domain-containing protein	NA	-7.34	7.34	1.71E-05
MMB18_RS17375	NA	autoinducer binding domain-containing protein	NA	-6.39	6.39	2.02E-10
MMB18_RS21900	NA	bifunctional 2-methylcitrate dehydratase/aconitate hydratase	GO:0019679, GO:0047547, GO:0051537	-6.36	6.36	1.55E-03
MMB18_RS03655	dnaK	molecular chaperone DnaK	GO:0006457, GO:0005524, GO:0016887	-6.34	6.34	2.61E-03
MMB18_RS07110	alaS	alanine--tRNA ligase	GO:0006419, GO:0000166, GO:0004813, GO:0005737	-6.23	6.23	4.52E-04
MMB18_RS02510	ychF	redox-regulated ATPase YchF	GO:0005524, GO:0005525, GO:0016887	-5.63	5.63	2.37E-03
MMB18_RS15540	NA	SDR family oxidoreductase	GO:0016491	-5.34	5.34	9.98E-03
MMB18_RS33300	NA	ABC transporter substrate-binding protein	NA	-5.24	5.24	4.09E-03
MMB18_RS04660	NA	UbiD family decarboxylase	GO:0016831	-4.83	4.83	8.23E-03
MMB18_RS16585	NA	CGNR zinc finger domain-containing protein	GO:0003676, GO:0008270	-4.61	4.61	7.45E-03
MMB18_RS02330	NA	cysteine hydrolase family protein	GO:0016787	-4.57	4.57	3.01E-03
MMB18_RS15140	NA	cytochrome c oxidase subunit 3	GO:0009060, GO:0019646, GO:0004129, GO:0009055	-4.26	4.26	9.49E-03
MMB18_RS10645	NA	[protein-PII] uridylyltransferase	GO:0008152, GO:0008773	-4.25	4.25	4.53E-03
MMB18_RS32785	NA	LLM class flavin-dependent oxidoreductase	GO:0010181, GO:0016491	2.02	-2.02	4.91E-03
MMB18_RS33195	NA	sugar transferase	NA	2.06	-2.06	8.19E-03
MMB18_RS37540	NA	pyruvate carboxylase	GO:0006090, GO:0006094, GO:0004736, GO:0005524, GO:0009374, GO:0046872	2.08	-2.08	2.65E-03
MMB18_RS37950	gcvA	transcriptional regulator GcvA	GO:0006355, GO:0003700	2.13	-2.13	8.39E-03
MMB18_RS20825	NA	helix-hairpin-helix domain-containing protein	NA	2.19	-2.19	2.33E-03
MMB18_RS28220	NA	MFS transporter	GO:0055085, GO:0022857	2.21	-2.21	2.82E-03
MMB18_RS17650	NA	IclR family transcriptional regulator	GO:0006355, GO:0003677, GO:0003700	2.30	-2.30	6.24E-04
MMB18_RS17845	NA	GNAT family N-acetyltransferase	NA	2.34	-2.34	5.36E-03
MMB18_RS37840	NA	hypothetical protein	NA	2.44	-2.44	9.46E-03
MMB18_RS05005	pepN	aminopeptidase N	GO:0006508, GO:0004177, GO:0008237, GO:0008270	2.51	-2.51	3.43E-04
MMB18_RS22630	andAa	anthranilate 1,2-dioxygenase system ferredoxin--NAD(+) reductase	GO:0009056, GO:0008860	2.51	-2.51	4.48E-03
MMB18_RS20055	NA	phenylacetaldoxime dehydratase family protein	NA	2.57	-2.57	2.82E-03
MMB18_RS18740	NA	DHA2 family efflux MFS transporter permease subunit	GO:0006855, GO:0022857, GO:0016020	2.60	-2.60	3.26E-05
MMB18_RS32720	NA	diaminobutyrate--2-oxoglutarate transaminase family protein	GO:0019491, GO:0008483	2.69	-2.69	5.50E-03
MMB18_RS02235	NA	LEA type 2 family protein	NA	2.69	-2.69	8.73E-06
MMB18_RS14335	glpK	glycerol kinase GlpK	GO:0006072, GO:0004370	2.70	-2.70	8.05E-03
MMB18_RS23960	NA	curli assembly protein CsgF	NA	2.70	-2.70	5.43E-03
MMB18_RS29150	NA	choline ABC transporter substrate-binding protein	NA	2.70	-2.70	8.36E-03
MMB18_RS28110	NA	GlxA family transcriptional regulator	GO:0006355, GO:0003677, GO:0003700	2.73	-2.73	4.80E-04
MMB18_RS33475	NA	DEAD/DEAH box helicase	GO:0003676, GO:0005524, GO:0016887	2.73	-2.73	7.93E-03

MMB18_RS27655	NA	LysR substrate-binding domain-containing protein	NA	2.79	-2.79	9.64E-04
MMB18_RS17860	NA	MDR family MFS transporter	GO:0055085, GO:0022857, GO:0016020	3.03	-3.03	7.26E-03
MMB18_RS23050	NA	glycosyltransferase	GO:0006486, GO:0016757	3.05	-3.05	4.49E-03
MMB18_RS26360	epsC	serine O-acetyltransferase EpsC	GO:0006535, GO:0009001	3.16	-3.16	1.92E-03
MMB18_RS36945	NA	ABC transporter permease	GO:0042626, GO:0140359, GO:0016020, GO:0043190	3.17	-3.17	6.23E-05
MMB18_RS07225	NA	CoA-acylating methylmalonate-semialdehyde dehydrogenase	NA	3.22	-3.22	7.59E-04
MMB18_RS21715	purF	amidophosphoribosyltransferase	GO:0009113, GO:0004044	3.25	-3.25	8.35E-03
MMB18_RS21480	NA	ornithine decarboxylase	GO:0006596, GO:0016831	3.38	-3.38	1.71E-05
MMB18_RS26960	NA	3-deoxy-7-phosphoheptulonate synthase	GO:0009073, GO:0016832	3.51	-3.51	4.47E-03
MMB18_RS03180	NA	glutamate-5-semialdehyde dehydrogenase	GO:0006561, GO:0004350, GO:0016620	3.61	-3.61	9.56E-05
MMB18_RS07605	typA	translational GTPase TypA	GO:0006412, GO:0006950, GO:0003924, GO:0005525	3.92	-3.92	3.97E-03
MMB18_RS04010	NA	phage holin family protein	GO:0016020	4.02	-4.02	7.81E-03
MMB18_RS07880	rlmB	23S rRNA (guanosine(2251)-2'-O)-methyltransferase RlmB	GO:0006396, GO:0008173	4.06	-4.06	4.82E-04
MMB18_RS15815	ttcA	tRNA 2-thiocytidine(32) synthetase TtcA	GO:0008033	4.10	-4.10	4.48E-03
MMB18_RS34965	treS	maltose alpha-D-glucosyltransferase	GO:0005991, GO:0047471	4.16	-4.16	8.00E-05
MMB18_RS14695	NA	M48 family metallopeptidase	GO:0004222, GO:0008237, GO:0046872, GO:0016020	4.22	-4.22	5.20E-03
MMB18_RS14645	NA	alpha/beta hydrolase	GO:0016787	4.29	-4.29	4.20E-03
MMB18_RS12905	NA	heme biosynthesis protein HemY	NA	4.29	-4.29	4.97E-03
MMB18_RS24660	NA	TetR/AcrR family transcriptional regulator	GO:0006355, GO:0003677, GO:0003700	4.38	-4.38	5.88E-03
MMB18_RS11105	NA	hypothetical protein	NA	4.38	-4.38	3.48E-04
MMB18_RS03150	dapB	4-hydroxy-tetrahydrodipicolinate reductase	GO:0009089, GO:0008839	4.42	-4.42	4.54E-03
MMB18_RS14175	NA	SbcC/MukB-like Walker B domain-containing protein	NA	4.46	-4.46	6.22E-04
MMB18_RS09465	NA	hypothetical protein	NA	4.55	-4.55	6.65E-04
MMB18_RS15960	NA	transcriptional regulator GcvA	GO:0006351, GO:0006355, GO:0003677	4.81	-4.81	7.93E-03
MMB18_RS00305	NA	efflux transporter outer membrane subunit	GO:0006810, GO:0015288, GO:0009279	4.83	-4.83	4.48E-03
MMB18_RS10860	NA	C40 family peptidase	GO:0000270, GO:0006508, GO:0008233, GO:0008234, GO:0016787	4.85	-4.85	1.63E-03
MMB18_RS20600	pcaQ	pca operon transcription factor PcaQ	GO:0019619, GO:0045893, GO:0003677	4.87	-4.87	3.48E-03
MMB18_RS21720	NA	CvpA family protein	GO:0009403	5.04	-5.04	4.90E-03
MMB18_RS21905	acnA	aconitate hydratase AcnA	GO:0006099, GO:0003994	5.06	-5.06	6.39E-03
MMB18_RS05520	NA	S49 family peptidase	NA	5.07	-5.07	2.26E-03
MMB18_RS20705	NA	metallophosphoesterase family protein	GO:0016787, GO:0042578, GO:0046872	5.26	-5.26	2.33E-03
MMB18_RS00845	NA	helix-turn-helix domain-containing protein	GO:0003677	5.43	-5.43	9.98E-03
MMB18_RS35375	NA	hypothetical protein	NA	5.45	-5.45	1.79E-04
MMB18_RS01985	NA	cytochrome c1	GO:0009055, GO:0020037, GO:0046872	5.47	-5.47	1.54E-03
MMB18_RS38795	NA	b(o/a)3-type cytochrome-c oxidase subunit 1	NA	5.50	-5.50	9.15E-03
MMB18_RS15885	NA	glutamine synthetase family protein	GO:0006542, GO:0004356, GO:0005524, GO:0046872	5.54	-5.54	4.48E-03
MMB18_RS13495	NA	EAL domain-containing protein	GO:0071111	5.56	-5.56	3.26E-05
MMB18_RS03585	aroG	3-deoxy-7-phosphoheptulonate synthase AroG	GO:0009073, GO:0003849	5.76	-5.76	1.29E-03

MMB18_RS15740	NA	alpha/beta fold hydrolase	NA	5.85	-5.85	5.00E-03
MMB18_RS05320	NA	M48 family metallopeptidase	GO:0006508, GO:0004222, GO:0046872	5.88	-5.88	1.39E-03
MMB18_RS01170	NA	c-type cytochrome	GO:0009055, GO:0020037, GO:0046872	5.91	-5.91	4.09E-03
MMB18_RS38810	NA	IS3-like element ISBmu11 family transposase	GO:0006313, GO:0004803	6.10	-6.10	3.48E-04
MMB18_RS14340	NA	MIP/aquaporin family protein	GO:0055085, GO:0015267	6.19	-6.19	1.27E-03
MMB18_RS23255	irlR	heavy metal response regulator transcription factor IrlR	NA	6.21	-6.21	6.63E-03
MMB18_RS05245	NA	electron transfer flavoprotein subunit beta/FixA family protein	GO:0009055	6.28	-6.28	6.22E-06
MMB18_RS05470	rmuC	DNA recombination protein RmuC	NA	6.29	-6.29	2.73E-03
MMB18_RS25180	NA	LysR family transcriptional regulator	GO:0006355, GO:0003700	6.31	-6.31	3.97E-03
MMB18_RS29635	betI	transcriptional regulator BetI	GO:0003677	6.38	-6.38	9.24E-05
MMB18_RS22320	NA	RES family NAD+ phosphorylase	NA	6.52	-6.52	3.40E-03
MMB18_RS07115	NA	LysR family transcriptional regulator	GO:0001216, GO:0003677, GO:0003700	6.93	-6.93	4.82E-04
MMB18_RS33925	NA	MFS transporter	GO:0055085, GO:0022857	6.97	-6.97	2.61E-03
MMB18_RS07920	NA	arginyltransferase	GO:0016598, GO:0004057	6.99	-6.99	8.85E-03
MMB18_RS05120	gph	phosphoglycolate phosphatase	GO:0005975, GO:0008967	7.14	-7.14	6.75E-04
MMB18_RS38825	NA	hypothetical protein	NA	7.35	-7.35	1.54E-03
