# Supplementary figures and images for: Transposon sequencing reveals Burkholderia gene fitness in a spaceflight-relevant plant-pathogen interaction
Source: Appl Environ Microbiol. 2026 Jan 13;92(2):e01941-25. doi: 10.1128/aem.01941-25 (PMC12915293; doi:10.1128/aem.01941-25)

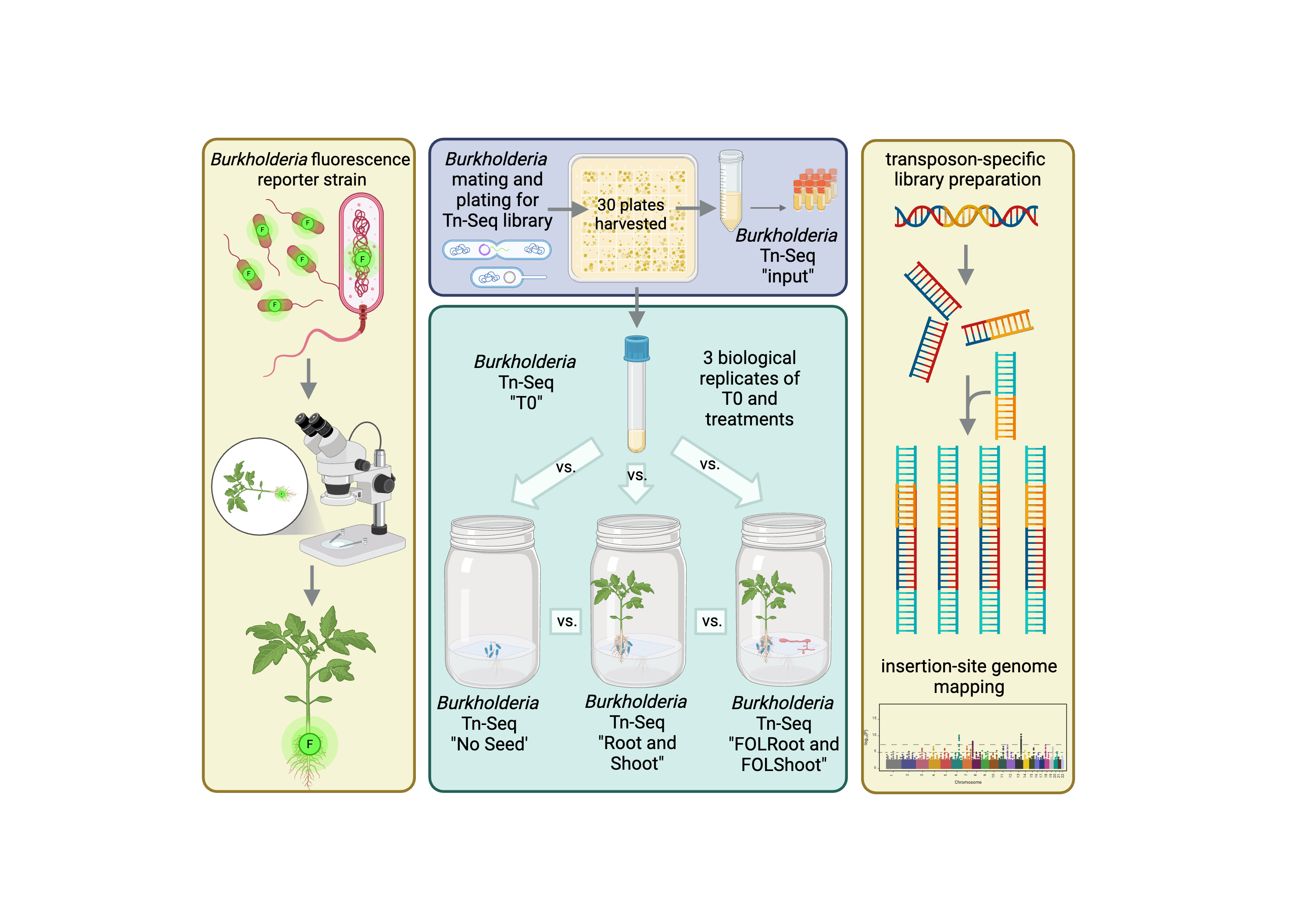

Supplement: Graphical abstract — Study overview. [file aem.01941-25-s0002.tiff]
